# The Prognostic Significance and Potential Mechanism of Prolyl 3-Hydroxylase 1 in Hepatocellular Carcinoma

**DOI:** 10.1155/2022/7854297

**Published:** 2022-10-26

**Authors:** Chunlei Li, Lilong Zhang, Yao Xu, Dongqi Chai, Shengchen Nan, Zhendong Qiu, Weixing Wang, Wenhong Deng

**Affiliations:** ^1^Department of General Surgery, Renmin Hospital of Wuhan University, No.238, Jiefang Road, Wuchang District, Wuhan 430060, Hubei Province, China; ^2^Hubei Key Laboratory of Digestive System Disease, No.238, Jiefang Road, Wuchang District, Wuhan 430060, Hubei Province, China; ^3^Department of Critical Care Medicine, Affiliated Jinling Hospital, Medical School of Nanjing University, Nanjing, China

## Abstract

**Background:**

Prolyl 3-hydroxylase 1 (P3H1) is essential for human collagen synthesis. Here, we investigated its relevance to multiple cancers, especially hepatocellular carcinoma (LIHC).

**Methods:**

We estimated the relationship of P3H1 with 33 cancers using publicly available databases. And immunohistochemistry was utilized to verify the P3H1 expression in liver, gastric, colon, pancreatic, and rectal cancer. Then, we attenuated P3H1 expression in BEL-7402 and HLF cells by lentivirus technology and assessed the effect of P3H1 on cell proliferation, migration, and invasion.

**Results:**

Bioinformatic analysis revealed a significantly higher expression of P3H1 in almost all tumors, which was consistent with the immunohistochemical findings in the liver, gastric, colon, pancreatic, and rectal cancers. P3H1 expression was associated with overall survival, progression-free interval, disease-specific survival, and disease-free interval in most cancers, particularly in LIHC. Besides, we also found that P3H1 expression was an independent prognostic factor for LIHC. And knockdown of P3H1 significantly reduced liver cancer cell proliferation, migration, and invasion in liver cancer cells. Interestingly, P3H1 expression levels showed a significant positive connection with Th2 infiltration through multiple immune infiltration algorithms. ICI treatment was less effective in LIHC patients with high P3H1 expression. Finally, we also identified an upstream regulatory mechanism of P3H1 in LIHC, namely, AL355488.1, HCG18, and THUMPD3-AS1/hsa-miR-29c-3p-P3H1 axis.

**Conclusion:**

We have systematically described for the first time that P3H1 is closely related to various tumors, particularly in LIHC, and interference with P3H1 may be a therapeutic target for patients with LIHC.

## 1. Introduction

Collagens represent a large family of structural proteins, accounting for more than 30% of the total body protein in humans [1]. Different types of collagens make up a wide variety of supersized molecules that are highly ordered and assembled in the extracellular matrix [2]. To date, 28 types of collagens have been reported, of which type I is the most abundant. It accounts for over 90% of all collagen and is widely expressed in most connective tissues, including skin, bone, and blood vessels. Prolyl 3-hydroxylase (P3H) is primarily involved in the synthesis of collagen with specific 3-hydroxylation of the proline at the X-position in the collagen Gly-X-Y repeat sequence [3]. The P3H family has three isozymes: P3H1, P3H2, and P3H3, as well as two potential coenzymes: cartilage-associated protein (CRTAP) and synaptonemal complex 65 (Sc65) [4]. These enzymes are localized in the endoplasmic reticulum and are required for collagen synthesis and assembly [3]. Of these, P3H1 is responsible for hydroxylation of Pro986 on the alpha1 chain and the Pro707 site on the alpha2 chain of type I collagen [5].

Prolyl 3-hydroxylase 1 (P3H1) is also known as leucine proline-enriched proteoglycan (leprecan) 1 (LEPRE1), growth suppressor 1 (GROS1), or procollagen-proline3-dioxygenase. Previous studies have identified three independent biological functions of P3H1. As leprecan, it can regulate intracellular pathways such as endoplasmic reticulum signaling and cell-matrix interactions [6]. In humans, P3H1 serves as a growth inhibitor located on chromosome 1 [7]. Finally, P3H1, as a member of the 2-oxoglutarate dioxygenase family, plays a crucial role in collagen synthesis, folding, and assembly [3, 8]. Huang et al. recently revealed that P3H1 was remarkably upregulated in osteosarcoma tissues and cell lines (MG63 and Saos2), while knockdown of P3H1 inhibited proliferation, migration, and invasion of osteosarcoma [9].

**Figure 1:**
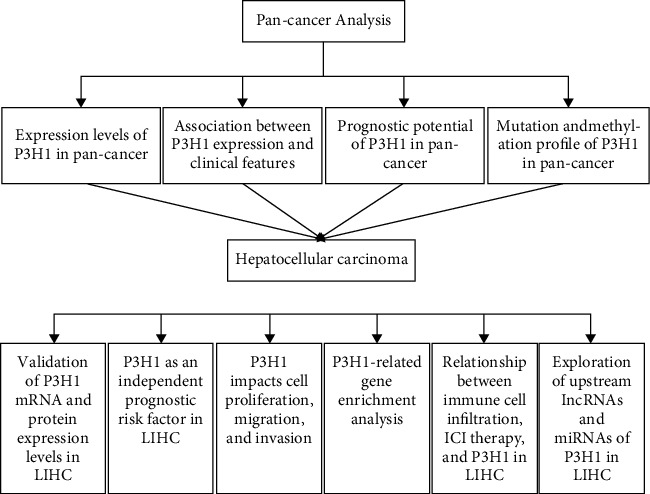
The workflow of the analysis steps. ICI, immune checkpoint inhibitor; LIHC, hepatocellular carcinoma.

In this study, bioinformatic analysis was performed to estimate the P3H1 expression in 33 cancers and its possible link to cancer, which was further verified by immunohistochemistry and in vitro experiments, to better understand the importance of P3H1 in multiple cancers, especially in LIHC (Figure 1).

## 2. Materials and Methods

### 2.1. Data Collection and Processing

The GTEx and TCGA data were obtained using the Xena Browser. The P3H1 expression in cancer cell lines was acquired using the CCLE database. The P3H1 mutation levels in various human malignancies were assessed using the cBioPortal database and the COSMIC database [10–12]. The EWAS Data Hub was used to explore the relationship between P3H1 methylation and patient prognosis [13]. The GSCA website was used to explore the association between P3H1 copy number variation and tumor prognosis [14]. GSE39791, GSE45267, GSE55092, GSE84598, GSE102079, and GSE121248, the Liver Cancer-RIKEN and JP (LIRI-JP) datasets were downloaded via GEO and the ICGC Data Portal. The HCCDB was used to validate the P3H1 expression in LIHC [15]. The starBase database was used for the prediction and analysis of lncRNAs and miRNAs upstream of P3H1 [16].

### 2.2. Expression Analysis of P3H1

The *R* software packages “gganatogram” and “ggpubr” were used to investigate P3H1 expression in normal tissues. The number of normal samples per tumor was counted for all TCGA data, and tumors with no less than five normal samples were chosen. A comparison between the two groups was conducted using the Wilcox test. The Kruskal–Wallis test was used to compare the three groups. The *R* software packages “ggpubr” and “ggplot2” were implemented to create the box plot. The paired difference analysis using the paired Wilcox test. Correlations among lncRNAs, miRNAs, and P3H1 were also evaluated using Pearson's correlation test.

### 2.3. Cox Regression Analysis and Survival Analysis

TCGA phenotypic and survival data were used to explore the relationship between P3H1 expression and overall survival (OS), disease-specific survival (DSS), disease-free interval (DFI), and progression-free interval (PFI). These patients were classified into high- and low-expression groups based on P3H1 median expression levels. The Kaplan–Meyer method was used to examine the difference in survival using the *R* packages “survival” and “survminer.” The *R* software packages “survival” and “forestplot” were used to calculate the hazard ratio using the Cox proportional hazard regression analysis.

### 2.4. Nomogram Establishment and Evaluation

Using the “rms” and “survival” *R* software packages, a nomogram was built to predict the 1-, 2-, and 5-year OS, PFI, and DSS in the TCGA-LIHC cohort. Then, calibration plots were then constructed based on the number of samples per group for repeated calculations (100), the number of repeated calculations (1000), and the method (boot).

### 2.5. Immune Cell Infiltration Analysis

“GSVA” *R* software package-based ssGSEA algorithm to calculate the difference in immune infiltration levels between LIHC patients with high P3H1 expression and low P3H1 expression (median value is the cut-off). Analysis of differences between the two groups was performed using the Wilcox test. We also analyzed the relationship between the expression of P3H1 and the infiltration of immune cells in the tumor microenvironment using the XCELL algorithms in the TIMER 2.0 platform. Finally, the “Immune Scores” template [17] from the CAMOIP website [18] was further used to analyze differences in immune infiltration and immune subtypes in LIHC patients with high and low P3H1 (median value is the cut-off).

### 2.6. Evaluation of the Efficacy of Immune Checkpoint Inhibitor

We obtained comprehensive immunogenomic analyses for LIHC data from the TCIA website. Patients were divided into high- and low-expression groups based on median P3H1 expression values and immune checkpoint inhibitor (ICI) treatment scores were assessed between the two groups.

### 2.7. KEGG and GO Enrichment Analysis

We divided the TCGA-LIHC cohort patients into high- and low-expression groups based on median P3H1 gene expression values. Differences in gene expression between the two groups were analyzed using the “limma” *R* package (logFCfilter = 1 and fdrFilter = 0.05), and heatmaps were created using the “pheatmap” *R* package. Next, we conducted KEGG and GO enrichment analysis of the above genes using the *R* packages “org.Hs.eg.db,” “DOSE,” “clusterProfiler,” “enrichplot,” “RColorBrewer,” “ggplot2,” and “stringi.”

### 2.8. Immunohistochemical Staining

The tumors and paracancerous tissues (LIHC, 50 cases; COAD, 20 cases; READ, 20 cases; STAD, 20 cases; and PAAD, 20 cases) were collected at our hospital (Wuhan). These tumors were subjected to immunohistochemistry analysis. Briefly, paraffin sections were baked, dewaxed, hydrated, and then subjected to antigen retrieval using sodium citrate buffer. Then, the slides were blocked for 15 min with a 3% hydrogen peroxide solution and then for 60 min with a 3% albumin bovine V. Anti-P3H1 antibody (rabbit, Proteintech, 1 : 400) was incubated overnight at 4°C on the sections, followed by a second antibody. The staining results were visualized using 3,5-diaminobenzidine. All slides were measured and photographed blindly using a light microscope. The AOD (integrated optical density/area) of the immunostained sections was measured quantitatively using the Image-Pro Plus 6.0 software (Media Cybernetics Inc, Bethesda, USA). The median value was used to determine whether a group had high or low expression.

### 2.9. Western Blotting

The RIPA buffer containing 1% protease (Roche) and 1% phosphatase inhibitor cocktail (Sigma) was used to lyse cells or tissues. The protein samples were separated by SDS-PAGE and transferred onto PVDF membranes (Millipore). After blocking with 5% skim milk, the membranes were probed with the anti-P3H1 antibody (rabbit, Proteintech, 1 : 1000) and then exposed to horseradish peroxidase (HRP)-linked secondary antibodies. The Bio-Rad GelDoc system was utilized to collect western blot pictures. ImageJ was used for semiquantitative analysis. Relative protein levels were calculated by using an internal reference GAPDH (rabbit, Abcam, 1 : 1000).

### 2.10. Cell Proliferation, Migration, and Invasion

The 293T cell line and liver cancer cell lines (BEL-7402 and HLF cells) were cultured at 37°C with 5% CO2 in Dulbecco's modified Eagle's medium (DMEM) containing 10% fetal bovine serum (FBS), 100 U/mL penicillin, and 100 g/mL streptomycin. For lentivirus production, pLV2-U6-P3H1 (human)-shRNA1-PGK-EGFP-Puro (8 *µ*g), psPAX2 (6 *µ*g), and pMD2 G (2 *µ*g) were purchased from MiaoLing Plasmid Platform (Wuhan, China) and were cotransfected into 293T cells. At 48 hours after transfection, the virus-containing supernatants were collected and added to exponentially growing BEL-7402 and HLF cells. P3H1 stable knockdown cells were achieved by 2-day puromycin (2 *µ*g/mL, Cayman Chemical, USA) selection. Cell proliferation was estimated using the Cell Counting Kit-8 (CCK-8, Dojindo, Tokyo, Japan). The 10 *μ*L CCK-8 solution was added to 96-well plates and incubated at 37°C for 1 h. The optical density was measured at 450 nm using an enzyme immunoassay (Beijing Zhongyi Technology Co., Ltd., Beijing, China). After the 6-well plate was inoculated with an equal number of cells, when the cell confluence was close to 100%, a 200 *μ*L pipette tip was used to create a wound in a single layer of cells. Images were obtained under a 40-fold inverted microscope and photographed at 0 and 24 h. The experiment was repeated three times. The 24-well transwell chambers with 8 *μ*m pore size polycarbonate membranes (Corning, USA) were used to test the invasion and migration of BEL-7402 and HLF cells. 100 *μ*L cell suspensions without FBS were added to the upper chamber coated with or without 90 *μ*L Matrigel (BD, USA), and 600 *µ*L of DMEM containing 10% FBS was added to the lower chamber. After 24 h (for migration) and 48 h (for invasion) of culture at 37°C in a 5% CO_2_ incubator, the chambers were fixed with 4% paraformaldehyde for 30 min and stained with a 0.1% crystal violet solution for 20 min.

Table S1 provides the database sites.  Table 1 provides the full cancer type name.

## 3. Results

### 3.1. Expression Levels of P3H1 in Pan-Cancer

**Figure 2:**
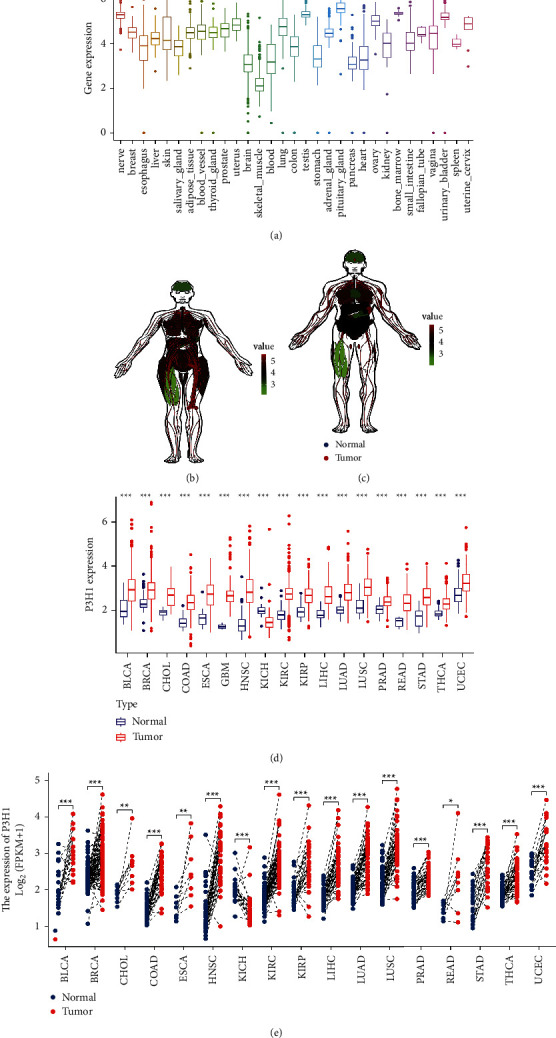
mRNA expression of the P3H1 gene. (a) The P3H1 expression in normal human tissues using the GTEx data. (b) Female and male (c) expression anatomy diagram using the GTEx data. (d) P3H1 expression in cancer and normal tissues using the TCGA data. (e) The pairwise difference analysis of various cancers using the TCGA data (^*∗*^*p* < 0.05, ^*∗∗*^*p* < 0.01, ^*∗∗∗*^*p* < 0.001).

First, we analyzed P3H1 expression in 31 different types of normal tissues using GTEx data. We found that the expression of P3H1 was higher in the pituitary gland, testis, bone marrow, spleen, and nerve, and lower in the skeletal muscle, brain, blood, and pancreas (Figure 2(a)). Furthermore, males had much higher P3H1 expression in the esophagus and spleen than females, whereas P3H1 exhibited an opposite expression profile in the thyroid gland (Figures 2(b) and 2(c), Figure S1A). The expression of P3H1 in various cancer cell lines was estimated using the CCLE database. The results showed that P3H1 was expressed in all 38 kinds of tumor cell lines at different expression levels. Specifically, the highest levels of P3H1 were observed in chondrosarcoma and giant cell tumor cell lines, while colorectal and bile duct cell lines showed the lowest levels (Figure S1B). Finally, we compared the difference in P3H1 expression between tumor tissues and adjacent nontumor tissue using the TCGA data. After selecting tumors with a number of nontumor tissue sample sizes ≥5, we found that P3H1 expression was significantly upregulated in BLCA, BRCA, CHOL, COAD, ESCA, GBM, HNSC, KIRC, KIRP, LUAD, LIHC, LUSC, PRAD, READ, STAD, THCA, and UCEC compared to normal tissues, while downregulated in KICH (Figure 2(d)). Results from pairwise difference analysis were consistent with the above findings (Figure 2(e)).

Because of the small number of normal tissues in the TCGA database, we further combined the GTEx database and the TCGA database. Compared with normal tissues, significantly higher expressions of P3H1 were observed in ACC, BLCA, BRCA, CHOL, COAD, DLBC, ESCA, GBM, HNSC, KIRC, KIRP, KIRC, LGG, LIHC, PAAD, READ, SKCM, STAD, THYM, and UCS, while lower expression levels of P3H1 were found in CESC, KICH, LAML, LUAD, LUSC, OV, PRAD, TCGT, and THCA (Figure S2).

### 3.2. Association between P3H1 Expression and Clinical Features

**Figure 3:**
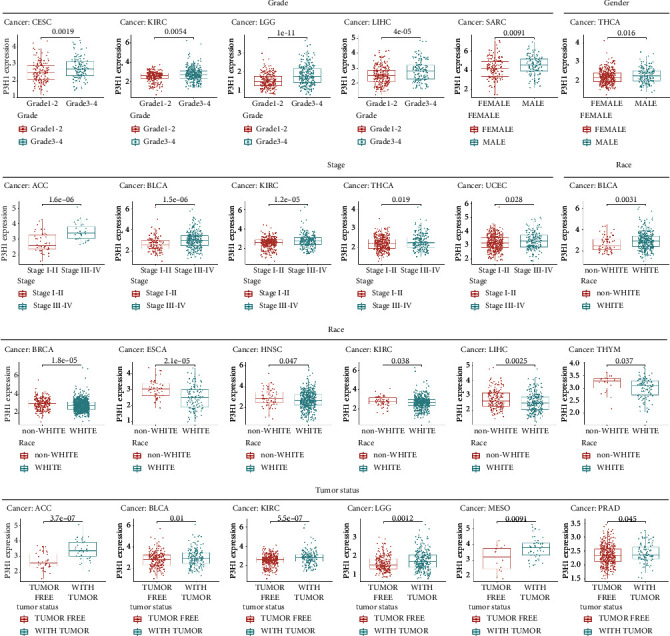
Correlation of P3H1 expression levels with clinical characteristics.

We evaluated the relationship between P3H1 expression and clinical characteristics in human malignancies. In CESC, KIRC, LGG, and LIHC, patients with grades 3–4 expressed significantly higher than patients with grades 1–2 (Figure 3). Furthermore, we observed an increased P3H1 expression in SARC and THCA in male patients (Figure 3). In ACC, BLCA, KIRC, THCA, and UCEC, the expression of P3H1 was considerably elevated in stage III–IV patients compared to stage I–II patients (Figure 3). In terms of race, nonwhite patients showed significantly higher expression in BRCA, ESCA, HNSC, KIRC, LIHC, and THYM than white patients, but lower expression in BLCA (Figure 3). Finally, we also found that P3H1 expression in the presence of tumors was higher in ACC, BLCA, KIRC, LGG, MESO, and PRAD when compared to tumor-free status (Figure 3).

### 3.3. Prognostic Potential of P3H1 in Pan-Cancer

**Figure 4:**
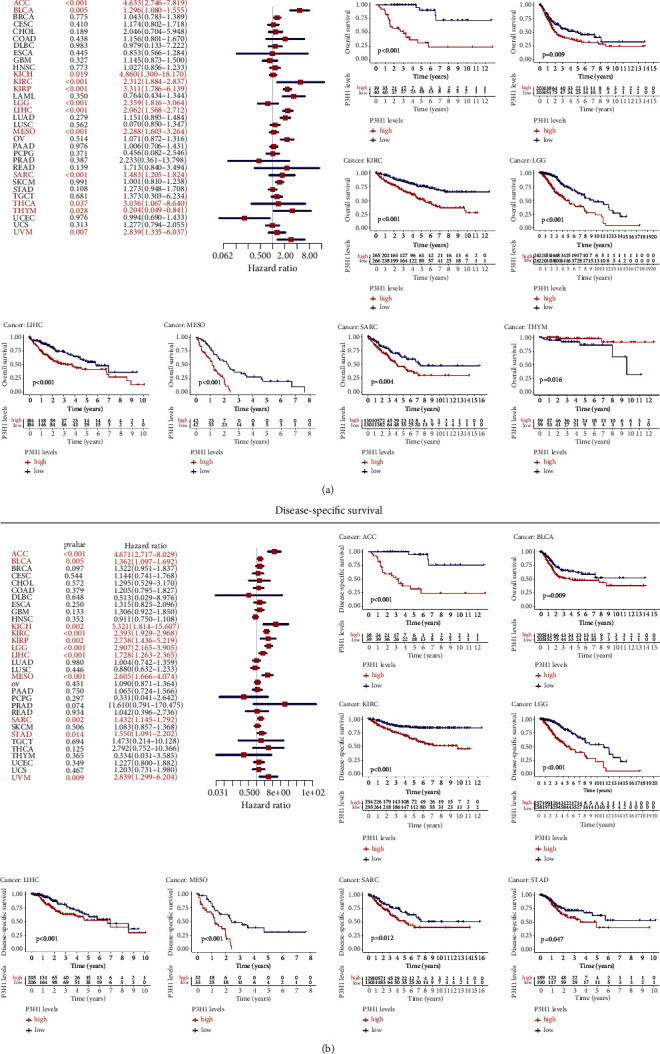
Prognostic potential of P3H1 in pan-cancer. Relationships between P3H1 expression and OS (a) and DSS (b) using univariate Cox regression and Kaplan–Meier survival methods. OS, overall survival; DSS, disease-specific survival.

**Figure 5:**
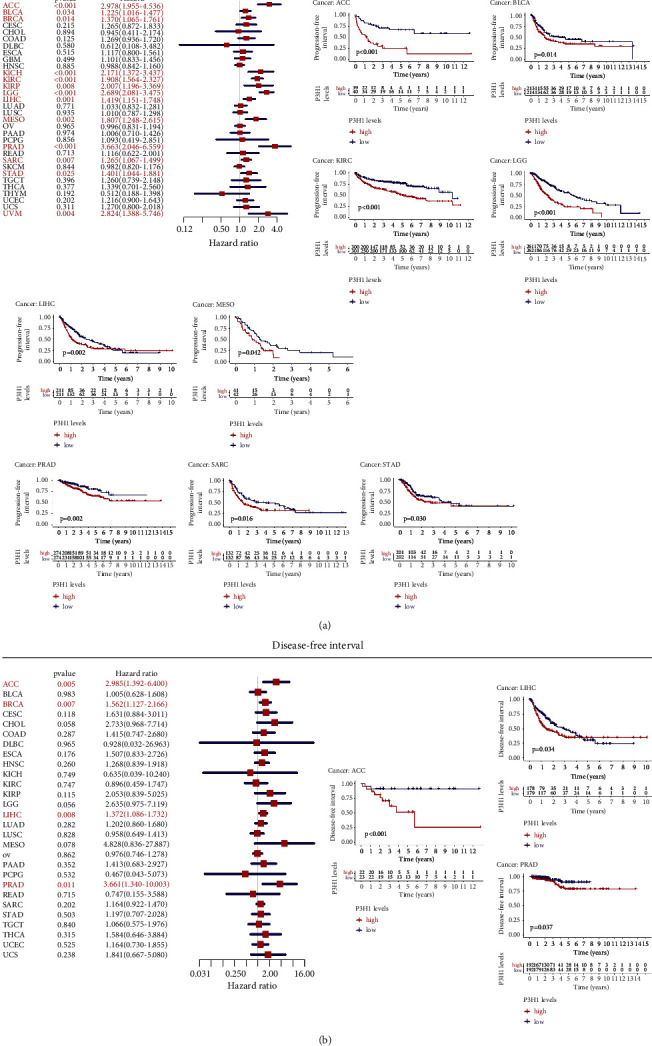
Prognostic potential of P3H1 in pan-cancer. Association between P3H1 expression and DFI (a) and PFI (b) using univariate Cox regression and Kaplan–Meier survival methods. DFI, disease-free interval; PFI, progression-free interval.

The connection between P3H1 expression and survival in malignancies was estimated using TCGA data. Kaplan–Meier analysis showed that the high P3H1 expression was linked to poor survival in ACC (OS, DSS, PFI, and DFI), BLCA (OS, DSS, and PFI), KIRC (OS, DSS, and PFI), LGG (OS, DSS, and PFI), LIHC (OS, DSS, PFI, and DFI), MESO (OS, DSS, and PFI), PRAD (PFI and DFI), SARC (OS, DSS, and PFI), and STAD (PFI and DSS), while P3H1 was a protective prognostic factor in THYM (OS) (Figures 4 and 5).

The Cox regression analysis was further used to assess the P3H1-related survival (OS, DSS, PFI, and DFI) (Figures 4 and 5). As a result, we discovered that P3H1 was a negative prognostic factor in ACC (OS, PFI, DSS, and DFI), BLCA (OS, PFI, and DSS), BRCA (PFI and DFI), KICH (OS, PFI, and DSS), KIRC (OS, PFI, and DSS), KIRP (OS, PFI, and DSS), LGG (OS, PFI, and DSS), LIHC (OS, PFI, DSS, and DFI), MESO (OS, PFI, and DSS), PRAD (PFI and DFI), SARC (OS, PFI, and DSS), STAD (PFI and DSS), THCA (OS), and UVM (OS, PFI, and DSS). However, OS was longer in THYM patients with high P3H1 expression.

### 3.4. Mutation and Methylation Profile of P3H1 in Pan-Cancer

**Figure 6:**
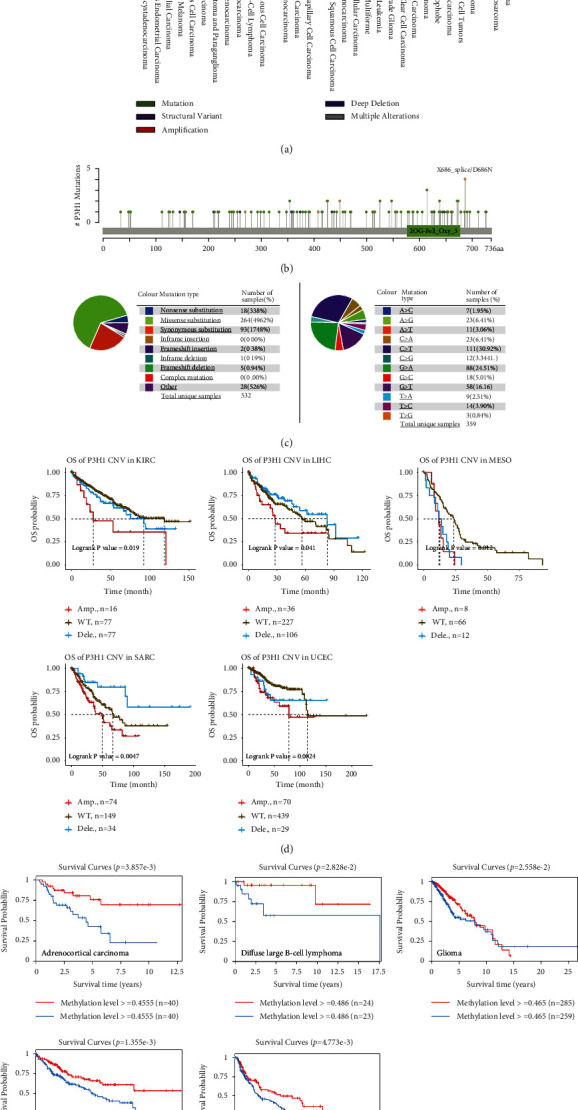
The landscape of P3H1 mutation and methylation in pan-cancer. (a) Mutation level and (b) mutation diagram of P3H1 using the cBioPortal website. (c) The pie chart shows the percentages of the different mutation types of P3H1 using the COSMIC database. The genetic alteration of P3H1 and the prognosis of KIRC, LIHC, MESO, SARC, and UCEC using the Gene Set Cancer Analysis database (d). Correlation of P3H1 methylation of gene body with patient prognosis by the EWAS Data Hub database (e). WT: wild-type; Amp: amplification; Dele: deletion; CNV: copy number variations.

We estimated the alteration frequency of the P3H1 gene in multiple cancers using the cBioPortal website. We found that P3H1 was altered in 223 of the 10,950 patients included in the TCGA (2%) (Figure 6(a)). The highest alteration frequency was associated with amplification and mutation (Figure 6(a)). The types, sites, and case numbers of the P3H1 genetic mutations are further shown in Figure 6(b). A total of 115 mutation sites (including 91 missenses, 13 truncating, 9 splices, and 2 SV/fusion) were found in P3H1 through the cBioPortal database. We further explored the mutation distribution of P3H1 using the COSMIC website. The results showed that missense substitution and synonymous substitution were the main mutation types (Figure 6(c)). The *C* > *T*, *G* > *A*, and *G* > *T* mutations were most common in the P3H1 coding chain (Figure 6(c)). Interestingly, patients with P3H1 amplification had a significantly worse prognosis compared to the P3H1 wild-type in KIRC, LIHC, MESO, SARC, and UCEC (Figure 6(d)).

DNA methylation, an epigenetic modification, can regulate individual growth, development, gene expression patterns, and genome stability without altering the DNA sequence [19, 20]. In recent years, numerous studies have shown that abnormal DNA methylation is closely associated with tumor development and cellular carcinogenesis. Here, we analyzed the impact of P3H1 methylation of the gene body on the prognosis of tumor populations. We revealed that patients with high P3H1 methylation levels presented a better prognosis in ACC, diffuse large B-cell lymphoma, glioma, LIHC, and melanoma (Figure 6(e)).

### 3.5. Validation of P3H1 mRNA and Protein Expression Levels in LIHC

**Figure 7:**
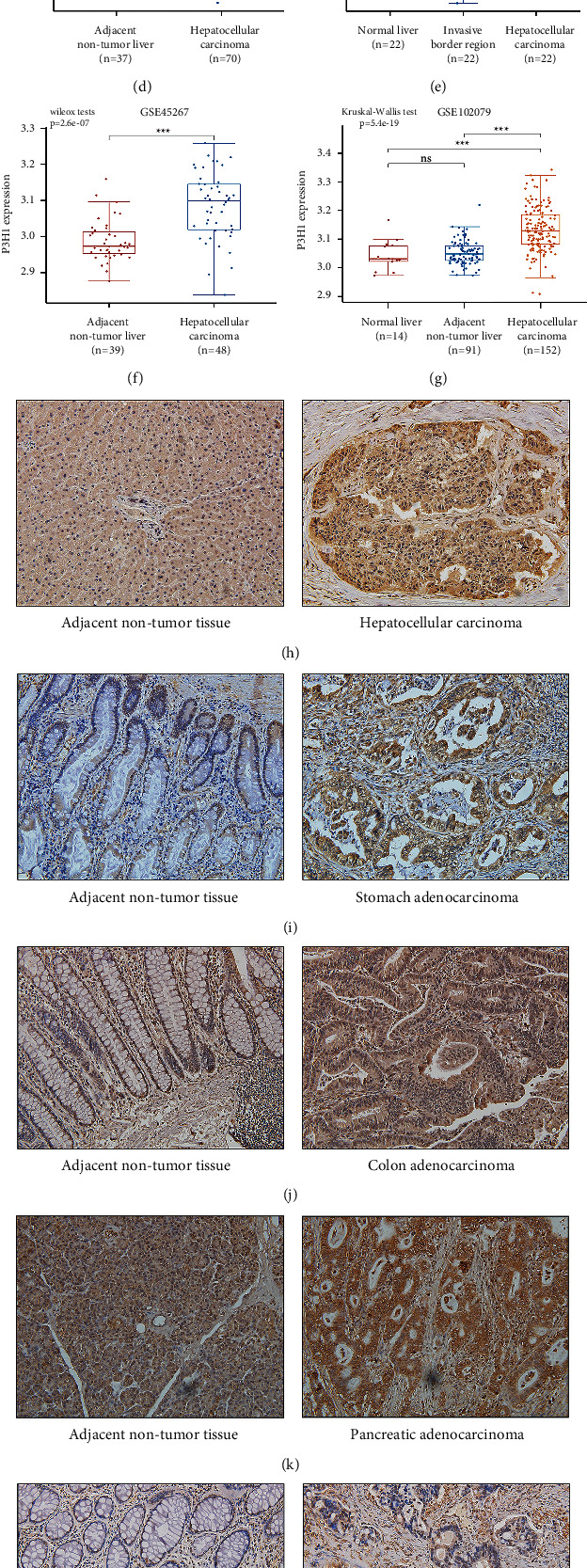
Validation of P3H1 expression in LIHC. Validation of P3H1 expression by the HCCDB database (a). Validation of P3H1 expression by GSE39791 (b), GSE55092 (c), GSE121248 (d), GSE84598 (e), GSE45267 (f), GSE102079 (g), immunohistochemical with 200 × in LIHC (h), STAD (i), COAD (j), PAAD (k), and READ (l). Analysis of AOD values of cancer and adjacent nontumor tissue (m). Protein expression of P3H1 in HCC tissues and nontumor tissues was estimated by western blotting (n). (T) Tumor tissue, (N) adjacent tissue, and AOD: integrated optical density/area (^*∗*^*P* < 0.05, ^*∗∗*^*P* < 0.01, ^*∗∗∗*^*P* < 0.001).

P3H1 in LIHC has been shown to be closely connected to tumor development. Therefore, we further assessed the carcinogenic potential of P3H1 in LIHC. The data extracted from the HCCDB website, GSE39791, GSE55092, GSE121248, GSE84598, GSE45267, and GSE102079 datasets, all demonstrated that P3H1 was highly expressed in LIHC (Figures 7(a)–7(g)). Consistent with the above findings, IHC staining revealed a higher expression level of P3H1 in LIHC than in nontumor tissue (Figures 7(h) and 7(m)). Similar results were also observed in STAD, READ, COAD, and PAAD (Figures 7(i)–7(m)). Fifty patients were separated into two groups based on IHC analysis: low P3H1 expression (*n* = 25) and high P3H1 expression (*n* = 25). The detailed clinicopathological features are shown in Table 2. Chi-squared analysis showed that patients with III-IV stage (*P*=0.037), poor tumor differentiation (*P*=0.047), and larger tumor size (*P*=0.021) exhibited a higher expression of P3H1. Besides, western blotting was performed and the results also showed that P3H1 expression was remarkably higher in tumor tissues (Figure 7(n)).

### 3.6. P3H1 as an Independent Prognostic Risk Factor in LIHC

**Figure 8:**
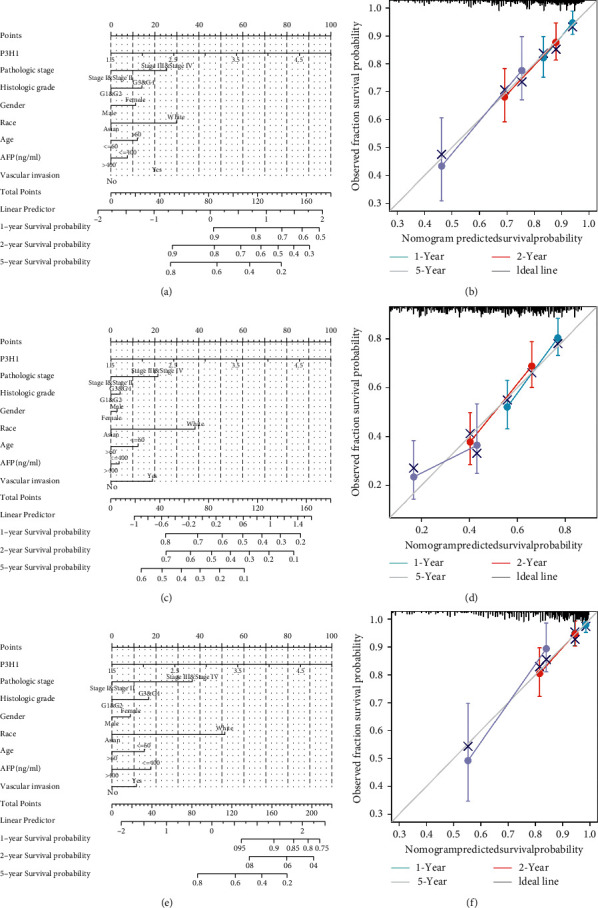
Nomogram establishment and evaluation. Nomogram to predict the 1-year, 2-year, and 5-year OS (a), DSS (c), and PFI (e) of LIHC patients. (e) Calibration curve for nomogram model of OS (b), DSS (d), and PFI (f) in the LIHC-TCGA cohort. OS, overall survival; DSS, disease-specific survival; PFI, progression-free interval.

Using the TCGA dataset, we then investigated whether P3H1 is an independent predictive factor for LIHC patients. As seen in Table S2–S4, the P3H1 expression levels were remarkably correlated with OS, PFI, and DSS in the univariate Cox regression and multivariate Cox regression. To develop a clinically applicable approach for predicting the OS, PFI, and DSS of LIHC patients, we resorted to nomogram predictive models (Figure 8(a), OS : *C*-index = 0.682 (0.647–0.717), *P* < 0.001; Figure 8(c), PFI : *C*-index = 0.660 (0.631–0.688), *P*=0.001; Figure 8(e), DSS : C-index = 0.723 (0.676–0.770), *P*=0.003), considering P3H1 expression levels, TNM stage, histologic grade, gender, race, age, AFP levels, and vascular invasion to predict the probability of the prognosis at 1, 2, and 5 years in the TCGA-LIHC cohort (Figures 8(a), 8(c) and 8(e)). When compared to an ideal model in the entire cohort, the calibration curve confirmed that the nomogram can reliably predict the 1-year, 2-year, and 5-year OS, PFI, and DSS (Figures 8(b), 8(d) and 8(f)).

### 3.7. P3H1 Impacts Cell Proliferation, Migration, and Invasion

**Figure 9:**
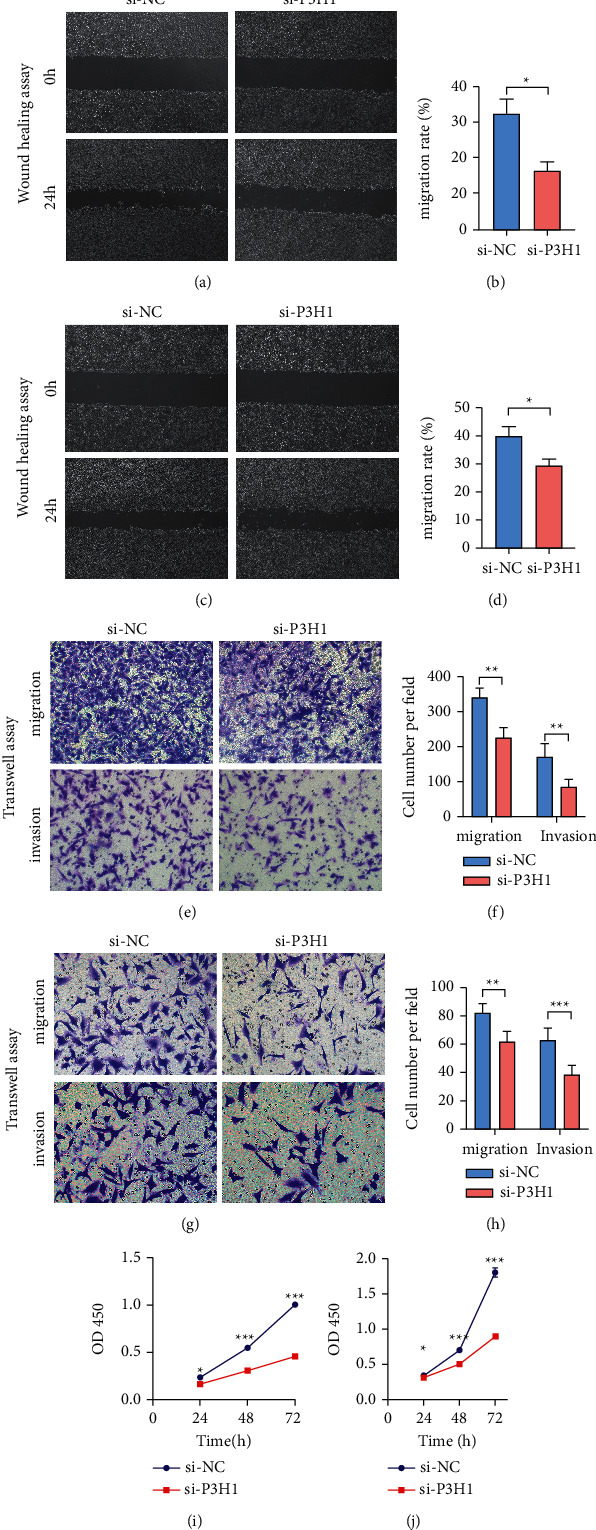
P3H1 impacts cell proliferation, migration, and invasion. Knockout of P3H1 reduced the migration ability of BEL-7402 (a-b) and HLF (c-d) cells by a wound-healing assay. Migration and invasion assays of BEL-7402 (e-f) and HLF (g-h) cells after the knockdown of P3H1. The CCK-8 analysis showed that the proliferative ability was inhibited in BEL-7402 and HLF cells (IJ) (^*∗*^*P* < 0.05, ^*∗∗*^*P* < 0.01, ^*∗∗∗*^*P* < 0.001).

We further confirmed the role of P3H1 in liver cancer cells. We first constructed P3H1 knocked-down BEL-7402 and HLF cells using the lentivirus technology (Figures S3A, S3B). Compared to controls, the wound-healing assay revealed that the percentage of cell scratch areas healed was significantly reduced following knockdown of P3H1 in BEL-7402 and HLF cells (Figures 9(a)–9(d)). The transwell migration and invasion assays showed that by knocking down P3H1 in the BEL-7402 and HLF cells, the number of cells crossing the chamber membrane from the upper to lower chambers was significantly reduced (Figures 9(e)–9(h)). The CCK8 assay demonstrated that the proliferative ability of cells at 24, 48, and 72 h was significantly lower after knockdown of P3H1 in the BEL-7402 and HLF cells (Figures 9(i) and 9(j)). The experiments revealed that P3H1 can be involved in liver cancer progression.

### 3.8. P3H1-Related Gene Enrichment Analysis

**Figure 10:**
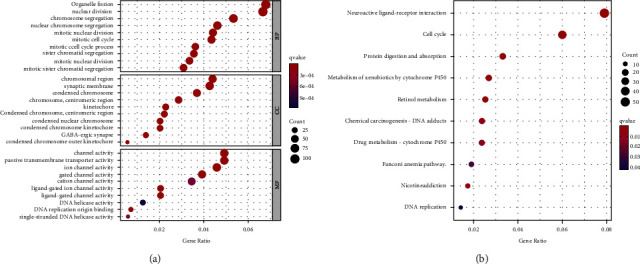
The GO enrichment analysis (a) and KEGG enrichment analysis (b).

Differences in gene expression between patients with P3H1 high expression and those with P3H1 low expression are shown in Figure S4 and Table S5. We further performed GO and KEGG enrichment analysis using P3H1 as well as the above differential genes to explore the biological function of P3H1 in LIHC. We showed that the most prevalent biological processes were “organelle fission” and “nuclear division” (Figure 10(a)). GO cellular compartment analysis revealed that P3H1 and related genes were highly enriched in the “chromosomal region” and “synaptic membrane” (Figure 10(a)). The most significantly enriched GO term in the molecular function was “channel activity,” “passive transmembrane transporter activity,” and “ion channel activity” (Figure 10(a)). The KEGG data in Figure 10(b) showed that “neuroactive ligand−receptor interaction” and “cell cycle” might be involved in the effect of P3H1 on tumor pathogenesis.

### 3.9. The Relationship between Immune Cell Infiltration, Immune Checkpoint Inhibitor Treatment, and P3H1 in LIHC

**Figure 11:**
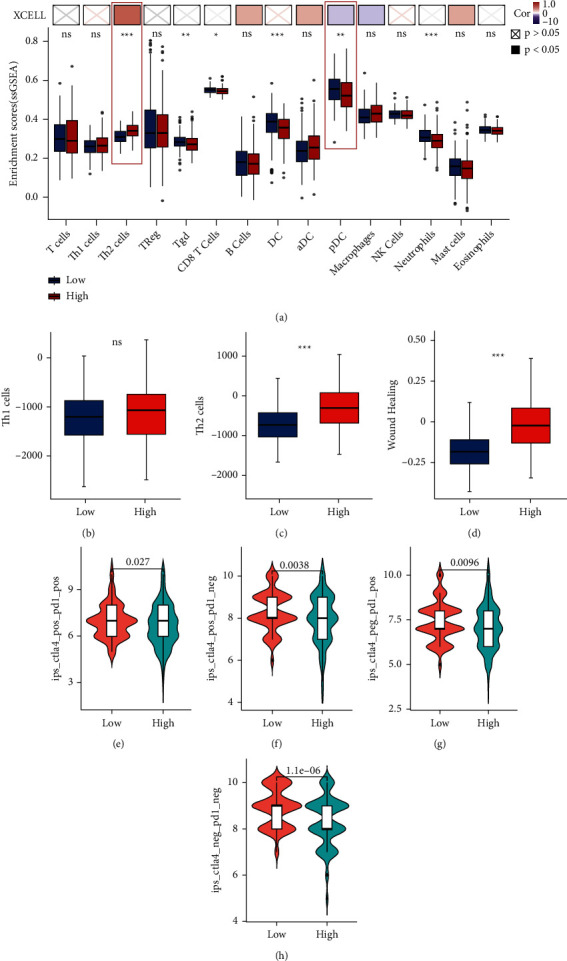
The relationship among immune cell infiltration, immune checkpoint inhibitor treatment, and P3H1 in LIHC. Immunoinfiltration analysis according to XCELL and ssGSEA algorithms (a). Analysis of P3H1 expression concerning Th1, Th2, and immune subtype C1 (wound healing) using the CAMOIP website (b–d). Differential analysis of ICI treatment efficacy in high and low P3H1 expression groups (e–h) (ns P > 0.05, ^*∗*^*P* < 0.05, ^*∗∗*^*P* < 0.01, ^*∗∗∗*^*P* < 0.001).

After finding that P3H1 could affect the malignant behavior of tumor cells, we further explored whether P3H1 was correlated with immune cell infiltration. We used the XCELL and ssGSEA algorithms, respectively, and took the intersection to further confirm the reliability of the results. As shown in Figure 11(a), we found that P3H1 expression was positively correlated with Th2 cell infiltration. In contrast, the plasmacytoid DC (pDC) cell infiltration was negatively correlated with the P3H1 expression. Next, the Immune Scores module on the CAMOIP website was used to reverify the above findings. The results were consistent with the above conclusion (Figures 11(b) and 11(c)).

GATA3 has been identified as a master regulator that controls Th2 cell differentiation and the production of Th2 cytokines by binding to a broad range of Th2 cytokine gene locus [21], whereas GATA3 suppresses Th1 cell differentiation by blocking transcription of Th1-specific genes [22, 23]. GATA3 also serves as a commonly used Th2 cell marker. We found a significant positive correlation between the expression levels of P3H1 and GATA3 using the GEPIA2 website analysis (Figure S5A). We then revealed that P3H1 was also significantly positively related to IL10 (Th2 cytokine) [24]. These results further confirm our findings above (Figure S5B). Interestingly, the above analysis also showed that LIHC patients with high P3H1 expression had higher C1 (wound healing) scores (Figure 11(d)). As far as we know, C1 (wound healing) had elevated expression of angiogenic genes, a high proliferation rate, and a Th2 cell bias to the adaptive immune infiltration [17]. This reaffirmed the above conclusion that P3H1 was positively correlated with Th2 cell infiltration.

ICI therapy is at the forefront of treatment for liver cancer. Here, we also found that the combination of anti-PD-1 and CTLA4, anti-PD-1 monotherapy, anti-CTLA-4 monotherapy, and other ICI therapies presented poorer outcomes in LIHC patients with high P3H1 expression compared to those with low P3H1 expression (Figures 11(e)–11(h)).

### 3.10. Exploration of Upstream lncRNAs and miRNAs of P3H1 in LIHC

**Figure 12:**
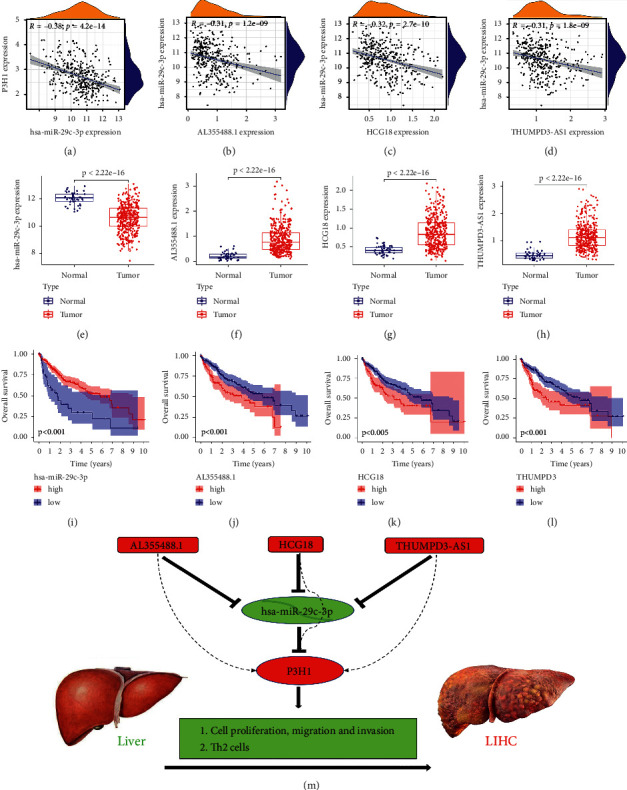
Exploration of upstream lncRNAs and miRNAs of P3H1 in LIHC. hsa-miR-29c-3p and P3H1 correlation analysis (a), and its expression (b) and prognosis analysis (c). AL355488.1 and hsa-miR-29c-3p correlation analysis (d), and its expression (e) and prognosis analysis (f). HCG18 and hsa-miR-29c-3p correlation analysis (g), and its expression (h) and prognosis analysis (i). THUMPD3-AS1 and hsa-miR-29c-3p correlation analysis (j), and its expression (k) and prognosis analysis (l). The model of P3H1 in the carcinogenesis of liver cancer (m).

Noncoding RNAs (ncRNAs) play a crucial regulatory role in gene expression. We used the starBase database to predict the upstream miRNAs of P3H1, and identified 12 significantly associated miRNAs (filtered with programNum ≥2, Table S6). The miRNAs should have a negative connection with P3H1 based on their action mechanism in modifying target genes. Thus, we further analyzed the correlation between these miRNAs and P3H1 in the TCGA-LIHC data (filtered with the value of *R* ≤ −0.3), and the results revealed that only hsa-miR-29c-3p (*R* = −0.38) met the above filtering criteria (Figures 12(a) and S6A). As shown in Figures 12(b) and 12(c), hsa-miR-29c-3p was expressed low in LIHC compared to paraneoplastic tissues, and patients with low hsa-miR-29c-3p expression had a poor prognosis. These findings indicated that hsa-miR-29c-3p was the most plausible upstream miRNA for P3H1.

Next, the upstream lncRNAs of hsa-miR-29c-3p were estimated via the starBase website, and a total of 132 lncRNAs were discovered (Table S7). The lncRNAs can enhance mRNA expression by binding to shared miRNAs competitively according to the competitive endogenous RNA (ceRNA) theory. As shown in Figures 12(d)–12(l), we revealed that only the correlation coefficient among AL355488.1 (*R* = −0.31), HCG18 (*R* = −0.32), THUMPD3-AS1 (*R* = −0.31), and hsa-miR-29c-3p was less than −0.3, and all these lncRNAs were highly expressed and were associated with poor prognosis in LIHC. In addition, P3H1 expression was also found to be positively associated with AL355488.1 (*R* = 0.48), HCG18 (*R* = 0.37), and THUMPD3-AS1 (*R* = 0.39) (Figures S6B–S6D). Thus, AL355488.1, HCG18, and THUMPD3-AS1 were the most likely upstream lncRNAs of the hsa-miR-29c-3p-P3H1 axis in LIHC.

## 4. Discussion

Although P3H1 plays an extremely important role in human collagen synthesis, research on the role of P3H1 in disease is still limited, particularly in tumors. In this study, we performed a systematic analysis of P3H1 in pan-cancer. We first compared the P3H1 expression in tumors and paracancerous tissues. We found that P3H1 was significantly overexpressed in most tumor types. The immunohistochemistry assay further demonstrated that P3H1 expression was significantly higher in LIHC, STAD, COAD, READ, and PAAD. These findings imply that P3H1 might exert a key role in the progression of tumors.

We also discovered that increased P3H1 expression was linked to the poor OS, DSS, DFI, and PFI, especially in LIHC. Furthermore, patients with P3H1 amplification had a significantly worse prognosis compared to P3H1 wild-type in KIRC, LIHC, MESO, SARC, and UCEC. The P3H1 hypermethylation levels were associated with a good prognosis in several tumors, especially in LIHC. Therefore, we further explored the role of P3H1 in LIHC and found that its expression was significantly elevated with increasing grades. P3H1 expression levels could be employed as an independent prognostic indicator for LIHC patients. In addition, the ability of proliferation, migration, and invasion was significantly downregulated in liver cancer cells with P3H1 knockdown.

It is well known that ncRNAs are involved in the regulation of gene expression via the ceRNA mechanism [25–27]. Previous studies also revealed that, compared to paraneoplastic tissues, has-mir-29c-3p was lowly expressed in CRC tissues, and has-mir-29c-3p overexpression inhibits cell proliferation and migration [28]. Wang et al. also found lower has-mir-29c-3p expression in HBV-infected patients with hyperfibrosis compared to patients with fibrosis stage 0, indicating that has-mir-29c-3p might be involved in the fibrosis process [29]. Besides, HCG18 and THUMPD3-AS1 have been reported to function as oncogenes in some cancers, including liver cancer. For example, Zhou et al. found that HCG18 was highly expressed in liver cancer tissues and that HCG18 silencing could inhibit the proliferation and migration while inducing the apoptosis of liver cancer cells [30]. In this study, we confirmed that AL355488.1, HCG18, and THUMPD3-AS1 may be the most promising upstream lncRNAs of the hsa-miR-29c-3p-P3H1 axis in LIHC. Thus, these ncRNAs may contribute to the development of LIHC by regulating the expression of P3H1.

Immune infiltrates in the tumor microenvironment have been shown to exert a crucial role in tumor development and will influence the clinical outcome of cancer patients [31]. We found that there was a positive connection between P3H1 expression and Th2 cell infiltration levels by various immune infiltration algorithms and immune subtype analysis. Interestingly, there is no evidence that Th2 immunity promotes cancer origin, progression, and metastasis. For example, several studies have shown that Th2 cells are linked to the progression of breast and cervical tumors [32, 33]. Furthermore, Th2 cells have been proven to promote metastasis in breast, colorectal, and lung cancer [34–37]. Thus, this is further evidence that P3H1 is closely linked to Th2 and may be a factor in its poor prognosis. It is well known that immune cell infiltration plays an important role in ICI therapy. For example, immunosuppressive microenvironments rich in Th2 cells are not conducive to the efficacy of ICI. Collectively, these findings indicated that ICI treatment was less effective in LIHC patients with high P3H1 expression.

It is worth noting that although we have provided a comprehensive analysis of the role of P3H1 in pan-cancer, there are still some restrictions. Many of the findings from our current study have not been reported because they are based on bioinformatics analysis. We have only confirmed P3H1 expression in LIHC, STAD, COAD, READ, and PAAD at the tissue level, as well as the role of P3H1 in LIHC at the cellular level, whereas the role of P3H1 in other cancers will require more research. In addition, the AL355488.1, HCG18, and THUMPD3-AS1/hsa-miR-29c-3p-P3H1 axis also require further experimental validation.

## 5. Conclusion

Our research discovered that P3H1 was substantially upregulated in almost all tumors, and a high expression level of P3H1 was linked to poor prognosis and Th2 cell infiltration in LIHC. We also demonstrated that ICI treatment was less effective in LIHC patients with high P3H1 expression. Furthermore, the expression of P3H1 was found to be an independent prognostic factor for LIHC. Knockdown of P3H1 significantly inhibited proliferation, migration, and invasive activity in liver cancer cells. Finally, we also identified an upstream regulatory mechanism of P3H1 in LIHC, namely, the AL355488.1, HCG18, and THUMPD3-AS1/hsa-miR-29c-3p-P3H1 axis (Figure 12(m)). Therefore, interference with P3H1 may be a therapeutic target for patients with LIHC. However, more basic experiments and large clinical trials need to be conducted in the future to corroborate these findings.

## Figures and Tables

**Table 1:** Full names of the 33 cancers in the TCGA data.

Abbreviation	Full name
ACC	Adrenocortical carcinoma
BLCA	Bladder urothelial carcinoma
BRCA	Breast invasive carcinoma
CESC	Cervical squamous cell carcinoma and endocervical adenocarcinoma
CHOL	Cholangiocarcinoma
COAD	Colon adenocarcinoma
DLBC	Lymphoid neoplasm diffuse large B-cell lymphoma
ESCA	Esophageal carcinoma
GBM	Glioblastoma multiforme
HNSC	Head and neck squamous cell carcinoma
KICH	Kidney chromophobe
KIRC	Kidney renal clear cell carcinoma
KIRP	Kidney renal papillary cell carcinoma
LAML	Acute myeloid leukemia
LGG	Brain lower grade glioma
LIHC	Liver hepatocellular carcinoma
LUAD	Lung adenocarcinoma
LUSC	Lung squamous cell carcinoma
MESO	Mesothelioma
OV	Ovarian serous cystadenocarcinoma
PAAD	Pancreatic adenocarcinoma
PCPG	Pheochromocytoma and paraganglioma
PRAD	Prostate adenocarcinoma
READ	Rectum adenocarcinoma
SARC	Sarcoma
SKCM	Skin cutaneous melanoma
STAD	Stomach adenocarcinoma
TGCT	Testicular germ cell tumors
THCA	Thyroid carcinoma
THYM	Thymoma
UCEC	Uterine corpus endometrial carcinoma
UCS	Uterine carcinosarcoma
UVM	Uveal melanoma

**Table 2:** The relationship between P3H1 expression and clinicopathological features in LIHC.

Clinical variables	No. of patients	P3H1 expression level		*P*-value
	*n* = 50	Low (*n* = 25)	High (*n* = 25)	
Gender				
Female	18	11	7	0.239
Male	32	14	18	

Age (years)				
<60	31	17	14	0.382
≥60	19	8	11	

HBsAg				
Negative	12	7	5	0.508
Positive	38	18	20	

AFP (ng/ml)				
<400	21	12	9	0.390
≥400	29	13	16	

Liver cirrhosis				
No	16	10	6	0.225
Yes	34	15	19	

Child–Pugh class				
A	34	19	15	0.225
B	16	6	10	

Tumor size (cm)				
<5 cm	20	14	6	0.021
≥5 cm	30	11	19	

Vascular invasion				
No	32	19	13	0.077
Yes	18	6	12	

Tumor differentiation				
Well	27	17	10	0.047
Poor	23	8	15	

TNM stage				
I–II	33	20	13	0.037
III–IV	17	5	12	

## Data Availability

The dataset analyzed during the current study consists of public datasets such as TCGA, GEO, and information from our hospital patients, which can be downloaded from the methodology section of the manuscript or obtained from the corresponding author upon reasonable request.

## Supplementary Materials

Supplementary Materials Table S1. Database and its website. Table S2. TCGA-LIHC data were used to conduct univariate and multivariate analyses of variable factors affecting overall survival. Table S3. TCGA-LIHC data were used to conduct univariate and multivariate analyses of variable factors affecting the progress-free interval. Table S4. TCGA-LIHC data were used to conduct univariate and multivariate analyses of variable factors affecting disease-specific survival. Table S5. Differences in gene expression between patients with high P3H1 expression and those with low P3H1 expression. Table S6. Coexpression miRNA of P3H1 obtained using starBase. Table S7. Coexpression lncRNA of hsa-miR-29c-3p obtained using starBase. Figure S1. mRNA expression of the P3H1 gene. (A) P3H1 expression in men and women of normal tissue using GTEx data. (B) The expression of P3H1 in 38 kinds of cancer cell lines from the CCLE database (^*∗*^*P* < 0.05, ^*∗∗∗*^*P* < 0.001). Figure S2. P3H1 expression in cancer and normal tissues using the TCGA and GTEx dataset (^*∗*^*P* < 0.05, ^*∗∗∗*^*P* < 0.001). Figure S3. The western blot method was utilized to assess the P3H1 level after transfection with lentivirus (^*∗∗∗*^*P* < 0.001). Figure S4. Heatmap for variance analysis between patients with high P3H1 expression and those with low P3H1 expression. Figure S5. P3H1 and GATA3 (A) and IL10 (B) correlation analysis in tumor tissues of TCGA-LIHC cohort. Figure S6. Exploration of upstream lncRNAs and miRNAs of P3H1 in LIHC. (A) The binding sequence of hsa-miR-29c-3p on P3H1 using the starBase database. (B) AL355488.1 and P3H1 correlation analysis. (C) HCG18 and P3H1 correlation analysis. (D) THUMPD3-AS1 and P3H1 correlation analysis.
