# Supplementary material for: The Prognostic Significance and Potential Mechanism of Prolyl 3-Hydroxylase 1 in Hepatocellular Carcinoma
Source: J Oncol. 2022 Oct 26;2022:7854297. doi: 10.1155/2022/7854297 (PMC9629929; doi:10.1155/2022/7854297)
Supplement: Supplementary Materials — Table S1. Database and its website. Table S2. TCGA-LIHC data were used to conduct univariate and multivariate analyses of variable factors affecting overall survival. Table S3. TCGA-LIHC data were used to conduct univariate and multivariate analyses of variable factors affecting the progress-free interval. Table S4. TCGA-LIHC data were used to conduct univariate and multivariate analyses of variable factors affecting disease-specific survival. Table S5. Differences in gene expression between patients with high P3H1 expression and those with low P3H1 expression. Table S6. Coexpression miRNA of P3H1 obtained using starBase. Table S7. Coexpression lncRNA of hsa-miR-29c-3p obtained using starBase. Figure S1. mRNA expression of the P3H1 gene. (A) P3H1 expression in men and women of normal tissue using GTEx data. (B) The expression of P3H1 in 38 kinds of cancer cell lines from the CCLE database (∗P < 0.05, ∗∗∗P < 0.001). Figure S2. P3H1 expression in cancer and normal tissues using the TCGA and GTEx dataset (∗P < 0.05, ∗∗∗P < 0.001). Figure S3. The western blot method was utilized to assess the P3H1 level after transfection with lentivirus (∗∗∗P < 0.001). Figure S4. Heatmap for variance analysis between patients with high P3H1 expression and those with low P3H1 expression. Figure S5. P3H1 and GATA3 (A) and IL10 (B) correlation analysis in tumor tissues of TCGA-LIHC cohort. Figure S6. Exploration of upstream lncRNAs and miRNAs of P3H1 in LIHC. (A) The binding sequence of hsa-miR-29c-3p on P3H1 using the starBase database. (B) AL355488.1 and P3H1 correlation analysis. (C) HCG18 and P3H1 correlation analysis. (D) THUMPD3-AS1 and P3H1 correlation analysis. [file 7854297.f1.pdf]

Figure S1 mRNA expression of the P3H1 gene. (A) P3H1 expression in men and women of normal tissue using GTEx data. (B) The expression of P3H1 in 38 kinds of cancer cell lines from the CCLE database. ($^{*}P < 0.05$, $^{***}P < 0.001$)

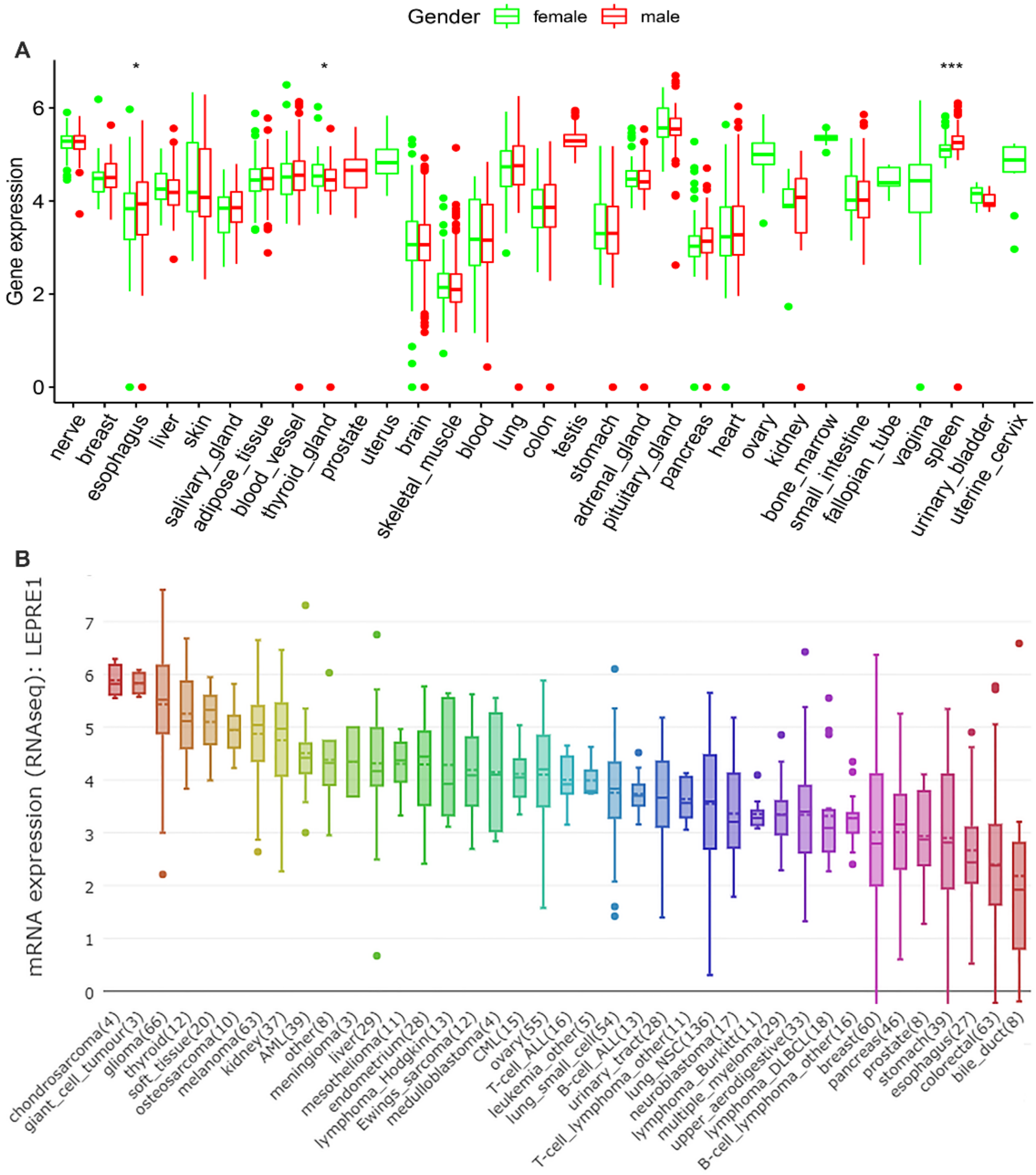

Figure S2 P3H1 expression in cancer and normal tissues using the TCGA and GTEx dataset. ($^{*}P<0.05$, $^{***}P<0.001$)

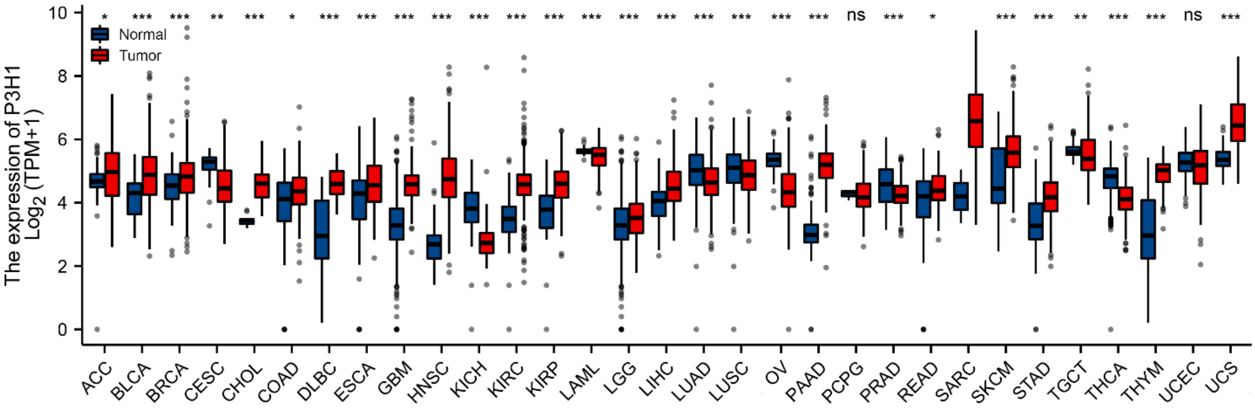

Figure S3 The western blot method was utilized to assess the P3H1 level after transfection with lentivirus. ($^{***}P<0.001$)

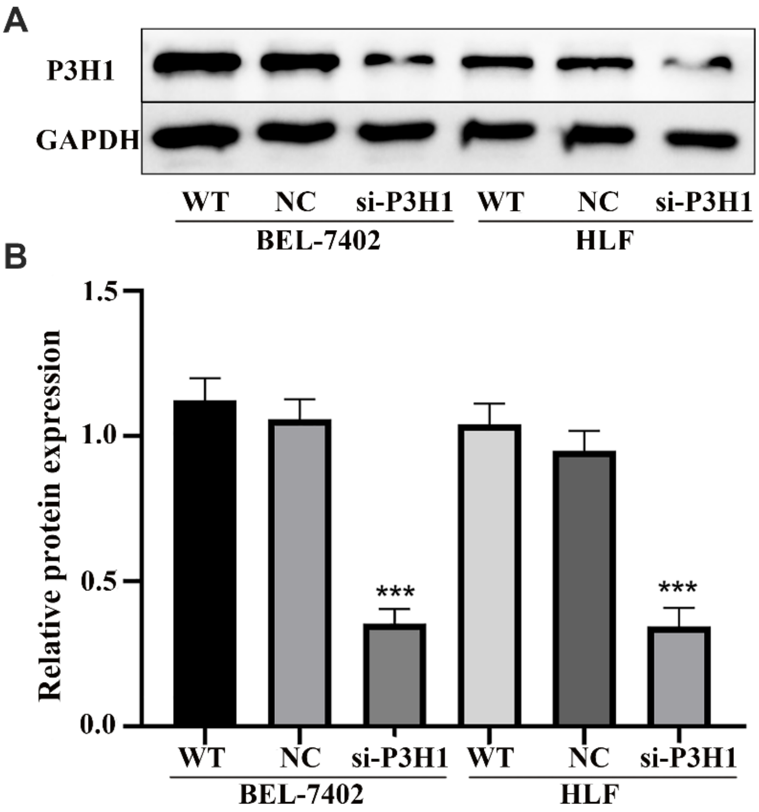

Figure S4 Heatmap for variance analysis between patients with high P3H1 expression and those with low P3H1 expression.

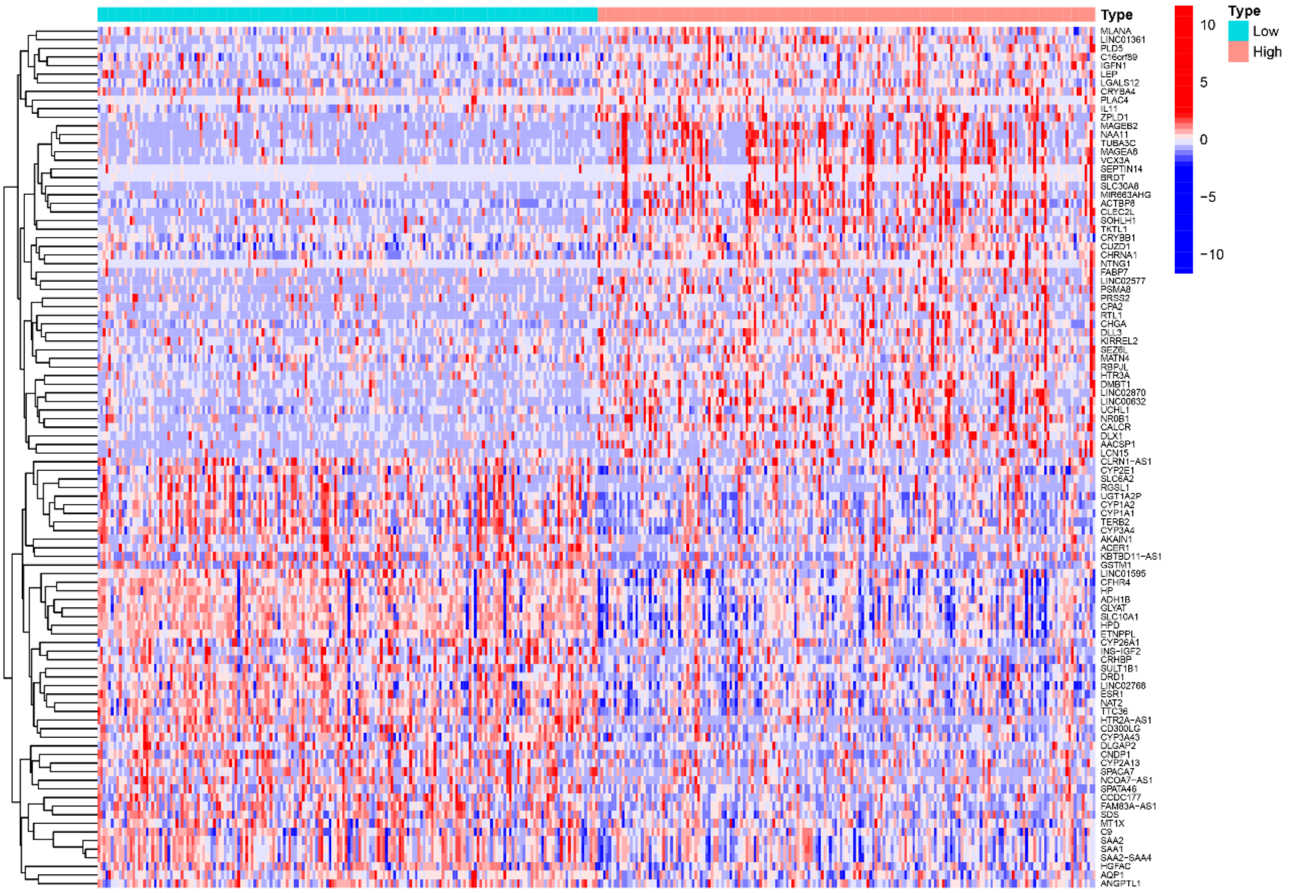

Figure S5 P3H1 and GATA3 (A) and IL10 (B) correlation analysis in tumor tissues of TCGA-LIHC cohort.

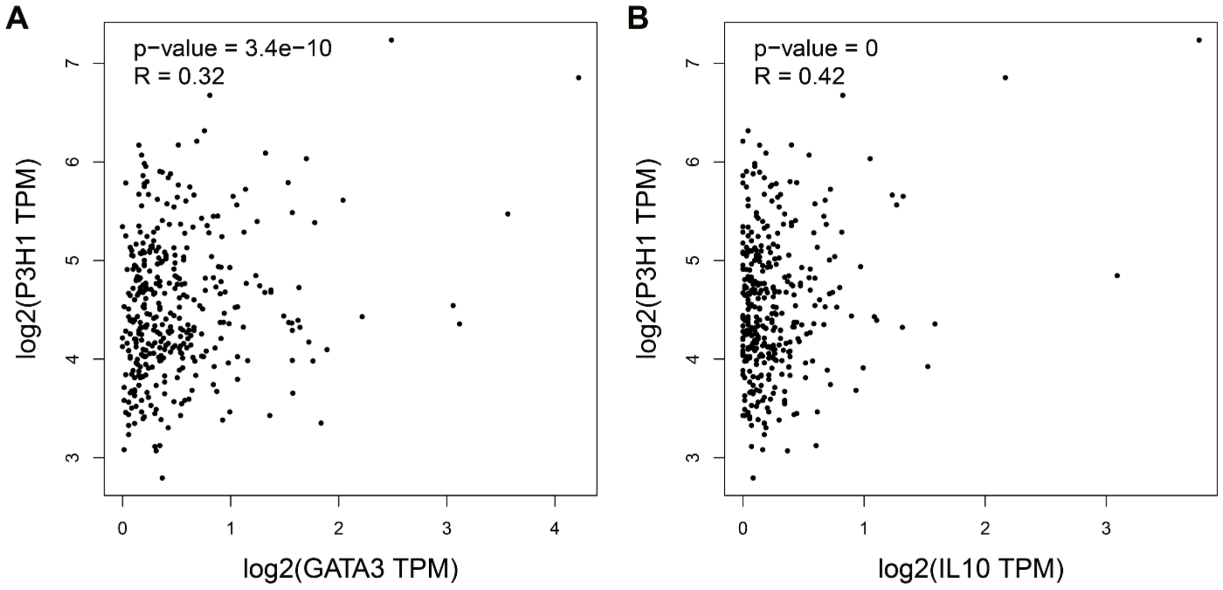

Figure S6 Exploration of upstream lncRNAs and miRNAs of P3H1 in LIHC. (A) The binding sequence of hsa-miR-29c-3p on P3H1 using starBase database. (B) AL355488.1 and P3H1 correlation analysis. (C) HCG18 and P3H1 correlation analysis. (D) THUMPD3-AS1 and P3H1 correlation analysis.

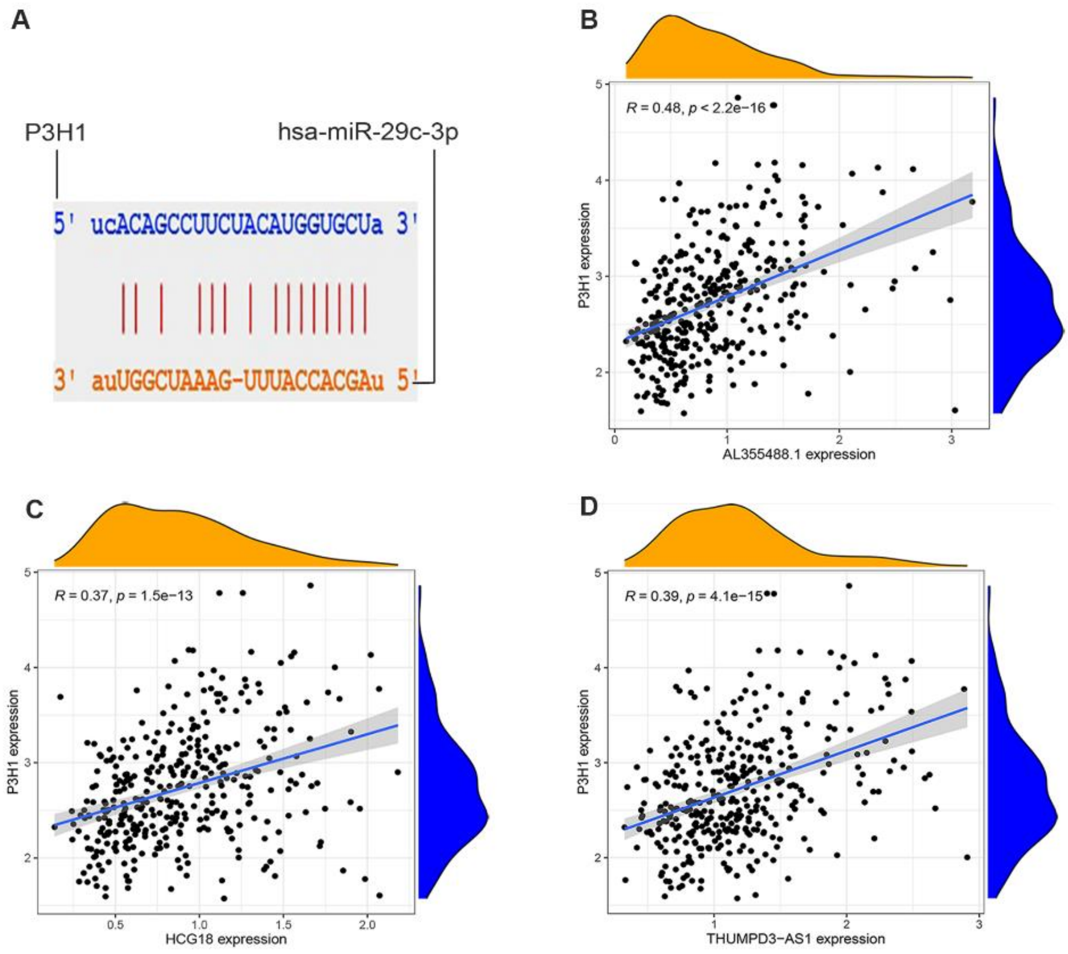

Table S1 Database and its website.

Databases	Website
Xena Browser	https://xenabrowser.net/datapages/
The Cancer Cell Line Encyclopedia (CCLE)	https://portals.broadinstitute.org/ccle/about
cBioPortal	http://www.cbioportal.org/
Catalogue of Somatic Mutations in Cancer (COSMIC)	https://cancer.sanger.ac.uk/cosmic/
Gene Expression Omnibus (GEO)	https://www.ncbi.nlm.nih.gov/geo/
TIMER2.0	http://timer.cistrome.org/
The Cancer Genome Atlas (TCGA) Program	https://www.cancer.gov/about-nci/organization/ccg/research/structural-genomics/tcga
International Cancer Genome Consortium (ICGC) Data Portal	https://dcc.icgc.org/
Gene Set Cancer Analysis (GSCA)	http://bioinfo.life.hust.edu.cn/GSCA/#/
EWAS Data Hub	https://ngdc.cncb.ac.cn/ewas/datahub/index
Clinical Proteomic Tumor Analysis Consortium (CPTAC) Confirmatory/Discovery dataset	http://ualcan.path.uab.edu/analysis-prot.html
starBase	
Integrative Molecular Database of Hepatocellular	http://starbase.sysu.edu.cn/
Carcinoma (HCCDB)	http://lifeome.net/database/hccdb/home.html
Comprehensive Analysis on Multi-Omics of Immunotherapy in Pan-cancer (CAMOIP)	https://www.camoip.net/
The Cancer Immunome Database (TCIA)	https://tcia.at/home

Table S2 TCGA-LIHC data was used to conduct univariate and multivariate analyses of variable factors affecting overall survival

**Characteristics**	**Total(N)**	**HR(95% CI) Univariate analysis**	**P value Univariate analysis**	**HR(95% CI) Multivariate analysis**	**P value Multivariate analysis**
P3H1	373				
Low	186	Reference			
High	187	2.054 (1.438-2.933)	<0.001	1.892 (1.286-2.782)	0.001
Age	373				
<=60	177	Reference			
>60	196	1.205 (0.850-1.708)	0.295		
Gender	373				
Female	121	Reference			
Male	252	0.793 (0.557-1.130)	0.200		
Race	344				
White	185	Reference			
Asian	159	0.755 (0.519-1.100)	0.144		
BMI	336				
<=25	177	Reference			
>25	159	0.798 (0.550-1.158)	0.235		
Pathologic stage	349				
Stage I&Stage II	259	Reference			
Stage III&Stage IV	90	2.504 (1.727-3.631)	<0.001	2.244 (1.539-3.270)	<0.001
Histologic grade	368				
G1&G2	233	Reference			
G3&G4	135	1.091 (0.761-1.564)	0.636		
Vascular invasion	317				
No	208	Reference			
Yes	109	1.344 (0.887-2.035)	0.163		
AFP(ng/ml)	279				
<=400	215	Reference			
>400	64	1.075 (0.658-1.759)	0.772		

Table S3 TCGA-LIHC data was used to conduct univariate and multivariate analyses of variable factors affecting the progress-free interval

Characteristics	Total(N)	HR(95% CI) Univariate analysis	P value Univariate analysis	HR(95% CI) Multivariate analysis	P value Multivariate analysis
P3H1	373				
Low	186	Reference			
High	187	1.607 (1.200-2.152)	0.001	1.478 (1.045-2.089)	0.027
Age	373				
<=60	177	Reference			
>60	196	0.960 (0.718-1.284)	0.783		
Gender	373				
Female	121	Reference			
Male	252	0.982 (0.721-1.338)	0.909		
Race	344				
White	185	Reference			
Asian	159	0.781 (0.577-1.057)	0.109		
BMI	336				
<=25	177	Reference			
>25	159	0.936 (0.689-1.272)	0.673		
Pathologic stage	349				
Stage I&Stage II	259	Reference			
Stage III&Stage IV	90	2.201 (1.591-3.046)	<0.001	1.763 (1.172-2.652)	0.007
Histologic grade	368				
G1&G2	233	Reference			
G3&G4	135	1.152 (0.853-1.557)	0.355		
Vascular invasion	317				
No	208	Reference			
Yes	109	1.676 (1.196-2.348)	0.003	1.366 (0.948-1.969)	0.094
AFP(ng/ml)	279				
<=400	215	Reference			
>400	64	1.045 (0.698-1.563)	0.832		

Table S4 TCGA-LIHC data was used to conduct univariate and multivariate analyses of variable factors affecting disease-specific survival

**Characteristics**	**Total(N)**	**HR(95% CI) Univariate analysis**	**P value Univariate analysis**	**HR(95% CI) Multivariate analysis**	**P value Multivariate analysis**
P3H1	365				
Low	182	Reference			
High	183	2.187 (1.383-3.460)	<0.001	1.925 (1.138-3.256)	0.015
Age	365				
<=60	174	Reference			
>60	191	0.846 (0.543-1.317)	0.458		
Gender	365				
Female	118	Reference			
Male	247	0.813 (0.516-1.281)	0.373		
Race	336				
White	180	Reference			
Asian	156	0.648 (0.399-1.051)	0.079	0.977 (0.571-1.670)	0.931
BMI	329				
<=25	175	Reference			
>25	154	0.826 (0.512-1.330)	0.431		
Pathologic stage	341				
Stage I&Stage II	254	Reference			
Stage III&Stage IV	87	3.803 (2.342-6.176)	<0.001	3.222 (1.916-5.418)	<0.001
Histologic grade	360				
G1&G2	227	Reference			
G3&G4	133	1.086 (0.683-1.728)	0.726		
Vascular invasion	309				
No	204	Reference			
Yes	105	1.277 (0.707-2.306)	0.418		
AFP(ng/ml)	275				
<=400	214	Reference			
>400	61	0.867 (0.450-1.668)	0.668		

Table S5 Differences in gene expression between patients with high P3H1 expression and those with low P3H1 expression

gene	lowMean	highMean	logFC	pValue	fdr
PIN1-DT	0.022946	0.046482	1.018457	0.001355	0.002596
KCNQ4	0.121209	0.367381	1.599776	2.72E-07	1.17E-06
LDHAL6A	0.0164	0.032947	1.006468	0.001002	0.001966
TINAG	1.597018	3.344457	1.066391	7.00E-05	0.000177
HOXA9	0.026945	0.090596	1.749452	0.002007	0.0037
SLC38A5	0.275823	0.927919	1.750255	4.56E-06	1.50E-05
RCOR2	0.148879	0.668262	2.166276	2.73E-10	2.25E-09
NCCRP1	0.073937	0.471374	2.672497	0.001515	0.002873
PPIAP77	0.513941	1.038296	1.014543	4.40E-07	1.81E-06
CDC20	3.875965	17.34762	2.16211	1.31E-31	1.33E-27
IGSF11	0.010372	0.081	2.965262	3.28E-11	3.40E-10
ADH1C	316.5764	150.5887	-1.07194	2.00E-12	2.68E-11
MEP1A	1.078985	5.985123	2.471706	9.57E-13	1.40E-11
RIMS2	0.029662	0.07091	1.257382	0.001241	0.002393
MEIOSIN	0.007514	0.019671	1.388348	4.66E-08	2.37E-07
ZNF320	0.647318	1.45067	1.164174	5.37E-13	8.35E-12
DTL	1.485325	3.592794	1.274328	9.20E-21	8.16E-19
BPIFB2	4.560834	14.23503	1.642076	0.022078	0.032021
SGO2	0.424382	1.220322	1.523826	7.26E-26	3.35E-23
NRBF2P6	0.127469	0.265317	1.05757	8.45E-09	5.04E-08
GABRR1	0.004231	0.025989	2.618755	2.90E-07	1.24E-06
FAM90A1	0.06151	0.185963	1.596124	2.04E-10	1.73E-09
FUT7	0.081164	0.186632	1.201282	0.000103	0.000249
CDKN2A	2.116408	6.107962	1.529073	8.12E-14	1.53E-12
SHCBP1	0.507707	1.562194	1.621506	8.71E-21	7.83E-19
IL17REL	0.009987	0.026371	1.400849	0.00057	0.001179
PRELID3BP4	0.039659	0.090416	1.188944	0.019814	0.029037
INKA2-AS1	0.024094	0.048895	1.020998	7.62E-07	2.97E-06
FCGBP	0.642337	1.708447	1.411282	1.36E-10	1.20E-09
IL1B	0.279866	0.562101	1.006094	1.79E-05	5.14E-05
LINC02159	0.095967	0.221459	1.206424	0.001872	0.003475
U8	0.045952	0.13015	1.50197	0.00336	0.005888
FAM215B	0.007283	0.01467	1.010247	9.10E-07	3.49E-06
IGHV1-45	0.122145	0.31707	1.376204	0.011882	0.018306
KCNH2	0.334758	0.893668	1.416618	1.62E-05	4.72E-05
STK32A	0.024589	0.05904	1.263698	7.82E-05	0.000195
COL9A2	0.723287	1.824404	1.334785	1.02E-13	1.87E-12
RPL7P18	0.027478	0.059593	1.116859	0.00036	0.000778
CRHBP	1.771902	0.798812	-1.14937	2.32E-06	8.13E-06
NFYAP1	0.031942	0.08242	1.367541	2.07E-07	9.13E-07
FAM24B	0.265665	0.83048	1.644335	2.78E-17	1.15E-15

H2AX	11.40762	24.62218	1.109961	2.45E-24	5.99E-22
LINC02613	0.056923	0.125011	1.13498	5.63E-05	0.000145
NRXN1	0.002652	0.007037	1.408109	1.33E-06	4.91E-06
GINS1	1.205925	3.735552	1.631182	2.26E-26	1.48E-23
CD7	1.810743	6.936259	1.937576	3.22E-07	1.37E-06
CELF2-AS1	0.011024	0.095246	3.110988	3.52E-07	1.48E-06
IL37	0.015682	0.048915	1.641157	9.03E-07	3.46E-06
HMGN2P15	0.897095	2.208578	1.299786	3.00E-08	1.59E-07
PCLO	0.029806	0.099166	1.73424	2.07E-10	1.75E-09
H1-7	0.022334	0.093384	2.063913	7.49E-06	2.35E-05
TLR10	0.129745	0.417403	1.685756	0.008507	0.01357
LINC00221	0.401225	1.826177	2.186342	1.91E-11	2.08E-10
MCM8	0.719806	1.661073	1.206436	3.01E-22	3.80E-20
SNORA70	0.006424	0.01513	1.235902	2.31E-09	1.56E-08
LINC01152	0.101587	0.287587	1.501284	0.000169	0.000391
RALYL	0.043001	0.112117	1.382558	4.96E-05	0.000129
KCNJ10	0.113009	0.470902	2.058993	3.93E-06	1.31E-05
SLC34A3	0.04536	0.099896	1.139	0.00041	0.000875
BMP7	0.126686	0.504343	1.993145	0.000318	0.000694
SCAT1	0.027198	0.076394	1.48994	0.001761	0.003292
CHGB	0.111376	0.71504	2.682592	3.33E-09	2.17E-08
C11orf86	0.08759	0.307297	1.810796	0.000483	0.001014
FXYD3	1.636117	4.21109	1.363918	4.84E-07	1.97E-06
CIBAR2	0.016177	0.040533	1.325136	0.015515	0.023294
LYG2	0.08314	0.211223	1.345149	6.20E-08	3.07E-07
CENPH	1.261861	2.969217	1.23453	2.05E-29	8.31E-26
CLDN19	0.220496	0.484378	1.13538	0.000142	0.000334
RNF151	0.023245	0.136497	2.553906	0.003694	0.006412
GSTCD-AS1	0.01453	0.031019	1.094116	2.88E-06	9.87E-06
PLAC1	0.008356	0.088888	3.411154	1.64E-10	1.43E-09
OTOG	0.052927	0.116705	1.140797	5.23E-05	0.000136
FAM225B	0.005567	0.013716	1.300772	0.000747	0.001509
VN1R42P	0.048006	0.097968	1.029107	7.08E-05	0.000179
UGT1A7	0.044445	0.377136	3.085004	0.003496	0.006099
EFNA5	0.245797	1.049414	2.094047	2.20E-05	6.21E-05
PIK3CD-AS2	0.275683	1.181687	2.099764	4.27E-14	8.50E-13
LMNB1	4.054273	10.34555	1.351495	3.41E-23	5.54E-21
MCM2	3.984609	11.29465	1.50313	6.47E-25	1.90E-22
FSIP2-AS1	0.055465	0.119309	1.105065	4.97E-06	1.62E-05
DUSP26	0.091656	0.373679	2.027498	9.09E-06	2.80E-05
SMIM31	0.203091	0.623834	1.619037	4.07E-06	1.35E-05
NCAPG	1.094921	3.396441	1.633197	3.64E-25	1.14E-22
LINC01194	0.082621	0.496502	2.58722	2.56E-11	2.72E-10
PTCH2	0.156729	0.39675	1.339962	0.00419	0.007179

PGC	35.65553	97.09583	1.445284	7.84E-05	0.000195
SPINDOC	2.035629	4.753062	1.223383	3.37E-27	3.80E-24
CEP126	0.047289	0.156235	1.724155	1.51E-11	1.67E-10
TAF4B	0.113083	0.228733	1.016274	0.009611	0.015151
PLEKHG4	0.318971	0.692797	1.119007	9.04E-08	4.31E-07
CLGN	2.42172	6.84762	1.499571	1.23E-10	1.10E-09
CASC19	0.696605	1.507661	1.1139	0.007896	0.012687
KIRREL2	0.028124	2.819373	6.647428	2.09E-06	7.41E-06
LINC02367	0.04108	0.109137	1.40963	1.09E-11	1.24E-10
SLCO4C1	0.53662	1.208672	1.17145	8.63E-09	5.13E-08
LINC02893	0.078108	0.31301	2.002661	3.09E-12	3.96E-11
SYT6	0.01447	0.039814	1.460167	4.43E-06	1.46E-05
EYA1	0.020953	0.132149	2.656937	4.94E-05	0.000129
SEPTIN14	0.004716	0.287264	5.928733	1.03E-09	7.53E-09
MCOLN2	0.175029	0.412041	1.235198	5.34E-05	0.000138
CKM	0.038426	0.161259	2.069228	5.84E-09	3.60E-08
ALX4	0.01592	0.072503	2.187203	0.000542	0.001126
GPR156	0.006498	0.016827	1.372697	0.000407	0.000869
ZNF695	0.038463	0.154596	2.006957	1.02E-11	1.17E-10
C6	66.18464	30.79561	-1.10377	1.59E-15	4.38E-14
HPCA	0.018916	0.071995	1.928308	2.50E-05	6.96E-05
RAB6C-AS1	0.007559	0.023035	1.607595	0.009596	0.015136
PLTP	8.450479	23.06907	1.448855	1.54E-05	4.51E-05
LINC02413	0.104326	0.49822	2.255684	0.00137	0.002622
TLL2	0.030399	0.097455	1.680721	8.14E-07	3.16E-06
PTBP1P	0.008355	0.019175	1.198601	9.30E-06	2.86E-05
SOX12	4.006224	8.263457	1.044502	1.56E-15	4.32E-14
TRNP1	5.374495	17.81824	1.729154	1.41E-13	2.49E-12
MIR210HG	0.820088	2.031573	1.308747	3.36E-14	6.86E-13
CHST2	0.687783	1.475302	1.100984	2.83E-06	9.72E-06
DNER	0.642829	1.699119	1.402281	0.000108	0.000262
LAMB4	0.023176	0.119311	2.364019	4.55E-05	0.00012
TEKT2	0.027593	0.147203	2.415433	1.27E-09	9.13E-09
RDH8	0.097734	0.258852	1.405189	0.000294	0.000646
NELL2	0.175372	0.44811	1.353432	2.15E-06	7.59E-06
B3GALT1-AS1	0.120619	0.367771	1.608351	0.005023	0.008434
HIC2	0.669151	1.482981	1.148097	1.57E-13	2.76E-12
ASIC1	0.381433	0.826524	1.115626	4.87E-07	1.98E-06
SIX2	0.257729	1.039139	2.011461	1.55E-07	7.03E-07
MACROD2	0.257331	0.624676	1.279479	6.14E-07	2.44E-06
DQX1	0.408598	1.322099	1.694075	1.25E-12	1.78E-11
SPNS2	4.142157	1.907536	-1.11867	6.28E-05	0.00016
CDC6	1.485447	4.372938	1.557706	2.67E-23	4.60E-21
DNAI3	0.012145	0.039386	1.697303	0.01983	0.029057

LETM1P2	0.007588	0.034738	2.194716	1.57E-05	4.60E-05
RPL12P25	0.056764	0.196645	1.792546	1.49E-13	2.64E-12
ZFY-AS1	0.391985	0.188163	-1.05882	0.008048	0.012911
VCX3B	0.017809	0.120948	2.763742	8.19E-06	2.55E-05
RIPPLY3	0.205814	0.508155	1.303927	1.27E-06	4.72E-06
LINC01303	0.018479	0.0529	1.517348	1.95E-05	5.57E-05
LINC01357	0.114653	0.278917	1.282568	7.04E-07	2.77E-06
ZAN	0.003053	0.006938	1.184226	0.002335	0.004244
SAA1	1959.516	497.2402	-1.97848	7.83E-06	2.45E-05
KHDC1	0.026921	0.145458	2.433818	2.59E-12	3.40E-11
LRRIQ1	0.005479	0.023528	2.102355	1.29E-08	7.39E-08
TMPRSS3	1.170548	2.646879	1.177108	0.000726	0.001469
PNOC	0.050837	0.130077	1.355402	0.010794	0.016806
SLC1A7	0.86656	3.33755	1.945418	0.00024	0.000537
MYO18B	0.174395	0.664595	1.930121	1.16E-08	6.71E-08
PKIA	0.060165	0.576145	3.259443	2.09E-14	4.53E-13
LINC00540	0.027543	0.186472	2.759195	1.01E-09	7.35E-09
SPC25	1.033708	2.808267	1.441852	1.11E-24	3.10E-22
ZBTB32	0.088311	0.176867	1.002011	6.41E-08	3.16E-07
IL4I1	0.887158	2.945808	1.7314	1.57E-11	1.73E-10
STRIP2	0.274053	0.620705	1.179454	3.96E-11	4.00E-10
PRR11	0.821995	2.549738	1.633146	4.96E-26	2.52E-23
CYP2C9	159.6098	74.2828	-1.10345	6.20E-12	7.49E-11
RIMKLA	0.064818	0.133466	1.042009	0.015766	0.023642
PRKG2	0.010238	0.04669	2.18912	0.000351	0.00076
PRR7-AS1	0.066017	0.146163	1.146659	6.05E-12	7.35E-11
KLRK1-AS1	0.020048	0.063222	1.656966	7.75E-05	0.000194
IL17B	0.036644	0.080031	1.126995	2.08E-07	9.17E-07
TOP2A	3.957568	11.67166	1.560324	1.16E-21	1.28E-19
RUBCNL	0.167977	0.400372	1.253081	2.02E-06	7.18E-06
EMC3-AS1	0.185312	0.432661	1.223282	2.30E-18	1.24E-16
RFPL4B	0.188706	1.133846	2.58701	3.94E-07	1.64E-06
CDX2	0.023322	0.189467	3.022172	0.000339	0.000736
DMBT1	0.052835	1.831412	5.115315	2.85E-08	1.52E-07
NAALAD2	0.13244	0.298654	1.173139	1.50E-10	1.31E-09
BAMBI	11.21765	22.68314	1.01585	1.04E-07	4.91E-07
LINC01293	0.012614	0.054605	2.114015	0.000219	0.000495
MAGEB17	0.669974	1.630784	1.283389	0.000391	0.000839
COX7B2	3.300887	11.36624	1.78383	1.02E-07	4.79E-07
DIPK1C	0.080274	0.182551	1.185291	0.000596	0.001229
UPK1A	0.192876	0.640549	1.731634	0.001201	0.002325
IFI27L2	2.943782	6.702489	1.187026	1.19E-12	1.71E-11
HOXC4	0.0957	0.266365	1.476815	0.000712	0.001444
EFCAB8	0.014308	0.032191	1.169837	0.000224	0.000505

SLC2A1-AS1	0.036855	0.07696	1.062272	0.003402	0.005951
PPP1R14D	0.790115	1.679585	1.087969	0.001761	0.003292
H2BC14	0.027613	0.058766	1.089612	0.000283	0.000624
FAM3D	0.062131	0.305765	2.299032	0.004012	0.006904
ZC2HC1A	0.403536	0.967814	1.262033	1.19E-16	4.19E-15
GPR63	0.018171	0.048861	1.427042	3.19E-05	8.65E-05
SLFN13	0.563103	1.35313	1.264829	1.85E-07	8.27E-07
IGKV3-11	16.46512	44.66912	1.439865	0.034503	0.047899
PAPLN-AS1	0.066095	0.136402	1.045249	1.48E-05	4.35E-05
BBOX1-AS1	0.050396	0.591946	3.554093	2.56E-09	1.71E-08
LINC01234	0.261885	0.632743	1.272684	2.06E-08	1.12E-07
PCDH15	0.002008	0.005743	1.515742	8.79E-08	4.20E-07
DKK1	2.502905	15.81157	2.659305	1.03E-08	6.04E-08
NAT2	6.710575	2.950393	-1.18553	1.79E-07	7.99E-07
TRPV6	0.117519	0.242345	1.044166	0.001033	0.002023
FAM153B	0.023203	0.047597	1.036598	0.001237	0.002387
AKNAD1	0.003633	0.009232	1.345438	1.71E-08	9.55E-08
ADAMTS20	0.004066	0.027744	2.770504	2.01E-13	3.46E-12
LINC02381	1.137784	3.459685	1.604414	0.000155	0.000362
IL21R	0.158048	0.357846	1.178976	2.49E-05	6.93E-05
ACSBG1	0.027253	0.080974	1.571061	0.000168	0.000389
RFLNA	0.307451	1.986255	2.691621	0.021422	0.031161
TULP1	0.004652	0.015983	1.780521	0.000329	0.000716
DTNB-AS1	0.052531	0.142431	1.439025	0.00081	0.001621
ZNF728	0.039459	0.165713	2.070243	1.66E-07	7.46E-07
LRRN4CL	0.092629	0.24663	1.412813	0.01563	0.023458
CACNA1B	0.008723	0.026175	1.585381	0.001116	0.002171
MARK1	0.129996	0.391366	1.590056	0.018201	0.026919
PSMC3IP	0.508293	1.109882	1.126674	4.99E-18	2.50E-16
GRIA2	0.008885	0.049384	2.47464	7.41E-11	6.99E-10
EIF2S3B	0.066176	0.143052	1.112158	0.000362	0.000783
LINC00519	0.052957	0.161406	1.607802	0.003548	0.006182
NEXMIF	0.009241	0.02023	1.13039	0.004884	0.008226
RBFOX3	0.004574	0.0104	1.184969	0.00041	0.000874
WIF1	0.194604	0.513435	1.399638	4.70E-07	1.92E-06
SALL4	0.279706	0.709313	1.342511	6.38E-05	0.000163
HOXC13	0.034748	0.138031	1.98998	2.48E-06	8.65E-06
PRSS44P	0.025664	0.075853	1.56346	2.30E-05	6.46E-05
GALNT8	0.011728	0.05015	2.09629	7.67E-06	2.41E-05
ZNF415	0.201769	0.524833	1.379157	8.79E-05	0.000217
MICAL1	1.539651	3.219748	1.064344	8.82E-10	6.55E-09
CCDC183	0.297042	0.822336	1.469063	1.49E-13	2.64E-12
KLHDC7B	0.555889	1.33019	1.258764	0.005256	0.008787
CADM2	0.04489	0.106713	1.249269	3.48E-13	5.60E-12

IGLV3-21	9.43759	29.26245	1.63256	0.022537	0.032614
DHDH	0.300217	0.777109	1.372112	5.49E-17	2.08E-15
COL9A3	0.843029	2.060239	1.289158	0.000221	0.000498
C1orf158	0.006494	0.058442	3.169843	6.32E-08	3.12E-07
HSPA8P8	0.060687	0.151088	1.315925	0.000748	0.00151
APOL4	0.615592	1.322984	1.103749	7.31E-07	2.86E-06
DNASE1L3	6.432773	3.094206	-1.05587	6.50E-13	9.92E-12
IGDCC3	0.075308	0.478301	2.667045	0.000517	0.00108
LINC01608	0.469697	1.450715	1.626962	1.01E-10	9.25E-10
TMEM158	0.437717	1.582338	1.853986	0.000632	0.001297
FAM218A	0.019121	0.060881	1.670831	5.34E-07	2.15E-06
CD70	0.117191	0.531743	2.181865	3.15E-05	8.54E-05
FAM183A	0.18193	0.577789	1.667162	4.99E-10	3.88E-09
SV2A	0.189608	0.706097	1.896848	1.36E-09	9.74E-09
KIF15	0.352801	1.254596	1.830298	3.91E-26	2.20E-23
E2F2	0.282707	0.847667	1.584192	3.29E-25	1.11E-22
ANXA8	0.046768	0.193921	2.051884	0.027389	0.038847
ZNF43	0.312657	0.748761	1.259925	2.96E-11	3.10E-10
TUBAP2	0.084845	0.171735	1.017289	5.70E-14	1.10E-12
MATN1	0.015015	0.041229	1.457248	7.71E-08	3.74E-07
PARPBP	0.445072	1.125917	1.338991	4.40E-22	5.28E-20
XKR4	0.002041	0.00981	2.264712	7.95E-05	0.000198
CFHR4	19.48851	6.800673	-1.51887	4.05E-17	1.62E-15
IGFBPL1	0.282392	0.882687	1.644202	4.18E-06	1.39E-05
EME1	0.414667	1.215107	1.551058	7.54E-28	1.39E-24
FABP5P7	0.083241	0.204323	1.295484	4.80E-06	1.57E-05
ISM2	0.011628	0.129844	3.481131	6.06E-05	0.000155
JPT1	9.937859	20.03823	1.011748	1.36E-17	6.17E-16
PLAUR	1.138896	2.596424	1.18889	1.18E-10	1.06E-09
CHGA	0.044644	4.251296	6.5733	2.56E-06	8.89E-06
CLEC18B	0.03254	0.070185	1.108956	1.24E-05	3.72E-05
SYNGR4	0.133449	0.426353	1.675755	5.25E-05	0.000136
WDR87BP	0.006631	0.036078	2.443845	1.26E-06	4.68E-06
AKR1B1	4.857642	11.17851	1.2024	1.80E-06	6.47E-06
GLRA3	0.002955	0.008406	1.508438	5.51E-06	1.78E-05
ZNF257	0.068455	0.296617	2.115378	1.01E-16	3.63E-15
LINC02910	0.022893	0.054229	1.244153	0.002926	0.005204
SPDL1	0.958135	2.09709	1.130088	1.32E-24	3.57E-22
SH3BP1	0.883849	1.933692	1.129487	5.53E-14	1.07E-12
MMP9	5.920261	12.52735	1.081349	2.55E-06	8.86E-06
KIFC1	2.656517	7.670825	1.529846	2.50E-24	6.04E-22
PDZD3	0.038754	0.103857	1.422194	0.000101	0.000246
RRAD	1.268993	2.689031	1.083402	0.003044	0.005386
TRBV30	0.180589	0.362239	1.004232	0.006312	0.01036

DLL3	0.009435	0.13074	3.792492	6.25E-11	6.00E-10
CENPL	0.62758	1.355299	1.11074	4.88E-20	3.75E-18
GRM4	0.0039	0.021238	2.445117	1.08E-07	5.08E-07
DLK2	0.48652	1.151926	1.243477	8.04E-13	1.20E-11
MEG3	1.337612	3.519993	1.395913	0.00495	0.008323
KMO	4.479757	2.218225	-1.01401	4.81E-11	4.75E-10
MAGEA1	3.313699	7.930815	1.259027	0.000427	0.000908
LCTL	0.040071	0.110405	1.462171	3.10E-07	1.32E-06
BCL11A	0.100319	0.26902	1.423118	1.89E-05	5.41E-05
FILNC1	0.0131	0.068708	2.390905	0.000668	0.001363
MDGA2	0.035244	0.140978	2.00004	2.51E-10	2.08E-09
CNTNAP4	0.117296	0.381568	1.701787	9.77E-08	4.62E-07
EPHB6	0.917304	2.158573	1.234607	3.66E-06	1.23E-05
GYS2	16.77404	8.214798	-1.02993	8.45E-11	7.89E-10
TMCC1-DT	0.172577	0.376793	1.126528	1.64E-12	2.26E-11
GLYATL2	0.013964	0.042891	1.61896	2.07E-06	7.35E-06
LINC00943	0.014719	0.045634	1.632405	1.40E-07	6.39E-07
DYDC1	0.03801	0.08175	1.104844	0.002106	0.003862
TPM2	7.663524	16.88595	1.139743	1.49E-07	6.77E-07
RPS6KA6	0.044157	0.101752	1.204336	0.007419	0.012
MLLT11	1.059777	2.346984	1.147047	1.85E-05	5.31E-05
EGF	0.143558	0.346474	1.271113	0.000239	0.000536
KBTBD11-OT1	0.110332	0.052525	-1.07076	2.05E-06	7.29E-06
LINC00323	0.027271	0.072752	1.415616	0.007174	0.011635
BECN2	0.013878	0.044627	1.685057	1.11E-07	5.17E-07
KIF20A	1.496296	4.456665	1.574569	1.59E-22	2.15E-20
AQP1	49.02188	18.65939	-1.39352	0.000282	0.000622
NAP1L3	0.185038	0.407751	1.139867	8.85E-05	0.000218
KAZALD1	0.530083	1.328197	1.325181	1.17E-08	6.78E-08
LINC01666	0.127168	0.326264	1.359297	2.81E-05	7.71E-05
FGF9	0.0086	0.065633	2.932077	0.000107	0.00026
CYP4F23P	0.106599	0.215204	1.013512	7.10E-06	2.24E-05
SNORA73B	0.737231	1.53324	1.056395	5.68E-11	5.51E-10
CNGB1	0.04094	0.150846	1.881507	0.000574	0.001187
YWHAEP1	0.078828	0.290136	1.879954	2.51E-06	8.74E-06
RPSAP63	0.01052	0.023098	1.134621	0.00019	0.000435
DRP2	0.010485	0.034171	1.704452	1.70E-12	2.32E-11
PLOD2	7.610629	15.41523	1.018269	5.94E-20	4.54E-18
UBE2C	6.193763	21.00566	1.76189	2.04E-23	3.72E-21
TH2LCRR	0.048742	0.138145	1.502946	0.003684	0.006397
SLC6A14	0.048206	0.422808	3.132715	2.91E-10	2.39E-09
KLK1	0.025324	0.099417	1.972956	1.51E-06	5.50E-06
ZNF676	0.132094	0.391576	1.567721	0.000104	0.000254
SNORD3B-1	0.021417	0.079744	1.896623	3.35E-09	2.18E-08

CPA4	0.062373	0.16627	1.414526	0.011037	0.017148
FBN2	0.023177	0.057678	1.315346	0.000237	0.000532
TSKS	0.041927	0.149571	1.834895	0.000206	0.00047
LINC01585	0.010526	0.027521	1.386551	6.96E-06	2.21E-05
BUB1B	0.795851	2.45534	1.625352	5.42E-23	8.35E-21
ZNF334	0.166672	0.360735	1.113928	0.002001	0.00369
FAM72A	0.093162	0.22842	1.293883	2.21E-19	1.56E-17
NLRP2	0.205766	0.474464	1.205294	1.67E-06	6.04E-06
ZNF781	0.122812	0.400986	1.707104	2.91E-12	3.76E-11
PRUNE2	0.081298	0.264937	1.704361	0.002149	0.003933
TRPC3	0.042138	0.162742	1.94939	2.20E-06	7.77E-06
PSRC1	1.083913	2.908284	1.42392	1.54E-27	2.23E-24
ADAMTS6	0.066869	0.15067	1.171975	1.58E-11	1.73E-10
GUCY1B2	0.100951	0.227669	1.173284	9.98E-11	9.17E-10
RPS2P4	0.068872	0.14314	1.05544	0.003425	0.005988
TMEM52B	0.116439	0.289827	1.315615	9.49E-06	2.92E-05
AGR2	6.013021	18.64841	1.632891	3.39E-06	1.15E-05
DACH2	0.019372	0.042725	1.141104	1.62E-09	1.14E-08
NT5DC4	0.019244	0.057777	1.586114	4.38E-09	2.77E-08
S100A1	2.055351	5.227553	1.346751	0.00112	0.002177
MTFR2	0.363929	1.100443	1.596357	9.68E-24	1.95E-21
LMO3	0.024162	0.090541	1.905823	9.82E-15	2.29E-13
IL10	0.093094	0.189848	1.028089	0.005559	0.009243
SPATA17	0.053548	0.133264	1.315367	2.24E-07	9.84E-07
EFHC2	0.015332	0.135149	3.139953	1.61E-12	2.21E-11
NR5A1	0.02313	0.059833	1.371145	0.006944	0.011288
EPHX4	0.090116	0.237585	1.398589	2.91E-08	1.55E-07
STOX1	0.243895	0.593976	1.284144	3.47E-11	3.56E-10
PHC2-AS1	0.044356	0.115276	1.377892	7.87E-07	3.06E-06
LRCOL1	4.11732	1.859822	-1.14654	3.39E-08	1.78E-07
SNHG26	0.173382	0.381251	1.13679	3.38E-08	1.77E-07
NPM1P9	0.037563	0.095658	1.34856	1.96E-11	2.13E-10
CAPN9	0.163326	0.331964	1.02327	0.003721	0.006455
SLC6A8	3.823352	8.727808	1.190781	0.000277	0.000612
LAG3	1.003906	2.375911	1.242857	1.47E-05	4.32E-05
SALL2	0.574354	1.421217	1.307113	2.05E-14	4.46E-13
LVRN	0.015695	0.052042	1.729341	1.17E-05	3.51E-05
HTR3A	0.035947	0.787226	4.45283	5.11E-05	0.000133
PCDH9	0.027264	0.096181	1.818775	0.032568	0.04547
CASKIN1	0.031702	0.086687	1.451249	0.000446	0.000944
LINC01361	0.007956	0.336879	5.40404	8.34E-11	7.80E-10
PCDHA1	0.092653	0.201943	1.124037	8.13E-06	2.53E-05
AUNIP	0.192844	0.599617	1.63661	3.54E-24	7.89E-22
HYAL4	0.010282	0.047465	2.206702	3.93E-06	1.31E-05

ZBTB12	1.209308	2.691801	1.15439	6.95E-18	3.34E-16
HOXB8	0.024982	0.177182	2.826278	0.009727	0.015315
NCAPD2P1	0.125242	0.540397	2.109297	9.33E-10	6.89E-09
PKIA-AS1	0.009784	0.067114	2.778055	8.29E-07	3.21E-06
ADAM32	0.014065	0.040379	1.521474	0.001055	0.002063
RPL34P33	0.083542	0.216766	1.375564	0.000283	0.000625
CARMIL3	0.168444	0.34113	1.018049	0.000404	0.000864
OLFM4	0.961701	4.411668	2.197664	0.015374	0.023101
FOXM1	2.14173	5.822842	1.442947	9.87E-19	5.92E-17
LINC02416	0.0427	0.117894	1.465171	3.85E-05	0.000102
AURKBP1	0.218706	0.104816	-1.06113	0.008064	0.012932
ARF4P2	0.083743	0.256915	1.617259	6.47E-07	2.56E-06
PDE7A-DT	0.052777	0.120036	1.185489	7.11E-11	6.73E-10
MYADML2	0.01275	0.039873	1.644905	2.43E-08	1.31E-07
TRPM5	0.019381	0.067533	1.800952	2.81E-09	1.86E-08
LINC02152	0.031436	0.134175	2.093637	7.02E-05	0.000177
KBTBD11	1.054962	0.501186	-1.07377	3.39E-07	1.43E-06
ANKRD36BP2	0.026868	0.079234	1.560227	0.001251	0.002411
DPY19L2P2	0.056604	0.141055	1.317281	1.62E-10	1.41E-09
FARP1-AS1	0.046165	0.132217	1.518044	6.91E-06	2.19E-05
IGHG1	101.706	352.109	1.791618	0.001424	0.002716
SLC30A8	0.002401	0.096369	5.326652	1.51E-09	1.06E-08
IGHV1-24	3.832581	14.8074	1.949931	0.012774	0.019547
SPACA7	0.153024	0.058405	-1.3896	8.81E-05	0.000217
LINC01841	0.018191	0.040593	1.157974	0.014137	0.021426
DPP6	0.004404	0.012267	1.477912	9.50E-06	2.92E-05
MCM5	5.355531	11.68658	1.125752	1.02E-24	2.87E-22
RAET1K	0.031381	0.095113	1.59974	9.97E-13	1.45E-11
CLCN3P1	0.015077	0.0555	1.880098	0.0003	0.000658
ITPRIPL1	0.137742	0.298381	1.115187	1.25E-05	3.74E-05
SMTNL2	0.048298	0.145565	1.591627	3.85E-05	0.000102
RGS22	0.006726	0.026214	1.962581	0.006021	0.009929
TCF24	0.004532	0.046285	3.352431	2.59E-14	5.50E-13
RPS26P47	0.140911	0.298683	1.083831	0.00441	0.007515
HSD11B1	258.0507	127.7172	-1.0147	5.75E-08	2.87E-07
LINC01063	0.192714	0.405836	1.074433	1.25E-05	3.73E-05
OTOGL	0.00729	0.024772	1.764712	0.000126	0.000301
ARL14	0.743928	3.007341	2.015254	0.000344	0.000747
PTGS1	0.616528	1.277466	1.051045	6.68E-06	2.12E-05
BIRC7	0.087863	1.107622	3.656071	2.01E-09	1.38E-08
ZNF488	0.023284	0.053339	1.195828	1.11E-07	5.19E-07
LINC02041	0.596879	1.608552	1.430251	2.75E-09	1.82E-08
HFM1	0.010148	0.038262	1.91475	0.00024	0.000538
POLQ	0.180849	0.521321	1.527384	1.48E-23	2.83E-21

C6orf223	0.777348	2.691377	1.791712	2.78E-12	3.62E-11
ERFE	0.290543	0.657172	1.177516	2.69E-09	1.79E-08
FFAR2	0.124336	0.359645	1.532334	7.41E-07	2.90E-06
NBEAP1	0.01126	0.038883	1.787989	0.006533	0.010681
LINC01094	0.111764	0.238691	1.09469	8.09E-07	3.14E-06
SCIN	0.063717	0.131369	1.043878	1.79E-05	5.16E-05
SOHLH2	0.04263	0.374583	3.135344	5.05E-09	3.16E-08
VN1R85P	0.095936	0.321021	1.742524	2.63E-07	1.13E-06
ARHGAP29-AS1	0.059392	0.15708	1.403157	0.000947	0.001867
PIFO	0.046492	0.209158	2.169537	2.26E-13	3.82E-12
SPINK4	0.105297	0.993598	3.238204	0.000119	0.000285
LAMP5	0.479045	2.361968	2.301758	3.68E-05	9.83E-05
ZNF239	0.380887	1.088397	1.514771	3.09E-14	6.39E-13
PAFAH1B3	7.864026	20.1849	1.359936	1.64E-17	7.31E-16
DCAF4L1	0.063638	0.171305	1.428606	3.38E-07	1.43E-06
MYBPC2	0.022446	0.162568	2.856533	4.95E-06	1.62E-05
RNASE2	0.266916	1.299638	2.283649	4.15E-06	1.38E-05
SKA1	0.930642	3.11114	1.741146	4.25E-26	2.21E-23
RPL18P13	0.284753	0.813428	1.514306	2.59E-10	2.14E-09
BSND	0.010799	0.075055	2.79706	9.21E-13	1.36E-11
RNF175	0.056575	0.141883	1.326452	0.000145	0.000341
DNAJB13	0.029754	0.072329	1.281498	1.38E-10	1.22E-09
NUDT1	3.332228	7.169853	1.105457	4.02E-23	6.33E-21
CD27	1.085997	2.419575	1.155733	0.00016	0.000374
MAGEA12	0.728133	5.372457	2.883309	1.15E-10	1.04E-09
GLIS1	0.016283	0.05511	1.758989	0.001069	0.002086
LPAR3	0.117656	0.336336	1.51533	0.000314	0.000686
LINC02253	0.100365	0.513281	2.354493	8.71E-09	5.18E-08
PLPP7	0.184969	0.584476	1.659864	0.000132	0.000313
TGFB2-AS1	0.184693	0.51946	1.491879	0.004208	0.007205
GSDME	0.739933	1.532201	1.050139	8.87E-13	1.32E-11
WNT6	0.166791	0.783529	2.231941	0.030723	0.043094
CDC7	0.745627	1.987546	1.414463	2.65E-24	6.26E-22
CSTF3-DT	0.015627	0.037392	1.258719	4.10E-12	5.14E-11
MPP2	0.078406	0.219501	1.4852	3.75E-11	3.81E-10
RPL32P32	0.069708	0.171606	1.299699	9.94E-10	7.28E-09
KPNA2	12.99762	27.09531	1.059796	4.88E-20	3.75E-18
TTK	0.592091	2.016243	1.767779	3.33E-24	7.56E-22
IGSF3	1.581155	3.190286	1.012707	3.03E-12	3.90E-11
RLN2	0.013395	0.037371	1.480223	0.001683	0.003159
SPATA3-AS1	0.020672	0.059402	1.522805	3.06E-12	3.93E-11
EIF5A2	0.877613	1.802089	1.038013	1.66E-07	7.48E-07
FRMD5	0.0175	0.043386	1.309854	1.33E-12	1.88E-11

NSUN7	0.156747	0.353059	1.171473	0.000289	0.000636
ISL2	0.072184	0.383904	2.410986	1.05E-09	7.61E-09
CALCR	0.009753	0.153011	3.971696	3.75E-14	7.52E-13
RGSL1	0.393657	0.173643	-1.18082	0.000117	0.000282
PAGE1	1.695226	19.18107	3.500134	3.64E-16	1.16E-14
LINC01597	0.006873	0.014993	1.125312	0.001416	0.002703
FSIP1	0.076158	0.194838	1.355208	3.74E-07	1.56E-06
PPP4R3C	0.368784	0.980681	1.411006	7.19E-06	2.27E-05
CERS3	0.005812	0.014672	1.335857	0.002091	0.003837
LINC02211	0.030529	0.255643	3.065881	8.16E-09	4.89E-08
UROC1	16.34587	7.601672	-1.10454	1.58E-05	4.62E-05
PAPLN	1.288653	3.103659	1.268107	1.61E-07	7.27E-07
NECTIN1-DT	0.129397	0.271725	1.070345	4.16E-06	1.38E-05
PTHLH	0.537318	2.01562	1.907374	1.32E-08	7.53E-08
TMEM59L	0.073851	0.179132	1.278346	0.009527	0.015039
EMP3	5.171408	11.41898	1.142805	1.53E-06	5.56E-06
C16orf89	0.117774	4.419463	5.229778	1.89E-06	6.76E-06
LINC02475	0.112573	0.375796	1.739083	1.35E-08	7.69E-08
KCNQ3	0.033636	0.073398	1.125752	0.000776	0.001559
ZSCAN5DP	0.015895	0.034283	1.108922	0.000507	0.00106
TFF2	1.112789	8.189273	2.879555	1.40E-10	1.24E-09
NPAS1	0.164459	0.509899	1.632485	2.14E-10	1.80E-09
IL17RD	0.082035	0.181291	1.144005	0.006727	0.010968
SLC27A5	65.70058	32.57834	-1.01199	4.53E-14	8.99E-13
MYOSLID	0.021605	0.077066	1.834708	0.000215	0.000488
SEMA3E	0.079415	0.609526	2.940198	7.73E-15	1.84E-13
GABRB1	0.016683	0.034535	1.049677	1.27E-05	3.77E-05
PIMREG	0.323516	1.116404	1.78695	7.13E-15	1.72E-13
CENPM	2.029164	6.475783	1.674169	1.26E-26	1.07E-23
C9	123.7752	54.23485	-1.19043	0.000978	0.001924
HIF1A-AS3	0.042657	0.100615	1.23801	0.000415	0.000885
ACTA1	0.134205	0.318487	1.246792	2.13E-07	9.38E-07
OIP5	1.061762	2.817846	1.408133	1.09E-20	9.46E-19
LINC01833	0.111635	0.278078	1.316706	2.08E-05	5.89E-05
OR52K3P	0.108988	0.277017	1.345805	0.000285	0.000627
GAL	0.27693	0.593795	1.100443	0.012361	0.018973
KCND3	2.141947	1.057692	-1.018	1.34E-08	7.64E-08
NANOS3	0.062965	0.138498	1.137238	0.002241	0.004088
COL19A1	0.005771	0.019693	1.770885	0.013277	0.02024
GDF10	0.1375	0.650351	2.241792	4.96E-07	2.02E-06
KCNA2	0.006598	0.023516	1.833657	0.010297	0.016113
DPYSL4	0.180424	0.42044	1.220513	0.000314	0.000686
MIR6797	0.246165	0.509847	1.050435	1.64E-06	5.94E-06
OSMR-DT	0.085362	0.171414	1.005812	2.49E-05	6.95E-05

THBS1-IT1	0.142552	0.29835	1.065515	1.32E-05	3.92E-05
LRP12	0.268553	0.786307	1.549886	8.46E-10	6.31E-09
C17orf99	0.014908	0.035671	1.258695	7.88E-10	5.92E-09
ARNILA	0.038438	0.077236	1.006735	2.92E-07	1.25E-06
MIR3150BHG	0.009353	0.023568	1.33334	4.79E-07	1.95E-06
DNAH17-AS1	0.004203	0.012709	1.596376	0.00015	0.000352
LYPD6B	0.2073	0.746741	1.848886	1.05E-08	6.13E-08
SLC25A21	0.034183	0.083177	1.282921	3.30E-05	8.92E-05
MYH15	0.007603	0.01952	1.360401	2.61E-05	7.24E-05
LINC02768	2.006166	0.889993	-1.17258	3.43E-09	2.22E-08
LINC02391	0.039396	0.080571	1.032194	8.31E-08	3.99E-07
BNIP3P10	0.039525	0.087567	1.147617	3.09E-09	2.03E-08
C4BPA	578.5932	285.057	-1.0213	3.73E-14	7.49E-13
CD177	0.106772	0.804691	2.913906	0.00253	0.004565
GRASLND	0.056609	0.196529	1.795653	4.47E-07	1.84E-06
BTG1-DT	0.029511	0.080834	1.453714	9.28E-12	1.08E-10
GAL3ST4	0.585308	1.182217	1.014227	5.81E-05	0.000149
PLEKHS1	0.162514	0.421707	1.375674	7.57E-08	3.68E-07
GABRB3	0.278188	0.565004	1.022201	0.002629	0.004725
ZNF660	0.071551	0.165675	1.211319	1.63E-06	5.92E-06
DUXAP8	0.105556	0.214755	1.024679	3.40E-11	3.50E-10
ZNF66	0.073761	0.196387	1.412771	3.24E-12	4.14E-11
GPAT2	0.227717	0.734472	1.689467	0.003054	0.005401
ORC1	0.705153	2.131822	1.596079	1.20E-26	1.06E-23
FKBP10	3.658578	13.06117	1.835928	1.42E-06	5.22E-06
IGHV3-30	5.52639	16.93784	1.61584	0.024613	0.035276
PGLYRP4	0.008506	0.047687	2.487119	4.83E-05	0.000126
TMEM35A	0.031357	0.113244	1.852587	0.000131	0.000312
IGFBP3	25.6896	53.19441	1.050091	1.22E-08	7.03E-08
LINC01605	0.101416	0.247519	1.287252	0.007472	0.012073
GDPD3	0.588737	1.263694	1.101953	7.79E-09	4.68E-08
ADGRB2	0.079255	0.358818	2.178671	1.26E-05	3.76E-05
CYP2B6	38.35836	18.79803	-1.02896	8.54E-05	0.000211
LINC01088	0.015897	0.035659	1.165572	1.24E-06	4.60E-06
CGAS	0.356356	0.733373	1.04123	9.60E-05	0.000235
DLX1	0.022758	0.332711	3.869809	3.79E-07	1.58E-06
HTR1F	0.008898	0.031767	1.83601	0.003589	0.006247
C19orf84	0.028902	0.090697	1.64986	7.13E-07	2.79E-06
C16orf95-DT	0.593111	1.353534	1.190357	1.55E-07	7.04E-07
PFKFB4	0.396721	1.126928	1.506198	1.67E-18	9.41E-17
LPO	0.004435	0.032421	2.86981	3.37E-07	1.42E-06
KIF18B	0.745086	2.415452	1.696815	1.47E-24	3.88E-22
KLHL41	0.052916	0.116078	1.133328	0.010125	0.015863
CFAP92	0.023751	0.051443	1.115015	0.002232	0.004073

LINC02323	0.081702	0.222378	1.444572	8.09E-09	4.84E-08
BRINP3	0.042923	0.252532	2.556634	3.14E-09	2.05E-08
MYBL2	3.147466	15.50265	2.300252	1.18E-30	7.97E-27
PTGER4	0.628316	1.274849	1.020763	1.21E-06	4.52E-06
KRTAP5-7	0.048362	0.122546	1.341389	0.007494	0.012103
HJURP	1.194339	3.602775	1.592897	7.42E-24	1.51E-21
CFAP61	0.011972	0.047794	1.997215	1.17E-10	1.06E-09
E2F8	0.541187	1.302658	1.26726	6.37E-17	2.38E-15
MMP12	1.146765	2.489572	1.118328	1.59E-08	8.94E-08
RAD51AP1	1.017277	2.512215	1.304248	8.67E-19	5.29E-17
MMP16	0.036863	0.092089	1.320847	3.98E-06	1.33E-05
SGSM1	0.128373	0.259406	1.014866	2.07E-05	5.88E-05
FRAS1	0.363966	0.881711	1.276502	2.22E-07	9.76E-07
SBSN	0.032289	0.344221	3.414197	4.84E-05	0.000127
RASGEF1C	0.018774	0.053839	1.519894	0.003856	0.00666
ZNF727	0.088409	0.196456	1.151939	5.94E-07	2.37E-06
PTPRN	0.008178	0.026474	1.694814	6.54E-09	3.99E-08
TCL6	0.014682	0.071053	2.274872	1.69E-11	1.85E-10
CDC25A	0.52143	1.728404	1.728895	3.72E-26	2.16E-23
PRSS2	0.178941	15.72391	6.457336	0.016146	0.024169
CCDC144NL-AS1	0.088176	0.261918	1.570654	3.60E-06	1.21E-05
HSPA6	2.770011	5.899295	1.090651	5.83E-09	3.60E-08
FANCE	1.005905	2.294123	1.189449	1.89E-22	2.47E-20
CLIC3	0.774715	1.826149	1.237067	8.52E-08	4.09E-07
CHEK1	0.794013	1.857673	1.226262	2.29E-23	3.97E-21
LINC02577	0.002702	0.076171	4.817138	6.39E-09	3.91E-08
SH2D7	0.006886	0.023639	1.779358	1.40E-07	6.41E-07
C19orf33	2.382145	4.788434	1.007292	0.009213	0.014589
ZNF860	0.035121	0.078645	1.163022	0.000423	0.0009
PLCB1	0.676066	1.499806	1.149541	3.95E-17	1.59E-15
DEPDC1B	1.029942	2.397388	1.2189	3.22E-22	4.03E-20
KHSRPP1	0.010258	0.027025	1.397546	0.000461	0.000974
EVX1	0.082566	0.225268	1.448021	0.006222	0.010227
TENM3	0.044852	0.162758	1.859479	0.021249	0.030928
PSAPL1	0.054458	0.142626	1.38902	0.001093	0.00213
NDRG1	15.30895	36.05293	1.235741	4.50E-08	2.30E-07
TCAM1P	0.088843	0.240614	1.437388	0.001042	0.002039
GPRC5D	0.16502	0.365782	1.148346	2.95E-14	6.16E-13
HSD3BP4	0.0232	0.079181	1.771013	0.004262	0.007286
FOXD1	0.037557	0.175391	2.22343	0.000934	0.001845
HACD1	0.234469	0.621927	1.407349	0.03028	0.042523
CDHR4	0.016591	0.044989	1.439117	7.21E-07	2.82E-06
XRCC2	0.304134	0.847984	1.479328	9.57E-23	1.39E-20

TRAM1L1	0.241745	0.819412	1.761102	6.27E-12	7.57E-11
GCSAML	0.018597	0.093741	2.333599	2.95E-06	1.01E-05
LINC00565	0.01548	0.061201	1.983116	0.011177	0.017343
MRPL45P1	0.054005	0.127958	1.244513	4.45E-05	0.000117
FGD5P1	0.008987	0.048228	2.424002	8.50E-07	3.28E-06
ESCO2	0.200447	0.44757	1.158895	6.60E-14	1.26E-12
HOXD1	0.074838	0.230134	1.620635	0.000617	0.001268
NCOA7-AS1	0.145441	0.05522	-1.39716	0.001847	0.003434
SZT2-AS1	0.030781	0.075224	1.289171	3.46E-10	2.78E-09
PPFIA4	0.042414	0.166376	1.971829	4.16E-17	1.65E-15
ABCG4	0.014141	0.044839	1.664886	0.019409	0.028496
DDN	0.016956	0.073454	2.115047	3.23E-11	3.36E-10
CACNG8	0.019756	0.040007	1.017959	0.000984	0.001935
RGS16	3.498616	9.001695	1.363412	0.026839	0.038166
TYMS	5.795243	11.91981	1.04042	2.16E-17	9.36E-16
SPESP1	0.433204	1.213464	1.486012	0.000229	0.000516
SLC22A8	0.044551	0.24679	2.46976	0.000335	0.000729
NAALADL1	0.703708	2.942544	2.064015	2.00E-09	1.37E-08
PCDHA5	0.006638	0.022276	1.746673	3.03E-05	8.27E-05
CLDN11	0.391247	0.839809	1.101982	0.000234	0.000527
CCNF	0.854644	2.119116	1.310068	2.38E-25	9.10E-23
GFY	0.008324	0.057426	2.786391	2.61E-09	1.74E-08
MARCKSL1	27.26708	59.92181	1.135921	4.59E-14	9.09E-13
CDH7	0.003398	0.023469	2.787998	0.00118	0.002287
LRRC39	0.23231	0.630451	1.440332	1.52E-08	8.59E-08
CDCA2	0.339717	1.087511	1.678625	6.00E-22	7.13E-20
DUSP13	0.088744	0.284678	1.681615	0.000342	0.000742
GAD1	0.08829	0.17911	1.020534	0.00483	0.008144
SLC44A5	0.424955	1.272347	1.582111	1.17E-09	8.42E-09
PLAU	2.390377	5.623797	1.234306	0.000179	0.000413
LINC01970	0.074532	0.177804	1.254358	7.44E-06	2.34E-05
PPIAP34	0.102098	0.226047	1.14667	6.00E-06	1.93E-05
ZIC2	1.128634	2.672536	1.243631	6.62E-10	5.03E-09
OSR2	0.302642	0.873015	1.528393	8.40E-06	2.61E-05
WASF1	1.929759	4.257836	1.1417	2.48E-18	1.33E-16
ANLN	1.179861	3.461096	1.552612	1.20E-20	1.02E-18
SPAG5	3.5691	7.744627	1.117635	4.22E-17	1.66E-15
PPIAP45	0.049694	0.21665	2.124232	7.91E-11	7.45E-10
CDC20P1	0.035968	0.094996	1.401141	3.73E-07	1.56E-06
FABP7	0.007131	0.77211	6.758541	0.000522	0.001089
LINC00622	0.154165	0.36272	1.234383	3.44E-08	1.80E-07
KCNK2	0.077301	0.289774	1.906365	4.35E-06	1.44E-05
ARFGEF3	0.443436	0.890271	1.005517	1.35E-05	3.99E-05
MYBPC3	0.020347	0.129674	2.671967	1.21E-05	3.63E-05

UPK1B	0.021951	0.127573	2.538952	0.001659	0.003117
TRBV9	0.250767	0.640748	1.353409	0.003044	0.005386
CD79A	1.246988	3.714237	1.574619	0.036107	0.0498
C15orf48	3.616534	7.522155	1.056538	9.69E-07	3.70E-06
ZBED2	0.097306	0.264745	1.444008	5.17E-07	2.09E-06
VNN2	4.469249	8.989017	1.008131	3.86E-07	1.61E-06
SLC16A3	1.575867	4.594133	1.543647	1.09E-12	1.58E-11
ZNF711	0.209923	0.700992	1.739538	1.40E-11	1.56E-10
RADX	0.405988	0.837573	1.044776	3.63E-08	1.89E-07
LINC01460	0.00522	0.020176	1.95037	0.001515	0.002874
SVOPL	0.004165	0.025169	2.595385	1.50E-06	5.47E-06
POTEE	0.002906	0.010685	1.878309	1.96E-07	8.72E-07
P3H4	2.597819	5.541152	1.092885	1.61E-12	2.22E-11
RDM1	0.290198	0.673772	1.215225	1.02E-15	2.98E-14
CHTF18	1.195772	2.643699	1.144616	2.82E-25	1.03E-22
PLEKHH2	0.148082	0.3655	1.303477	2.24E-08	1.22E-07
MAP3K9-DT	0.539529	1.456115	1.432352	3.89E-22	4.78E-20
ERCC6L	0.2082	0.603695	1.535851	1.48E-21	1.58E-19
LNCSRLR	0.178121	0.379257	1.090318	3.18E-11	3.31E-10
C5orf46	0.381661	0.802606	1.072399	2.19E-05	6.19E-05
HOXA11-AS	0.033559	0.156184	2.218461	2.15E-06	7.61E-06
IGHVII-78-1	0.049493	0.121885	1.300218	1.14E-06	4.28E-06
TBX18	0.038831	0.236861	2.608763	0.000126	0.000301
MYB	0.068726	0.199709	1.538962	4.06E-17	1.62E-15
CDC45	1.242585	3.491983	1.490702	3.39E-25	1.11E-22
CPA2	0.022863	7.193458	8.297549	9.89E-08	4.67E-07
RGS2	5.386792	15.60871	1.534853	4.04E-09	2.58E-08
BLMH	3.452638	6.931595	1.005488	2.70E-11	2.85E-10
PSLNR	0.018663	0.175949	3.236903	6.58E-12	7.90E-11
CLSPN	0.204019	0.623131	1.610832	1.87E-19	1.34E-17
CFAP74	0.028111	0.076503	1.444407	0.000818	0.001636
DEPDC1	0.454848	1.53713	1.756782	2.60E-24	6.21E-22
DNAH3	0.012933	0.026606	1.040686	2.69E-13	4.46E-12
CHMP1B2P	0.018453	0.047347	1.359455	3.83E-07	1.59E-06
CHRDL1	0.142805	0.835824	2.549149	0.000603	0.001241
ROCR	0.121295	0.279776	1.205749	0.002205	0.004029
NFE4	0.053338	0.441216	3.048253	5.56E-06	1.80E-05
KHDC1-AS1	0.024556	0.076613	1.641532	4.19E-11	4.20E-10
PBK	1.771614	4.389764	1.309079	2.38E-16	7.82E-15
EIF4A2P1	0.017976	0.036789	1.033194	0.031889	0.0446
FZD2	0.411587	0.972976	1.241205	0.000123	0.000295
TMEM201	1.06747	2.228933	1.062158	9.62E-27	8.88E-24
GABRQ	0.02936	0.112131	1.933235	4.17E-11	4.19E-10
KCNQ1OT1	0.017803	0.04216	1.243753	6.13E-14	1.18E-12

MKRN3	0.076438	0.187857	1.297279	4.60E-09	2.90E-08
REC114	0.025669	0.219512	3.096213	0.005662	0.009396
ATP6V1FNB	0.158642	0.458161	1.530083	9.98E-07	3.79E-06
SOX2	0.138027	0.704263	2.351169	0.006346	0.010407
MACROH2A2	5.818008	12.41413	1.093386	8.80E-06	2.72E-05
CCDC196	0.531642	0.240874	-1.14218	8.29E-10	6.21E-09
ULBP1	0.057913	0.190243	1.71587	5.11E-06	1.66E-05
HHLA2	0.067014	0.144486	1.108406	3.22E-09	2.10E-08
ARHGAP28	0.070111	0.206656	1.559526	8.04E-06	2.51E-05
TKTL1	0.08908	1.871257	4.39277	7.61E-05	0.00019
QPCT	0.808236	2.306595	1.512916	6.00E-11	5.77E-10
ETV3L	0.004936	0.011463	1.215616	7.46E-06	2.35E-05
FAM153A	0.022783	0.06188	1.441537	0.005875	0.009715
H1-3	0.032187	0.073999	1.201021	1.08E-07	5.05E-07
PIF1	0.344102	1.026769	1.577206	6.29E-26	2.97E-23
MIR181A2HG	0.137993	0.380066	1.461656	6.05E-09	3.72E-08
HAVCR1	0.417039	1.288454	1.627388	2.24E-10	1.88E-09
OACYLP	0.015468	0.038589	1.31885	7.58E-05	0.00019
GPD1L	1.227393	2.597797	1.081692	7.60E-13	1.14E-11
RAD51	0.650118	1.759891	1.436713	1.76E-24	4.53E-22
ZNF878	0.052053	0.109883	1.077915	4.08E-12	5.13E-11
MUSTN1	0.146679	0.474463	1.693633	0.01061	0.016566
CDCA8	1.872059	5.9749	1.674288	2.32E-28	4.70E-25
CCDC192	0.046684	0.142253	1.607468	1.15E-09	8.33E-09
PDIA2	0.663442	2.683486	2.016065	2.33E-12	3.08E-11
CCNB1	4.927465	13.93196	1.49948	1.94E-25	7.72E-23
ACER1	0.109112	0.02822	-1.951	0.001703	0.003194
GRIK2	0.049562	0.146506	1.563642	1.12E-06	4.19E-06
IL11	0.051303	0.938312	4.192941	5.77E-05	0.000148
LINC01600	0.009055	0.026869	1.569112	3.01E-05	8.20E-05
MAGEB2	0.255662	4.24067	4.05198	2.13E-12	2.84E-11
LINC01436	0.916884	2.486478	1.439293	1.77E-05	5.12E-05
MXRA8	3.117145	6.515407	1.06363	0.012643	0.019365
COLEC12	0.43798	1.002855	1.195175	2.47E-05	6.90E-05
ABCC1	1.586506	3.211269	1.01729	1.42E-07	6.48E-07
RACGAP1	2.270298	5.188694	1.192489	7.80E-21	7.14E-19
FIGN	0.299012	0.698806	1.224689	5.25E-16	1.61E-14
LINC02404	0.280806	1.090862	1.957824	2.11E-08	1.15E-07
LINC02405	0.116305	0.264148	1.183437	4.06E-07	1.68E-06
MRPL40P1	0.076539	0.153425	1.003275	9.05E-06	2.79E-05
FAM135A-AS1	0.036064	0.086602	1.263831	9.59E-06	2.94E-05
LINC01697	0.063961	0.220727	1.786993	6.02E-07	2.40E-06
ZNF578	0.015109	0.038097	1.33425	3.82E-07	1.59E-06
UBE2SP1	0.231759	0.571268	1.301544	2.71E-17	1.14E-15

FADS1	7.793605	16.65894	1.095934	2.26E-06	7.96E-06
LEFTY2	0.013079	0.11516	3.138263	0.001059	0.00207
ESR1	0.797064	0.320873	-1.3127	6.11E-09	3.75E-08
LINC00595	0.009845	0.039441	2.002268	0.000104	0.000252
SPACA6	0.202453	0.538913	1.412466	6.47E-06	2.06E-05
CACNA1G	0.009096	0.022665	1.317102	1.05E-06	3.98E-06
CD1A	0.12616	0.257404	1.028787	4.70E-05	0.000123
RBL1	0.651525	1.449425	1.153589	6.15E-23	9.32E-21
DBN1	4.107448	8.287722	1.012733	5.83E-09	3.60E-08
CD109	0.946544	2.154177	1.186396	8.43E-08	4.05E-07
MSC-AS1	0.401839	0.845882	1.07384	0.002429	0.004402
LRRC37A6P	0.093055	0.198524	1.093157	6.45E-07	2.55E-06
WDR87	0.002312	0.010395	2.168426	4.59E-08	2.34E-07
PRR19	0.442665	0.891562	1.01012	6.99E-16	2.09E-14
CHRNA1	0.035945	0.663226	4.205627	8.07E-06	2.52E-05
IHO1	0.024558	0.068954	1.489437	0.00201	0.003704
PES1P1	0.040919	0.139621	1.770663	5.70E-08	2.84E-07
SLC29A4	2.982085	6.673381	1.162097	1.20E-05	3.61E-05
FCER1G	14.37871	31.38289	1.126044	8.08E-06	2.52E-05
ARID3A	1.470882	4.199683	1.513599	4.52E-13	7.11E-12
HRK	0.005462	0.071515	3.710712	8.98E-07	3.44E-06
TUBA3E	0.095378	0.201914	1.08201	0.000106	0.000258
SLC26A9	0.064777	0.864213	3.737835	0.000541	0.001125
FOLR1	0.746217	1.495912	1.003357	1.26E-05	3.76E-05
LINC02082	0.069212	0.187782	1.439968	7.79E-08	3.77E-07
MEG9	0.071155	0.277531	1.963617	0.000106	0.000258
HOXA10	0.496059	1.048679	1.07999	2.00E-06	7.12E-06
DZIP1L	0.201585	0.486339	1.270573	8.23E-11	7.71E-10
IGHGP	5.312027	11.83885	1.156195	0.003952	0.006808
HNRNPA1P8	0.072399	0.164368	1.182885	5.78E-10	4.45E-09
MNS1	1.355194	3.445814	1.346345	3.32E-05	8.96E-05
CAGE1	0.01362	0.078079	2.519245	2.99E-06	1.02E-05
C12orf42	0.013894	0.032738	1.236539	9.84E-10	7.21E-09
LINC01829	0.011062	0.024677	1.157493	0.000374	0.000807
ZNF300P1	0.199029	0.527523	1.406258	2.43E-08	1.31E-07
RPS20P22	0.015641	0.040392	1.368693	0.005741	0.009517
CPEB1	0.020999	0.136156	2.69685	0.005751	0.009532
DIRC3	0.020713	0.061423	1.568244	3.53E-07	1.48E-06
RAB39B	0.064395	0.128886	1.001065	1.01E-05	3.10E-05
RGS17	0.036283	0.101961	1.490658	9.22E-09	5.44E-08
ERVH48-1	0.100095	0.309626	1.62916	0.007597	0.012248
ZNF716	0.046969	0.127024	1.435318	2.14E-07	9.43E-07
LINC02466	0.01726	0.072465	2.069808	2.13E-10	1.80E-09
GRAMD4P8	0.015817	0.059239	1.905036	0.003024	0.005357

GGT2	0.016047	0.036161	1.172157	0.000856	0.001705
ZNF300	0.386782	0.938267	1.278479	3.17E-13	5.15E-12
EZH2	1.791049	4.10796	1.197617	3.49E-25	1.11E-22
TMEM108	0.045246	0.098773	1.126335	0.000504	0.001056
AMPD1	0.018463	0.037863	1.036147	0.000473	0.000995
DSC3	0.022274	0.166387	2.901111	0.015323	0.023028
PTN	0.827312	3.652168	2.14225	0.000365	0.000788
ALG14-AS1	0.0229	0.046907	1.034432	0.018429	0.027208
PHF19	1.165285	2.806303	1.267987	1.44E-24	3.86E-22
ACSL6	1.306487	0.649815	-1.00759	0.001147	0.002225
HMSD	0.05807	0.154927	1.415735	0.00034	0.000738
PDE6A	0.009607	0.026166	1.445453	3.45E-09	2.23E-08
LINC01807	0.10628	0.481725	2.180338	1.69E-06	6.09E-06
SAA2-SAA4	61.68478	16.90886	-1.86713	2.63E-06	9.13E-06
HPCAL4	0.008548	0.017877	1.064526	0.001404	0.002682
SMC2-DT	0.009532	0.019218	1.011647	9.43E-10	6.95E-09
EGFL6	0.089832	0.23495	1.387054	8.76E-07	3.37E-06
CHRNA5	0.090517	0.181053	1.000147	0.004617	0.007826
DRAXIN	0.022131	0.054258	1.2938	2.33E-05	6.53E-05
AARD	0.010613	0.035139	1.727305	9.26E-05	0.000227
C10orf55	0.026627	0.077228	1.536265	6.16E-05	0.000158
LINC00624	0.141615	0.30375	1.100912	0.000676	0.001378
SNHG25	3.076643	7.005266	1.187083	2.87E-06	9.84E-06
FAM72C	0.064861	0.176578	1.444888	1.20E-15	3.43E-14
BRCA2	0.181047	0.391545	1.11281	1.37E-17	6.18E-16
PTPRQ	0.014376	0.043023	1.581449	4.55E-06	1.50E-05
S100A8	1.821661	9.354641	2.360428	0.00388	0.006697
HCN4	0.03565	0.083412	1.226338	0.00116	0.00225
LINC01446	0.107115	0.281759	1.395297	1.94E-06	6.92E-06
TCP10L3	0.011134	0.033145	1.57383	0.002016	0.003714
BFSP2	0.092526	0.187778	1.021096	3.87E-07	1.61E-06
CCNA1	0.009562	0.043996	2.201955	0.00776	0.012491
PLXDC1	0.589899	1.396566	1.243343	9.14E-07	3.50E-06
LINC02204	0.011006	0.024349	1.145655	0.000351	0.000761
IGSF1	0.53756	2.337277	2.120331	3.88E-07	1.61E-06
ZNF665	0.061604	0.131045	1.088957	3.92E-10	3.12E-09
EGLN3	0.767802	2.407211	1.648556	6.12E-12	7.42E-11
DNM1	0.582698	1.284321	1.140185	1.05E-05	3.19E-05
UBE2U	0.105345	0.227619	1.111495	0.000684	0.001393
SRRM4	0.002875	0.009537	1.730049	4.63E-05	0.000122
LEFTY1	0.324134	1.574435	2.28017	3.68E-05	9.83E-05
PACSIN1	0.324665	0.876033	1.432031	1.23E-09	8.85E-09
GLYATL1	18.81646	9.001698	-1.06373	9.47E-14	1.75E-12
SOCS3-DT	0.164076	0.441322	1.427472	2.40E-08	1.30E-07

LGALS17A	0.064126	0.156114	1.283622	2.28E-07	9.97E-07
CSAG2	0.228801	0.813649	1.830314	9.19E-11	8.51E-10
HOXC6	0.097378	0.258513	1.40856	0.000233	0.000524
C18orf54	0.141888	0.414438	1.546399	1.43E-22	1.95E-20
TTYH1	0.206117	1.077199	2.385746	0.000198	0.000452
TTLL6	0.048331	0.13515	1.483534	0.000276	0.000611
EIF3KP1	0.02081	0.052858	1.344817	0.00108	0.002106
NEK2	1.63755	4.271804	1.383307	8.20E-20	6.14E-18
NRSN2	2.737536	7.674791	1.48725	2.25E-13	3.81E-12
H19	141.2781	327.0696	1.21106	4.62E-06	1.52E-05
KCNMB2-AS1	0.32657	1.156147	1.823859	2.90E-13	4.75E-12
FEZF1	0.047567	0.176093	1.888314	0.011241	0.017427
DDX43	0.10865	0.252113	1.214383	0.003904	0.006733
OOEP	0.097458	0.23174	1.24966	0.000346	0.00075
LINC00844	28.68659	14.00782	-1.03414	6.34E-07	2.51E-06
BDKRB1	0.196688	0.49937	1.3442	5.48E-06	1.78E-05
CELF3	0.007904	0.037945	2.263327	0.000551	0.001145
HCAR1	0.024059	0.103906	2.110651	0.00043	0.000913
HSPB7	0.215137	0.697831	1.697622	0.013458	0.020489
PEX5L	0.008205	0.017074	1.05714	1.87E-06	6.70E-06
BICC1	2.578241	5.270351	1.031512	4.91E-06	1.60E-05
IGKC	87.94485	287.2116	1.707443	0.008413	0.013435
LRFN5	0.039817	0.16277	2.03139	5.99E-07	2.39E-06
HP	1759.58	794.2995	-1.14748	3.09E-13	5.03E-12
CCDC162P	0.300356	0.712138	1.245485	6.45E-07	2.55E-06
FREM1	0.031655	0.289801	3.194544	1.05E-07	4.93E-07
ZNF215	0.087498	0.263601	1.591026	2.81E-09	1.86E-08
ZEB2P1	0.020985	0.094793	2.175389	8.38E-08	4.03E-07
KIF7	0.44653	0.920625	1.043858	1.34E-09	9.57E-09
MMP10	0.391524	1.447065	1.885955	5.69E-10	4.39E-09
HMGB2	10.92467	24.42118	1.160544	1.31E-22	1.83E-20
SPRY4-AS1	0.042096	0.096275	1.19348	1.10E-06	4.14E-06
TENT5B	0.138269	0.392496	1.505194	7.72E-09	4.65E-08
RPL32P20	0.184722	0.593653	1.684267	1.20E-07	5.55E-07
TAT	203.0686	100.054	-1.02119	3.49E-11	3.58E-10
TRPC1	0.25865	0.566058	1.129952	6.14E-13	9.42E-12
HES7	0.011794	0.026888	1.188876	3.10E-05	8.43E-05
ARMC3	0.004592	0.021494	2.226638	8.74E-11	8.13E-10
STMN1	7.572562	19.46906	1.36233	4.08E-26	2.20E-23
KLRG2	0.007636	0.017989	1.236194	5.67E-05	0.000146
PRSS3	4.027039	9.985383	1.310099	0.003289	0.005773
ZP1	0.013773	0.042193	1.61518	0.016232	0.024282
PRKG1-AS1	0.013443	0.04216	1.649017	7.32E-06	2.31E-05
VSIG8	0.014385	0.050489	1.811392	0.00065	0.001329

NDRG4	0.267512	0.989082	1.886484	0.012135	0.01866
GPR173	0.069846	0.149223	1.095214	0.020479	0.029923
RNF126P1	0.103342	0.215886	1.062845	0.000209	0.000475
LINC01980	0.702013	2.840771	2.016713	1.21E-11	1.37E-10
HPSE	0.336287	0.673105	1.001139	1.40E-05	4.13E-05
SSTR5-AS1	0.972681	1.9761	1.022617	0.012378	0.018993
CASC15	0.066854	0.17685	1.403439	3.77E-06	1.26E-05
CHRND	0.068342	0.278842	2.028597	0.014189	0.021501
VLDLR-AS1	0.025865	0.081352	1.65317	0.019235	0.02828
CASC20	0.05425	0.351362	2.695261	3.22E-14	6.61E-13
COL4A5	0.667076	1.452742	1.122855	1.83E-06	6.57E-06
ANKRD7	0.008566	0.07222	3.075703	3.31E-07	1.40E-06
LINC01205	0.009074	0.042992	2.244205	1.68E-09	1.18E-08
MMP3	0.089839	0.338194	1.91244	0.004908	0.008262
LINC00648	0.055713	0.369017	2.727605	3.76E-11	3.82E-10
HOXD10	0.093355	0.282041	1.595097	8.54E-05	0.000211
ZNF486	0.170605	0.690261	2.016486	2.89E-10	2.37E-09
SLC9A7P1	0.075701	0.201798	1.414527	1.76E-07	7.86E-07
KCNK9	0.110649	0.514999	2.218577	1.69E-13	2.96E-12
CYP8B1	92.707	44.05335	-1.07343	1.78E-09	1.23E-08
CDT1	2.07297	6.17663	1.575121	1.91E-26	1.34E-23
OPN1SW	0.938528	1.924978	1.03637	7.35E-15	1.76E-13
CD8A	1.295663	2.732669	1.076621	0.006505	0.010643
GRM1	0.004667	0.022234	2.252061	0.00624	0.010255
FCGR1CP	0.086785	0.224946	1.37407	6.09E-06	1.96E-05
PLA2G7	2.483998	5.620634	1.178069	0.000103	0.00025
CADM3	0.094865	0.208896	1.138832	0.000678	0.001381
PF4V1	0.431276	1.496258	1.794676	0.016594	0.024767
RHBDL2	0.130229	0.322856	1.309842	5.46E-10	4.22E-09
CDKL2	0.006591	0.018224	1.467291	0.000438	0.000929
FAM228A	0.035562	0.104153	1.550315	0.005491	0.009142
CSMD3	0.002543	0.019147	2.912737	7.74E-10	5.82E-09
KLHL30-AS1	0.005474	0.016548	1.595823	0.00076	0.00153
ENPP3	0.88587	2.196981	1.310356	6.51E-05	0.000166
LINC02476	0.301291	0.725721	1.268258	3.25E-06	1.10E-05
TMEM63C	0.077097	0.234451	1.604533	6.12E-06	1.96E-05
RAB34	4.229113	9.14145	1.112068	0.000112	0.000269
RNF17	0.017774	0.223896	3.655028	3.09E-11	3.23E-10
SSTR2	0.442757	1.203288	1.442395	3.40E-08	1.78E-07
ADH1B	302.7286	136.359	-1.15062	3.22E-12	4.12E-11
TFF1	1.639293	9.949217	2.601509	9.38E-06	2.89E-05
EYA4	0.068365	0.142226	1.056856	1.79E-08	9.94E-08
PIRT	0.003463	0.009167	1.404204	0.000598	0.001233
CCNB2	2.153015	5.973601	1.472242	6.97E-23	1.04E-20

FANCD2	0.549754	1.481935	1.430623	2.23E-25	8.73E-23
FOXC2	0.167204	0.43471	1.378443	0.000488	0.001025
GPSM2	0.429954	0.997605	1.214287	1.63E-20	1.37E-18
CSAG3	0.50511	1.467421	1.538613	1.43E-11	1.59E-10
L1CAM	0.037413	0.175572	2.230461	0.003194	0.005624
ZNF732	0.023049	0.079374	1.783941	5.97E-10	4.58E-09
TMEM45A	4.117864	8.39981	1.02846	0.000225	0.000507
TRPA1	0.013319	0.082389	2.629017	0.000483	0.001015
COL20A1	0.002021	0.005889	1.542898	0.002337	0.004246
KIR2DL4	0.07408	0.166758	1.170602	0.004924	0.008285
ZFHX4-AS1	0.09171	0.331462	1.853701	0.000258	0.000574
MACIR	0.724578	1.570772	1.11626	3.66E-10	2.93E-09
INS-IGF2	0.372752	0.051795	-2.84734	1.27E-05	3.78E-05
NPFFR2	0.156616	1.149369	2.87554	1.94E-11	2.10E-10
CYCSP24	0.047469	0.104724	1.141531	1.31E-06	4.85E-06
RUNDC3A-AS1	0.010532	0.024462	1.215727	7.85E-08	3.80E-07
PTGES3L-AARSD1	0.005747	0.018221	1.664691	2.19E-07	9.62E-07
SLC8A1-AS1	0.001924	0.008509	2.145202	1.02E-06	3.88E-06
ESPL1	0.821961	1.907449	1.214502	1.76E-18	9.72E-17
NCAM1	0.223314	0.494211	1.146054	0.010733	0.016725
KIF3C	0.462144	1.099561	1.250513	4.50E-06	1.48E-05
IL31RA	0.015129	0.084462	2.480948	6.08E-07	2.42E-06
AACSP1	0.008647	0.196719	4.507787	1.78E-12	2.42E-11
COLCA2	1.007173	2.182475	1.115654	3.34E-12	4.26E-11
B3GALNT1	0.454081	1.054603	1.215679	6.83E-11	6.49E-10
EREG	0.185491	0.569435	1.618182	4.47E-05	0.000118
CARD18	0.059998	0.277486	2.209437	8.38E-10	6.27E-09
RPL10P6	3.858901	11.56857	1.583949	0.009477	0.014969
FLNC	1.71421	3.877821	1.177702	0.001395	0.002666
PWWP3B	0.296377	0.641266	1.113489	2.21E-06	7.79E-06
DDX11	0.992181	2.092903	1.07683	6.57E-19	4.15E-17
TOMM40P2	0.10155	0.22206	1.128758	2.96E-10	2.42E-09
TXNDC2	0.003432	0.007697	1.165347	0.000897	0.001778
IGHV3-35	0.088001	0.26485	1.589584	0.000796	0.001594
SNHG14	0.425912	0.856246	1.007471	1.10E-06	4.14E-06
BLM	0.358426	0.798219	1.15511	2.91E-19	2.00E-17
GOLM2P1	0.010204	0.036523	1.839689	2.07E-05	5.88E-05
TPRXL	0.037778	0.160048	2.082876	4.78E-09	3.01E-08
CKMT1A	0.074912	0.194018	1.372922	1.01E-10	9.26E-10
SLC36A2	0.004091	0.031047	2.924032	2.00E-07	8.88E-07
ARHGAP28-AS1	0.026134	0.13667	2.386701	2.08E-07	9.19E-07
CNGB3	0.011042	0.041924	1.924712	2.26E-08	1.23E-07

NRG3	0.081483	0.197872	1.279992	9.34E-06	2.88E-05
UBE2E1-AS1	0.015808	0.043112	1.44746	2.82E-05	7.75E-05
MYBPHL	0.167912	0.55616	1.727793	0.001581	0.002984
UTS2B	0.059278	0.291763	2.299226	1.52E-06	5.54E-06
SLC5A5	0.0325	0.103075	1.665193	0.003632	0.006315
MAP3K15	0.034914	0.119567	1.775922	8.63E-09	5.13E-08
INSRR	0.02648	0.071101	1.424948	0.0097	0.015279
HERC2P4	0.016553	0.044659	1.431832	3.49E-08	1.82E-07
KBTBD12	0.016028	0.068606	2.097748	0.000428	0.000909
ADAMTS16-DT	0.458141	1.130567	1.303182	0.009871	0.015514
CYP4F29P	0.032939	0.19979	2.600616	0.018675	0.027531
TMPOP2	0.049905	0.116116	1.218299	1.74E-12	2.37E-11
LINC01587	0.129056	0.623183	2.271655	2.20E-07	9.64E-07
LINC02345	0.023048	0.061714	1.420928	1.91E-10	1.63E-09
C7orf57	0.007622	0.018708	1.295312	0.000513	0.001071
LINC00348	0.270629	0.760347	1.490339	1.03E-05	3.13E-05
CLEC5A	0.102563	0.235911	1.201735	4.83E-08	2.45E-07
HAP1	0.054454	0.145303	1.415954	0.002463	0.004456
NUSAP1	5.281404	12.15823	1.20294	3.92E-19	2.62E-17
RBPJL	0.010432	2.408965	7.851256	0.007338	0.011882
HENMT1	0.688775	1.622289	1.235925	7.57E-05	0.000189
PMAIP1	0.364009	0.757974	1.058174	6.08E-07	2.42E-06
CIDEC	1.377566	3.483508	1.33842	3.66E-06	1.23E-05
EID3	0.272347	0.583822	1.100084	1.19E-07	5.53E-07
GPR158	0.305574	0.615332	1.009844	3.78E-09	2.43E-08
ACP4	0.194381	0.603273	1.633925	1.00E-10	9.22E-10
ST18	0.004796	0.044071	3.200017	2.76E-05	7.60E-05
TRPM6	0.021119	0.062512	1.565621	0.00325	0.005709
JSRP1	0.263437	0.695801	1.40122	2.66E-06	9.21E-06
CAMKV	0.019773	0.103359	2.386023	7.36E-07	2.88E-06
ARHGAP31-AS1	0.049251	0.148703	1.594213	4.36E-07	1.80E-06
IL12A	0.06117	0.198546	1.698571	1.07E-14	2.47E-13
BPIFA2	0.177426	0.565339	1.671896	4.54E-08	2.32E-07
CLRN1-AS1	0.054395	0.023464	-1.21304	0.000284	0.000627
GINS4	0.356702	0.855408	1.261894	2.66E-14	5.63E-13
LINC00205	0.899505	1.916102	1.090971	2.05E-17	8.96E-16
FCGR3B	0.17593	1.33374	2.922404	0.010957	0.017035
FER1L5	0.01383	0.053741	1.958179	2.06E-13	3.53E-12
CKMT1B	0.074133	0.429691	2.535114	2.11E-08	1.15E-07
LINC00942	0.645105	2.496149	1.952099	7.86E-05	0.000196
ELOBP2	0.08849	0.376663	2.08968	0.001795	0.00335
SNAP25-AS1	0.284536	0.601557	1.080093	0.001487	0.002824
PMPCAP1	0.020235	0.12345	2.608998	1.32E-08	7.56E-08

SYNPO2L	0.008516	0.031276	1.876874	0.026241	0.037395
DKKL1	0.142793	0.297472	1.05883	5.13E-05	0.000133
P2RY6	0.400888	0.973188	1.279517	2.67E-05	7.39E-05
ZNF85	0.185384	0.469036	1.339186	2.92E-15	7.57E-14
PCDHB1	0.003526	0.012875	1.868593	0.004668	0.007902
DDN-AS1	0.049959	0.109588	1.133284	1.97E-11	2.13E-10
NKAIN4	0.01674	0.152045	3.183148	2.70E-07	1.16E-06
GALNT14	0.197241	0.449919	1.189702	0.019348	0.028411
MLANA	0.102618	2.028277	4.304905	0.009084	0.014407
ENTPD3	0.064806	0.239694	1.886989	0.000639	0.00131
TPX2	4.620189	13.80975	1.579663	5.12E-26	2.53E-23
HOXC10	0.229885	0.920039	2.000782	0.002112	0.003871
KIF11	1.047717	2.865405	1.451489	5.64E-23	8.61E-21
CPA6	0.151555	0.623705	2.041023	0.006526	0.01067
BIRC5	3.333392	10.7089	1.683748	8.92E-25	2.55E-22
PNMA5	0.289837	2.252908	2.958476	3.64E-07	1.53E-06
SCG2	0.095774	0.230477	1.266915	0.000342	0.000742
TEX41	0.089696	0.235217	1.390879	9.14E-09	5.40E-08
CSMD1	0.068744	0.244961	1.833248	1.60E-08	8.96E-08
BARD1	0.455342	0.959537	1.075388	1.72E-18	9.49E-17
C4orf46	0.705711	1.429821	1.018685	3.71E-22	4.59E-20
ZWINT	4.45081	11.36304	1.352209	9.01E-26	3.98E-23
NIPAL4	0.009534	0.024758	1.376787	0.000302	0.000661
KIF24	0.264453	0.583383	1.141431	2.24E-15	6.01E-14
DDX43P3	0.109108	0.538495	2.303174	9.57E-08	4.54E-07
RBX1P2	0.168324	0.381643	1.180987	0.000502	0.001051
SNORD72	0.194882	0.4401	1.175234	1.32E-05	3.93E-05
VN1R81P	0.17001	0.452989	1.413858	3.78E-08	1.96E-07
VN1R51P	0.035725	0.096497	1.433564	0.001462	0.002783
CCNJL	0.156394	0.517629	1.726733	1.69E-05	4.90E-05
IGF2BP3	0.167577	0.895431	2.417754	1.01E-15	2.94E-14
E2F1	5.372554	12.76413	1.248416	1.17E-21	1.28E-19
FAM182A	0.004225	0.029567	2.806814	5.07E-11	4.98E-10
WDR49	0.005412	0.018006	1.734232	2.06E-05	5.84E-05
GNB3	0.039517	0.080295	1.022863	1.49E-09	1.05E-08
MAFG-DT	0.961791	1.971767	1.035694	2.38E-10	1.98E-09
KLHL14	0.008684	0.041633	2.261255	0.020448	0.029881
ZIM2-AS1	0.032656	0.104679	1.680547	4.48E-06	1.47E-05
EGFEM1P	0.023869	0.084746	1.827993	1.22E-07	5.65E-07
FSIP2-AS2	0.038852	0.131395	1.757862	3.21E-08	1.69E-07
ERC2	0.014693	0.053293	1.858834	1.42E-11	1.59E-10
HLA-DPA3	0.134121	0.275078	1.036307	0.007318	0.011851
MFFP2	0.017425	0.051055	1.550888	5.71E-09	3.53E-08
STAP1	0.109805	0.23655	1.1072	0.004867	0.008201

CLDN18	0.112655	0.4058	1.848855	4.13E-10	3.26E-09
IL20RA	0.172551	0.517315	1.584022	1.31E-07	6.04E-07
RBP2	0.224457	2.62847	3.549715	5.04E-06	1.65E-05
GPAT2P1	0.009142	0.068453	2.904596	0.000351	0.00076
NMB	3.066128	6.917873	1.173911	3.48E-10	2.80E-09
IGLV1-50	0.08958	0.22103	1.302996	0.033054	0.046069
IGLV1-44	8.357566	27.23784	1.704457	0.023532	0.033886
CDC20B	0.066177	0.202695	1.614907	4.11E-06	1.37E-05
IFNG	0.11904	0.331021	1.475475	5.68E-05	0.000146
PNMA3	0.5686	2.95935	2.379793	1.31E-06	4.85E-06
GJA3	0.015841	0.094515	2.576842	2.56E-08	1.37E-07
P2RY4	0.025499	0.11184	2.132936	0.000221	0.000499
SPATA46	0.265435	0.102676	-1.37026	0.002968	0.005269
PCLAF	1.575291	3.851484	1.289797	2.62E-21	2.63E-19
TP73	0.338746	0.715016	1.077773	2.30E-06	8.06E-06
LINC02889	0.441844	1.370758	1.633366	0.003915	0.006752
ULBP2	0.305767	1.021707	1.740479	2.48E-08	1.34E-07
TNFAIP6	0.417484	0.861035	1.044349	9.81E-09	5.75E-08
SLITRK2	0.003127	0.022906	2.872811	6.83E-06	2.17E-05
ZFP82	0.174189	0.407114	1.224781	1.94E-14	4.27E-13
ILDR2	0.360056	0.78749	1.129038	0.000793	0.001588
IGHV1-18	9.58029	22.69965	1.244529	0.034133	0.047435
MCM10	0.352162	1.263881	1.843551	4.12E-26	2.20E-23
PI3	4.382709	8.961277	1.031881	0.008516	0.013582
COL2A1	1.979431	5.601415	1.500706	0.000197	0.00045
LINC02188	0.066102	0.318107	2.266738	0.000141	0.000333
TROAP	1.239114	3.963348	1.67741	1.89E-26	1.34E-23
ZNF793-AS1	0.29233	0.847471	1.535566	5.47E-08	2.74E-07
PRC1	2.245153	5.289028	1.236189	3.43E-20	2.74E-18
DLGAP5	0.956416	2.830536	1.565364	2.83E-23	4.80E-21
FEN1	8.426904	16.927	1.006252	5.58E-24	1.20E-21
NTNG1	0.00175	0.034915	4.318629	9.45E-06	2.91E-05
KCNK12	0.011577	0.053499	2.208297	5.15E-07	2.09E-06
LIN28B	0.084	1.031361	3.618019	2.69E-14	5.67E-13
TCN1	0.180949	0.925939	2.355334	8.85E-05	0.000218
UICLM	0.345956	0.959628	1.471885	0.01456	0.022001
VWA5B2	0.06132	0.17207	1.488562	4.62E-09	2.92E-08
GTSE1	0.704253	2.156318	1.614404	3.48E-23	5.60E-21
LINC02882	0.457154	1.262812	1.465888	1.15E-06	4.32E-06
NPTX2	1.924605	8.356925	2.11841	0.002593	0.004664
LINC01783	0.046774	0.111507	1.253345	0.001957	0.003617
HSPA7	0.934285	3.412777	1.869012	1.30E-07	6.00E-07
SCN5A	0.013951	0.039823	1.513257	1.19E-08	6.87E-08
KIF2C	1.305212	5.045023	1.950577	1.25E-29	6.33E-26

LCN15	0.010696	0.254529	4.572671	0.014244	0.021566
PLBD1	1.455434	5.446333	1.903836	1.15E-11	1.31E-10
POTEKP	0.007449	0.062167	3.060972	6.32E-10	4.83E-09
PRECSIT	0.279153	0.600932	1.106146	5.61E-10	4.33E-09
LRP4-AS1	0.033059	0.073497	1.15264	3.19E-08	1.68E-07
SPHK1	2.366896	8.41836	1.830543	5.85E-11	5.65E-10
MCEMP1	0.058029	0.162469	1.48532	4.87E-05	0.000127
GNAZ	2.118902	4.523918	1.094256	1.37E-10	1.22E-09
SERPINE2	2.087844	4.639641	1.152	1.15E-05	3.46E-05
CPVL	4.851436	10.84002	1.159884	3.11E-06	1.06E-05
OXCT1	0.844234	2.166815	1.359861	6.55E-08	3.22E-07
DLX6-AS1	0.018142	0.057786	1.671355	0.000439	0.000931
SPC24	2.556182	6.099338	1.254662	1.81E-21	1.91E-19
DLX5	0.107558	0.488662	2.183718	0.000113	0.000273
ZNF670-ZNF695	0.018023	0.039234	1.122256	1.58E-07	7.14E-07
ARHGEF2-AS2	0.068083	0.168972	1.311423	2.79E-15	7.30E-14
MUC15	0.163717	0.715919	2.128592	5.45E-10	4.22E-09
TDRD1	0.032955	0.136968	2.055269	0.004812	0.008119
ZAR1L	0.008416	0.020397	1.277214	1.74E-06	6.28E-06
DPF1	0.020775	0.072768	1.808429	1.03E-10	9.41E-10
PDCD6IPP1	0.0437	0.094505	1.112743	0.008424	0.013451
CASC9	1.177763	3.524424	1.581339	6.92E-12	8.28E-11
CYP3A43	1.958811	0.859112	-1.18906	7.21E-08	3.52E-07
ASNSP1	0.334525	0.698068	1.061252	5.16E-08	2.60E-07
TUSC3	1.407917	3.540557	1.330414	2.42E-05	6.77E-05
FMO1	1.06235	2.199892	1.050174	3.80E-05	0.000101
ZNF296	0.532095	1.193292	1.16519	5.37E-14	1.05E-12
COL11A1	0.132095	0.995471	2.913804	4.30E-06	1.42E-05
SMC4	1.574328	3.247601	1.044638	6.55E-12	7.88E-11
SCGB2A1	0.472143	1.154025	1.28938	0.002695	0.004831
CDH12	0.187894	0.564354	1.586679	6.65E-06	2.12E-05
PKMYT1	0.762908	1.93712	1.344333	1.48E-21	1.58E-19
TRIM67	0.033022	0.090579	1.455735	0.000282	0.000622
KIF14	0.439578	0.998496	1.183638	5.49E-15	1.35E-13
CKAP2L	0.570806	1.539845	1.431714	1.98E-20	1.64E-18
WDR45BP1	0.020206	0.040612	1.007099	0.004236	0.007247
ZNF354C	0.212904	0.440987	1.050534	3.56E-08	1.85E-07
TM4SF20	3.011901	6.422855	1.092541	0.000525	0.001095
S100A5	0.020643	0.051774	1.326572	0.000124	0.000297
CFHR3	37.0088	17.93328	-1.04523	1.72E-07	7.73E-07
STXBP5-AS1	0.043987	0.113816	1.371553	2.05E-08	1.12E-07
SAMMSON	0.006554	0.04301	2.714251	2.97E-14	6.19E-13
BMS1P22	0.010808	0.03364	1.63807	1.25E-09	8.96E-09

PFN2	2.408717	7.022686	1.543758	1.17E-06	4.38E-06
ACRV1	0.028748	0.090491	1.654325	1.01E-19	7.50E-18
KRT17	0.725904	3.335095	2.199877	1.87E-07	8.34E-07
FANCI	1.107628	2.553396	1.204944	4.47E-23	6.98E-21
TRIM36	0.086511	0.186183	1.105757	1.22E-06	4.54E-06
OR7E128P	0.042417	0.115177	1.441153	4.52E-07	1.85E-06
LINC01843	2.282909	4.618279	1.016482	1.31E-07	6.03E-07
EXO1	0.716247	1.872449	1.386397	1.37E-21	1.49E-19
DLGAP3	0.093305	0.187844	1.009516	5.75E-07	2.30E-06
TXLNB	0.106384	0.27228	1.355805	1.81E-06	6.50E-06
FAM133A	0.649629	1.656541	1.350486	5.02E-08	2.54E-07
HECW2-AS1	0.075909	0.232253	1.613348	0.006474	0.0106
UGT1A10	0.155061	0.957207	2.625996	3.41E-05	9.18E-05
TBC1D30	0.360993	0.73716	1.030006	4.10E-08	2.11E-07
LINC02086	0.018042	0.052433	1.539093	0.016406	0.024518
FOXD2-AS1	0.902924	2.264031	1.326217	1.08E-16	3.85E-15
DUX4L27	0.062867	0.14205	1.176031	0.009097	0.014427
ART5	0.076637	0.322046	2.071145	0.000585	0.001207
UGT2B28	0.090226	0.183449	1.023769	0.007298	0.01182
AKAIN1	0.121851	0.053155	-1.19684	0.014444	0.021838
KCNH7	0.019192	0.060193	1.649094	0.000153	0.000358
ZNF14	0.496791	1.126232	1.180792	8.13E-16	2.41E-14
CMKLR2	0.069102	0.176508	1.352937	0.007061	0.011464
IRGM	0.034742	0.077633	1.159968	0.000532	0.001108
UAP1L1	1.149927	2.737338	1.251231	2.09E-05	5.93E-05
GAPDHP33	0.018496	0.037156	1.006424	0.00131	0.002515
MELTF-AS1	0.581907	1.351619	1.215827	3.10E-14	6.39E-13
IGLC2	44.40334	136.6485	1.621729	0.026343	0.037524
AMIGO2	0.945789	1.942808	1.038552	4.65E-07	1.90E-06
KEL	0.22331	2.027776	3.182776	3.83E-05	0.000102
CCDC136	0.040075	0.106985	1.416641	3.09E-07	1.32E-06
SEZ6L	0.014445	0.232814	4.010501	0.018278	0.027016
TRIM54	0.387232	1.029792	1.411082	1.38E-07	6.33E-07
FAM225A	0.022156	0.063605	1.521411	2.12E-07	9.33E-07
PAQR5-DT	0.025419	0.052607	1.049332	1.25E-06	4.64E-06
LINC02055	0.218959	0.493828	1.17335	6.10E-05	0.000156
PURG	0.0069	0.018932	1.456149	0.012953	0.019796
NBEAP2	0.23697	0.634343	1.420557	1.98E-11	2.14E-10
FAM178B	0.082393	0.641238	2.960273	2.38E-11	2.54E-10
CHP2	0.364723	0.848969	1.218911	1.37E-05	4.06E-05
LMNB2	3.380833	7.747776	1.196403	5.34E-25	1.62E-22
ZNF492	0.017025	0.108586	2.673095	4.22E-13	6.68E-12
DDIT4L	0.275564	0.692603	1.329643	0.009747	0.015342
WASIR2	0.028303	0.088156	1.639102	3.95E-11	3.99E-10

TNFRSF13C	0.145198	0.292329	1.009567	1.02E-05	3.11E-05
MFAP2	0.500154	1.819096	1.862777	1.49E-08	8.43E-08
RGS1	3.179569	7.25605	1.190353	3.50E-08	1.83E-07
ZNF454	0.055787	0.135623	1.281607	1.74E-05	5.02E-05
SPOCD1	0.062715	0.187269	1.578222	2.62E-05	7.24E-05
HCAR3	0.066479	0.196107	1.560669	1.24E-05	3.70E-05
KLHL30	0.124229	0.465998	1.907323	0.003995	0.006877
ZNF560	0.046129	0.184096	1.996717	1.38E-10	1.22E-09
SEMA3A	0.069757	0.265135	1.926309	0.000495	0.001039
DLX6	0.067277	0.220924	1.715366	0.000806	0.001614
DOK6	0.092268	0.230341	1.319866	0.008879	0.014119
C1orf147	0.017028	0.034817	1.031864	1.59E-05	4.63E-05
CNTN5	0.017286	0.044377	1.360218	3.63E-07	1.52E-06
C17orf64	0.03432	0.086276	1.329911	0.000179	0.000413
SP140	0.300477	0.691426	1.20232	1.68E-08	9.37E-08
RN7SKP287	0.045032	0.113545	1.334229	7.12E-06	2.25E-05
FBXO43	0.221898	0.560633	1.337159	7.25E-18	3.46E-16
KIF5A	0.01616	0.077515	2.262061	2.73E-15	7.17E-14
PAGE2	2.849001	13.84797	2.281146	1.26E-09	9.02E-09
CRYBB1	0.297854	4.303712	3.852903	0.001808	0.003369
CNR1	0.056314	0.349352	2.633124	0.006301	0.010342
PPP1R1B	0.261868	1.373282	2.390719	3.53E-06	1.19E-05
RMI2	1.636345	3.92831	1.263432	1.24E-18	7.13E-17
NTMT2	0.017293	0.06931	2.002889	1.43E-12	2.00E-11
ARL4AP2	0.03579	0.093206	1.380879	5.18E-06	1.68E-05
RPL39L	4.19613	8.84413	1.075661	3.19E-07	1.35E-06
RTKN2	0.094098	0.315756	1.746576	3.25E-22	4.05E-20
KCNN1	0.067571	0.138267	1.032971	6.17E-05	0.000158
RNF186	0.115646	0.36726	1.667085	5.79E-06	1.86E-05
ASRGL1	0.945804	2.47971	1.390558	6.59E-13	1.00E-11
SNRPFP1	0.044587	0.092242	1.048788	1.19E-05	3.56E-05
SNORD36C	0.215595	0.452144	1.068456	1.52E-05	4.44E-05
RUNX2	0.134083	0.272646	1.023897	0.011807	0.018203
LETM2	0.085292	0.173893	1.027712	0.000394	0.000844
MIR589	0.242089	0.574204	1.246025	3.74E-10	2.99E-09
ZNF883	0.2173	0.811456	1.900824	5.17E-11	5.06E-10
LYPD6	0.120948	0.359116	1.570062	3.68E-09	2.37E-08
TCF19	3.626567	7.749868	1.095567	3.40E-18	1.76E-16
DLX4	0.066448	0.143495	1.110694	2.32E-08	1.26E-07
MTMR7	0.484475	1.216546	1.328295	2.27E-08	1.23E-07
TRAV20	0.072829	0.186026	1.352911	0.018914	0.027846
FMN2	0.011409	0.093834	3.039965	3.54E-07	1.49E-06
ZNF738	0.192807	0.624527	1.69561	2.44E-19	1.70E-17
DEFB132	1.098687	0.497712	-1.1424	5.29E-08	2.66E-07

LINC02334	0.022767	0.050079	1.137269	0.024915	0.035684
SLC1A2	7.107831	3.480432	-1.03014	6.47E-09	3.96E-08
RTL1	0.006148	0.723336	6.87839	2.83E-08	1.51E-07
ZNF826P	0.156388	0.582846	1.897985	2.40E-15	6.38E-14
TRIM59	0.188632	0.443189	1.232347	8.09E-19	4.99E-17
NOC2LP1	0.106474	0.427823	2.006518	8.55E-11	7.98E-10
BRCA1	0.633819	1.348724	1.089451	3.71E-18	1.91E-16
HUS1B	0.061202	0.142986	1.224227	3.04E-14	6.31E-13
KCNMB3	0.089702	0.226449	1.335976	2.27E-19	1.60E-17
POTEF	0.009013	0.0225	1.319868	6.20E-05	0.000158
GDF11	0.377759	0.859598	1.186197	9.51E-09	5.60E-08
LINC01615	0.077374	0.268618	1.795639	0.014233	0.021556
MAGEA3	2.021067	10.04601	2.313434	1.02E-10	9.36E-10
TNFRSF13B	0.042869	0.089458	1.061268	0.006277	0.010309
CHAF1B	0.774152	2.085078	1.429412	1.93E-24	4.88E-22
EIF3EP1	0.123279	0.282984	1.198796	2.96E-09	1.95E-08
CYP26A1	0.987201	0.315742	-1.6446	0.000474	0.000997
ASPM	1.192213	2.872754	1.268792	4.33E-17	1.69E-15
FOXO6-AS1	0.062051	0.197321	1.669022	1.64E-05	4.78E-05
LINC02714	0.01818	0.105811	2.541062	4.23E-08	2.17E-07
RNF183	0.046335	0.1577	1.767005	5.81E-06	1.87E-05
MMP11	2.444185	5.675789	1.215467	1.18E-10	1.06E-09
ANKRD13B	0.417855	0.912419	1.126695	1.35E-11	1.51E-10
ART3	0.052545	0.108074	1.040394	0.000473	0.000995
EDIL3-DT	0.030564	0.084459	1.466434	0.000686	0.001396
LINC02616	0.030105	0.21014	2.803264	9.93E-13	1.45E-11
ACOT11	0.093935	0.190847	1.022686	2.19E-10	1.84E-09
HPR	243.7297	121.1163	-1.00889	6.68E-12	8.01E-11
JMJD1C-AS1	0.123927	0.260961	1.074344	1.33E-12	1.88E-11
CCDC13	0.142336	1.164996	3.032957	0.012185	0.018727
FSTL5	0.141788	0.480868	1.761908	3.09E-09	2.03E-08
ARMCX2	0.691826	1.450066	1.067639	0.031407	0.043977
RPS27AP6	0.047958	0.103118	1.104442	4.60E-05	0.000121
TCP10L2	0.020866	0.050885	1.286109	0.001238	0.002389
GNAO1-DT	0.076988	0.035102	-1.13307	3.27E-05	8.83E-05
S100A9	17.69841	77.98703	2.139614	0.000407	0.000869
TRIM46	0.087504	0.207007	1.242268	9.35E-09	5.51E-08
ZNF98	0.008199	0.088144	3.426285	1.97E-10	1.68E-09
EFS	0.275084	0.643255	1.225521	0.010308	0.016129
LINC01529	0.047868	0.137223	1.519401	5.85E-08	2.91E-07
CHST11	1.1399	2.734446	1.262341	3.41E-09	2.21E-08
CCNI2	0.059858	0.141282	1.238953	1.89E-09	1.30E-08
HAGLR	0.81713	2.224636	1.444931	0.00076	0.00153
SKA3	0.799693	2.310542	1.530714	4.36E-24	9.51E-22

SMC1B	0.124734	0.485404	1.960331	6.11E-13	9.38E-12
MANCR	0.056619	0.222592	1.975048	0.014362	0.021721
ZNF804A	0.043258	0.116061	1.423829	0.001014	0.001988
ADGRE3	0.032704	0.102836	1.65281	0.032389	0.045247
HSPA8P14	0.006663	0.015567	1.224256	5.22E-06	1.69E-05
RHOXF1-AS1	0.127472	0.286104	1.166367	0.006626	0.01082
RIBC2	0.347541	1.263887	1.862612	4.32E-19	2.86E-17
ACKR4	0.069118	0.14693	1.088004	0.012969	0.019814
COL28A1	0.042647	0.212123	2.314371	4.48E-12	5.58E-11
ZNF280B	0.041977	0.093254	1.151581	0.00018	0.000415
LINC02327	0.019643	0.162021	3.044115	2.61E-09	1.74E-08
RAD54L	0.385703	1.413831	1.874048	1.88E-27	2.55E-24
HPSE2	0.010134	0.063352	2.644177	1.01E-06	3.84E-06
LINC01611	0.169587	0.50334	1.569505	5.74E-09	3.55E-08
EPHA7	0.024553	0.057759	1.234136	0.002271	0.004137
RPL10P9	6.07999	15.50389	1.35049	0.023149	0.033388
TRIM45	0.670274	1.490522	1.152995	1.36E-17	6.17E-16
RFX6	0.025204	0.133782	2.408179	3.53E-09	2.28E-08
CCL26	0.331223	0.908927	1.456359	3.60E-07	1.51E-06
LINC01686	0.080445	0.231962	1.527812	0.015156	0.022806
ZDBF2	0.173253	0.351834	1.022014	0.006505	0.010643
LINC-ROR	0.019177	0.092527	2.270465	5.32E-08	2.67E-07
H2AC14	0.040023	0.118938	1.57132	3.74E-09	2.40E-08
WDR76	1.107162	2.435759	1.137505	3.66E-19	2.46E-17
CDCA7L	1.047641	2.484685	1.245918	3.62E-13	5.81E-12
ACTG1P19	0.008828	0.018636	1.077851	1.79E-05	5.17E-05
MUC5B	1.983467	5.623558	1.503459	1.88E-05	5.39E-05
COL11A2	0.103977	1.035458	3.315933	0.00419	0.007179
TMEM18-DT	0.024779	0.072027	1.539441	0.00073	0.001477
SOBP	0.738988	1.579052	1.095436	6.48E-08	3.20E-07
SCUBE3	0.053033	0.148458	1.485096	0.000109	0.000263
LINC01281	0.013229	0.036647	1.469971	0.000422	0.000897
CDCA3	0.763452	2.203744	1.529347	5.56E-25	1.66E-22
TRIM60P17	0.037545	0.095335	1.344382	1.92E-06	6.88E-06
NEIL3	0.356206	1.095959	1.621409	1.72E-18	9.49E-17
EPHB3	0.307855	1.130836	1.877067	8.76E-06	2.71E-05
MYLK2	0.03016	0.112954	1.905024	2.29E-17	9.84E-16
TCEAL2	0.026966	0.356221	3.723541	0.021237	0.030914
SUN3	0.036977	0.139987	1.920571	3.42E-07	1.44E-06
ZNF816	0.470447	0.991039	1.07491	2.06E-14	4.48E-13
TRIM72	0.046414	0.117258	1.337051	2.88E-07	1.23E-06
TUBB2B	0.875664	1.875655	1.098944	3.60E-05	9.64E-05
CDIPTOSP	0.129099	0.263726	1.030565	6.60E-07	2.60E-06
LINC02562	0.078398	0.312339	1.994218	2.00E-11	2.16E-10

CTLA4	0.366336	0.820251	1.162896	1.71E-07	7.69E-07
NOG	0.016518	0.038229	1.210585	0.021784	0.031635
PLAC4	0.019563	0.389768	4.316449	0.032722	0.045659
OR51B5	0.020044	0.102849	2.35932	0.003013	0.005341
DPY19L2P1	0.0041	0.016601	2.017639	0.009496	0.014998
ZBED9-AS1	0.007249	0.024471	1.755234	1.50E-08	8.47E-08
CYP1A1	31.20192	5.55777	-2.48906	0.001594	0.003008
CTSV	0.67297	2.153058	1.677774	2.65E-11	2.80E-10
CENPU	2.264289	4.958617	1.13088	2.07E-17	9.01E-16
ZSCAN5B	0.013465	0.080782	2.584842	0.000193	0.000442
HMMR	1.481776	3.485241	1.233931	8.51E-18	4.03E-16
SNHG28	0.086962	0.18589	1.095984	0.000221	0.0005
VIPR2	0.017785	0.133336	2.906304	3.35E-06	1.13E-05
POU5F2	0.002508	0.005086	1.019862	2.60E-06	9.04E-06
TDRD5	0.027034	0.209337	2.952972	3.12E-09	2.04E-08
BOLA3-AS1	0.146322	0.319637	1.127289	1.65E-06	5.97E-06
TMC6	1.836174	3.780285	1.041792	4.16E-07	1.72E-06
CABYR	1.378687	2.90054	1.073026	0.002517	0.004541
LINC02335	0.142839	0.450047	1.655686	3.41E-08	1.79E-07
ZIC5	0.277559	0.778401	1.487721	7.31E-10	5.52E-09
LMX1A	0.015383	0.08148	2.405118	1.69E-05	4.89E-05
PCDH10	0.002801	0.015666	2.483602	0.000487	0.001022
MCM6	5.115675	11.39958	1.155985	2.24E-24	5.55E-22
LINC02864	0.132501	0.632817	2.255785	1.29E-13	2.31E-12
IL12A-AS1	0.005313	0.020057	1.916659	4.35E-08	2.23E-07
ZNF534	0.01235	0.084957	2.782205	8.34E-09	4.98E-08
UBE2T	5.594391	12.54355	1.164893	3.74E-21	3.65E-19
MIXL1	0.025758	0.112164	2.122543	1.58E-08	8.88E-08
ASIC4	0.00666	0.020977	1.655127	2.91E-08	1.55E-07
BAGE2	0.036857	0.207916	2.495997	3.41E-08	1.79E-07
GLYAT	29.73382	12.86168	-1.20903	4.76E-12	5.89E-11
PGAM1P7	0.030014	0.069506	1.211501	0.0001	0.000245
GSTT2B	1.820556	3.924165	1.108006	0.001605	0.003026
TRIM60P18	0.4615	0.93216	1.014247	3.67E-13	5.88E-12
ARHGAP33	0.810401	1.644841	1.02124	1.52E-17	6.81E-16
TRBV4-1	0.184878	0.398175	1.106826	0.002302	0.004188
STK31	0.03105	0.111297	1.841778	4.63E-07	1.89E-06
HILPDA	1.79479	4.867878	1.439478	2.59E-17	1.10E-15
SENP3-EIF4A1	0.007525	0.015185	1.012999	5.33E-06	1.73E-05
CHST6	0.021057	0.049103	1.22151	8.30E-05	0.000206
VCX	0.091005	0.577035	2.664636	2.26E-12	3.00E-11
LINC01322	0.054539	0.128566	1.237157	5.32E-05	0.000138
FCN3	5.465571	2.702823	-1.01591	0.013459	0.020489
FCRLA	0.167728	1.038829	2.630765	0.000239	0.000537

STAR	0.086474	0.248772	1.52449	2.82E-05	7.73E-05
PATJ-DT	0.074099	0.159196	1.103286	1.95E-08	1.07E-07
G2E3-AS1	0.028298	0.257983	3.188488	6.44E-17	2.40E-15
BNIP3P11	0.278845	0.602378	1.111209	2.64E-15	6.97E-14
FSIP2	0.014547	0.046021	1.661606	1.86E-08	1.03E-07
GAPDHP14	0.187109	0.591504	1.660511	2.43E-05	6.80E-05
DPY19L2	0.036481	0.084189	1.206495	0.012401	0.019024
MZB1	1.339737	4.152278	1.631953	0.008389	0.013405
KY	0.00403	0.017576	2.124662	2.91E-05	7.96E-05
PDZD7	0.046241	0.10806	1.224581	4.25E-11	4.26E-10
IL2RA	0.295579	0.611296	1.04833	1.33E-06	4.92E-06
FAM181B	0.008651	0.023486	1.440839	0.021799	0.031653
FBLL1	0.731912	1.634282	1.158915	9.61E-07	3.67E-06
MSC	3.474064	14.81655	2.092514	9.25E-06	2.85E-05
LINC01952	0.101997	0.266601	1.386162	2.66E-10	2.19E-09
ATAD5	0.294512	0.680456	1.208177	7.67E-22	8.95E-20
SCIRT	0.445639	0.927686	1.05776	7.06E-14	1.34E-12
ASNS	1.438234	3.742638	1.379757	3.73E-11	3.79E-10
PDGFRL	0.487615	1.167561	1.259685	1.99E-09	1.37E-08
SRSF12	0.140414	0.391948	1.480972	6.16E-16	1.86E-14
SLC7A10	0.142942	1.890493	3.725264	1.93E-11	2.09E-10
POU6F2	0.028429	0.08348	1.554083	2.15E-05	6.08E-05
MIR4292	1.2764	2.786965	1.126615	2.77E-14	5.80E-13
LINC00923	0.033756	0.087402	1.372536	0.000245	0.000548
CYP3A4	514.8884	175.8981	-1.54952	1.47E-09	1.04E-08
ZNF426-DT	0.251707	0.526183	1.063821	3.75E-08	1.95E-07
IMPDH1	2.508486	6.026219	1.264436	1.10E-12	1.59E-11
EML5	0.011496	0.023094	1.006447	0.002855	0.005092
SLC10A4	0.020638	0.110223	2.417039	0.000838	0.001672
ANK2	0.104605	0.286613	1.454153	0.010366	0.016214
PIANP	0.024969	0.114046	2.191429	0.023968	0.03446
BNIP3P30	0.041328	0.164485	1.99277	1.05E-08	6.10E-08
CCT5P1	0.007929	0.01684	1.086729	4.46E-07	1.83E-06
TREM1	0.211882	0.459807	1.117768	6.12E-05	0.000157
CSF3R	0.415504	1.234008	1.570416	5.46E-05	0.000141
ZPLD1	0.041172	0.945536	4.521405	1.78E-11	1.95E-10
KCTD16	0.003218	0.015231	2.242925	0.017579	0.0261
CFAP45	0.101518	0.288052	1.504597	5.29E-15	1.30E-13
FABP3	3.082825	13.09909	2.087141	5.72E-07	2.29E-06
CCNE1	1.364075	5.617806	2.042084	6.22E-18	3.03E-16
CRTAM	0.16314	0.340657	1.062214	0.019855	0.029088
SLC10A1	89.36316	38.68544	-1.20789	7.52E-15	1.80E-13
BANK1	0.077785	0.251529	1.69316	0.002598	0.004673
TMSB15A	0.148329	0.641702	2.113104	7.14E-05	0.00018

PEG10	15.60344	48.49998	1.63612	1.53E-11	1.69E-10
PLK1	1.300353	4.227273	1.700823	3.35E-25	1.11E-22
CBY2	0.028726	0.112883	1.974393	2.49E-13	4.13E-12
LINC01954	0.105583	0.212345	1.00804	7.90E-05	0.000197
RAB3IL1	2.100785	4.591209	1.127946	4.19E-08	2.15E-07
GAPDHP59	0.044419	0.105019	1.241421	1.60E-07	7.22E-07
MUC1	1.087563	3.094648	1.508677	0.003659	0.006359
ATP5MC1P4	0.214722	0.709739	1.72482	6.10E-21	5.76E-19
CCDC177	0.4484	0.155194	-1.53072	0.000303	0.000665
BACE2	2.878109	6.505761	1.176597	0.003786	0.006554
HMGA2-AS1	0.002664	0.010606	1.992902	3.81E-05	0.000102
ZNF730	0.017145	0.084311	2.297951	1.13E-15	3.28E-14
TICRR	0.171093	0.541324	1.661715	1.45E-26	1.13E-23
LINC02802	0.152876	0.459779	1.588581	1.10E-07	5.14E-07
SP3P	0.031008	0.160714	2.373803	3.43E-08	1.79E-07
FBXO5	0.663135	1.537142	1.212875	5.82E-17	2.20E-15
SNORD3B-2	0.024231	0.071523	1.561546	0.000155	0.000363
HPD	764.226	297.7992	-1.35966	2.86E-17	1.18E-15
HIF1A-AS1	0.025762	0.075356	1.548467	0.000242	0.000543
CPHL1P	0.091465	0.465963	2.348915	4.32E-07	1.78E-06
RARRES2P8	0.562675	1.906865	1.760827	1.80E-08	9.99E-08
PRODH	2.371424	1.153042	-1.04031	0.000812	0.001625
SNORD3A	0.027135	0.056187	1.050077	0.000101	0.000246
TRPC7-AS1	0.098557	0.215381	1.127857	0.000204	0.000464
PRKAR2B	0.284666	0.577208	1.01982	0.004618	0.007826
CARD14	0.065537	0.166351	1.343842	1.12E-06	4.20E-06
C8orf34	0.002303	0.013714	2.57397	0.021264	0.030944
FOXJ1	0.698916	2.142257	1.61594	4.01E-10	3.18E-09
CLDN6	0.072598	0.402137	2.469694	0.000566	0.001173
MROH3P	0.046357	0.141856	1.613575	0.005953	0.009828
HK2-DT	0.149012	0.456237	1.614356	5.89E-07	2.35E-06
COL4A6	0.048019	0.196609	2.03366	0.000172	0.000398
C1GALT1C1L	0.093081	0.319403	1.778821	1.18E-05	3.55E-05
CYP26B1	0.233083	1.175759	2.334676	4.40E-07	1.81E-06
STXBP5L	0.002146	0.008406	1.969537	2.78E-09	1.84E-08
HOXC-AS2	0.038124	0.080753	1.082805	0.009803	0.015414
SNORD99	1.366801	2.988584	1.128659	9.37E-10	6.91E-09
MAGEA6	1.568133	8.180492	2.38314	5.64E-12	6.89E-11
LRRC7	0.034728	0.081864	1.237126	0.001522	0.002885
LINC02610	0.055581	0.132653	1.254999	0.012841	0.019641
KRT20	1.523117	7.085507	2.217844	0.001261	0.00243
FDCSP	6.14085	16.89063	1.459713	0.005545	0.009221
SLIT1	0.029553	0.072455	1.293791	1.66E-07	7.47E-07
ASB9P1	0.275563	0.63345	1.200848	0.008738	0.013914

ERFL	0.192223	0.60294	1.649236	0.000154	0.00036
TRMT112P4	1.401946	0.696144	-1.00997	1.05E-09	7.66E-09
GAU1	0.017882	0.070506	1.979276	0.00023	0.000518
CSRNP3	0.041611	0.105224	1.338443	7.87E-09	4.73E-08
KLK14	0.056194	0.117499	1.064157	0.001788	0.003338
FAM30A	0.032642	0.119919	1.877272	0.011981	0.018441
NUF2	1.050025	3.410263	1.699459	1.40E-22	1.94E-20
SHOC1	0.013859	0.04323	1.641178	0.000755	0.001523
KRT6B	0.130773	0.285943	1.128664	6.46E-07	2.56E-06
DCAF4L2	1.400565	5.267457	1.911098	3.19E-06	1.08E-05
CLRN2	0.04567	0.021435	-1.09126	3.64E-05	9.74E-05
DDX4	0.009394	0.026621	1.502812	0.000444	0.000941
ORC6	0.434413	1.273042	1.55114	3.15E-23	5.25E-21
LINC00334	0.020763	0.046372	1.159224	8.60E-06	2.67E-05
CCDC163	0.55554	1.21871	1.133392	1.78E-22	2.35E-20
BCAT1	0.358737	1.087062	1.599437	1.32E-08	7.53E-08
TENM1	0.300984	0.620714	1.044242	2.78E-05	7.66E-05
UPK1A-AS1	0.194626	0.497849	1.355001	4.42E-06	1.46E-05
GINS2	1.728678	3.735365	1.11158	6.21E-20	4.69E-18
FOXP4-AS1	0.245248	0.622547	1.343943	0.001649	0.003101
PIK3CD-AS1	0.013739	0.028951	1.075335	0.000207	0.000471
SAPCD2	0.538731	1.453592	1.431986	1.84E-16	6.26E-15
EGFR-AS1	0.433511	1.332264	1.61974	0.005922	0.009785
BCL2L12P1	0.012495	0.041471	1.730704	1.05E-07	4.95E-07
EID2B	0.381883	0.79148	1.051422	1.70E-22	2.27E-20
SPAG17	0.010018	0.03245	1.695634	3.56E-06	1.20E-05
SBK3	0.120983	0.281378	1.217711	0.000596	0.001229
LINC02872	0.004568	0.018157	1.990893	0.00294	0.005226
MIAT	0.093641	0.259503	1.470542	0.002224	0.004059
PDCD6IPP2	0.028441	0.124253	2.127253	1.22E-05	3.65E-05
MKRN4P	0.042065	0.256567	2.608636	3.05E-07	1.30E-06
NDUFA4L2	6.066549	15.49845	1.353176	2.73E-08	1.46E-07
MYEF2	0.160822	0.374638	1.220036	3.90E-08	2.02E-07
ZNF682	0.384929	0.867382	1.172074	3.62E-13	5.81E-12
GNGT1	0.023825	0.209846	3.13877	5.41E-19	3.46E-17
OTX1	0.203121	0.43419	1.095988	1.93E-08	1.06E-07
TMEM255A	0.208439	0.54289	1.381034	0.005431	0.009052
CUZD1	0.063104	1.009551	3.999829	2.08E-13	3.55E-12
RSPO4	0.040055	0.201545	2.331055	0.007222	0.011708
HOXB13	0.144776	0.469671	1.697829	2.06E-08	1.13E-07
NR0B1	0.06003	0.822884	3.776946	1.22E-08	7.01E-08
LINC00668	0.035232	0.204774	2.539055	5.94E-11	5.73E-10
ROR1-AS1	0.007322	0.029168	1.994015	1.87E-06	6.69E-06
ECT2	1.574149	3.913601	1.313924	4.37E-21	4.23E-19

LINC01901	0.024452	0.135801	2.473492	3.42E-08	1.79E-07
CTHRC1	3.335022	6.795548	1.026894	5.17E-07	2.09E-06
SSTR3	0.033459	0.136206	2.02534	6.92E-09	4.20E-08
CYP4F26P	0.02962	0.074184	1.324525	0.002547	0.00459
ENTHD1	0.009648	0.056405	2.547498	0.000722	0.001462
IBSP	0.144046	1.263911	3.133295	8.81E-14	1.64E-12
OLFML3	2.697932	6.326943	1.229655	8.23E-07	3.19E-06
CYP1A2	42.8636	6.980169	-2.61842	4.43E-07	1.82E-06
CACNG4	1.034877	2.643314	1.352889	0.004233	0.007243
COL6A4P1	0.008441	0.028259	1.743176	1.01E-14	2.35E-13
PYY	0.079837	0.212162	1.410033	0.006957	0.011308
POU3F2	0.025089	0.090825	1.856047	5.22E-09	3.25E-08
C12orf56	0.022943	0.220272	3.26318	4.06E-14	8.12E-13
MMD	3.657123	8.364898	1.193639	1.78E-18	9.77E-17
EMID1	1.748428	3.970852	1.18339	0.00012	0.000287
ZNF45-AS1	0.209168	0.569503	1.445038	1.46E-10	1.28E-09
LINC01882	0.077761	0.207367	1.415064	2.19E-06	7.73E-06
HOXD4	0.075894	0.177966	1.229555	0.020798	0.030345
LRP8	0.109808	0.299329	1.446742	2.84E-08	1.51E-07
C8orf88	0.227036	0.684956	1.593093	1.22E-05	3.65E-05
MELK	1.351298	3.766203	1.478765	1.07E-22	1.54E-20
TEX15	0.01692	0.063186	1.900895	7.54E-07	2.94E-06
DUXAP10	0.035643	0.080852	1.181686	8.72E-12	1.02E-10
C1orf194	0.022665	0.047603	1.070605	1.72E-05	4.99E-05
TMSB10P1	0.329607	0.717597	1.122427	1.10E-05	3.32E-05
DIRAS1	0.320916	0.684381	1.092602	0.000665	0.001358
MAGEC2	2.410759	7.792109	1.692527	5.93E-06	1.91E-05
MAGEC1	0.538401	1.840615	1.773434	3.43E-05	9.23E-05
CSPG4BP	0.007356	0.023816	1.694874	3.44E-05	9.26E-05
HTR2A-AS1	0.058788	0.022388	-1.3928	1.94E-09	1.34E-08
SLC25A24	0.677907	1.462704	1.109479	4.21E-08	2.16E-07
NEFM	0.007476	0.027758	1.892591	1.83E-05	5.27E-05
UBA52P6	0.203012	0.417442	1.040007	6.12E-11	5.88E-10
ARHGEF2	2.159862	4.759004	1.13972	1.25E-17	5.75E-16
SKP2	3.688396	7.6772	1.057587	2.97E-18	1.56E-16
RUNDC3A	0.061929	0.173757	1.488396	1.49E-10	1.31E-09
GRIA4	0.002221	0.013328	2.585366	2.54E-08	1.36E-07
CCNA2	2.780772	7.577609	1.446257	2.81E-21	2.79E-19
SPAG6	0.010173	0.064615	2.667148	5.74E-11	5.56E-10
MAFA	0.556283	1.773283	1.672531	0.000517	0.00108
GALNT15	0.815087	0.406734	-1.00287	5.89E-10	4.53E-09
GSTM1	29.98425	9.45905	-1.66444	0.003428	0.005991
CAPN6	0.861878	2.256231	1.38836	1.45E-05	4.27E-05
SCN1A	0.010853	0.08163	2.911035	1.25E-05	3.74E-05

ELFN1-AS1	0.412715	1.425695	1.788447	0.009801	0.015413
LRRC74B	0.001497	0.004922	1.716793	3.16E-07	1.34E-06
FRMD1	0.043079	0.169533	1.976521	1.70E-06	6.15E-06
TNFRSF11B	2.076469	4.417651	1.089147	0.000498	0.001044
NEFH	0.21116	0.437484	1.050892	2.69E-05	7.43E-05
MMS22L	0.135339	0.31547	1.220927	4.30E-20	3.35E-18
BCYRN1	0.027534	0.081568	1.566807	1.53E-06	5.57E-06
RPL29P14	0.084644	0.170967	1.01424	2.59E-10	2.14E-09
SH2D6	0.096827	0.207097	1.096819	7.02E-09	4.25E-08
C10orf88B	0.064966	0.225622	1.796161	2.12E-17	9.23E-16
LINC02641	0.013285	0.047051	1.824394	2.02E-07	8.96E-07
LINC02438	0.101584	0.283642	1.481398	1.44E-06	5.29E-06
LINC01993	0.031624	0.083405	1.399108	0.003119	0.005507
CORO6	0.109498	0.253825	1.212933	1.08E-05	3.28E-05
FANCG	1.986879	4.076711	1.036902	2.68E-25	1.01E-22
SLCO6A1	0.038841	0.180837	2.219023	3.45E-11	3.54E-10
MCCD1	0.949687	3.963778	2.061353	0.000237	0.000533
PLD5	0.00979	0.264832	4.757646	0.000906	0.001795
SEPTIN14P12	0.045936	0.133099	1.534818	3.25E-09	2.12E-08
GPR161	0.172805	0.381702	1.143296	2.53E-05	7.04E-05
GPC2	0.064912	0.167027	1.363524	9.97E-14	1.83E-12
MALRD1	0.008384	0.039649	2.241571	6.61E-10	5.03E-09
USH1G	0.02474	0.059643	1.269522	0.001207	0.002334
MAEL	0.215799	2.055068	3.251424	4.38E-05	0.000115
NCLP1	0.016008	0.033258	1.054941	2.97E-08	1.58E-07
STEAP1B	0.042121	0.265135	2.654103	1.78E-15	4.87E-14
CERS1	0.13719	0.363281	1.404913	4.41E-06	1.46E-05
RPS7P3	0.098116	0.196806	1.00421	9.22E-10	6.81E-09
ASB11	0.022488	0.104633	2.218126	0.000427	0.000907
PPP2R2B	0.050386	0.175665	1.801736	9.04E-09	5.34E-08
PLAAT5	0.037291	0.128951	1.789912	0.00186	0.003455
TRHDE	0.116717	0.3141	1.428213	3.55E-06	1.19E-05
BPIFB4	0.069008	0.14633	1.084398	0.005809	0.009617
P3H1	3.8838	8.651326	1.155452	2.65E-62	5.39E-58
CRYBB2	0.099525	0.209811	1.075955	0.00012	0.000287
CCDC74B	0.049693	0.103697	1.061268	1.44E-12	2.02E-11
LINC01121	0.028687	0.080435	1.487436	1.35E-07	6.20E-07
TOMM40P4	0.185216	0.506448	1.451206	3.73E-14	7.49E-13
NKAIN2	0.136387	0.402994	1.56305	2.83E-07	1.21E-06
SNHG4	0.293852	0.62056	1.078481	4.73E-06	1.55E-05
BARX1	0.264097	0.709783	1.426309	4.00E-05	0.000106
CNTN1	0.212405	1.019458	2.262912	0.004629	0.007842
NXPH4	1.919791	4.62451	1.268351	1.04E-10	9.52E-10
DNMT3B	0.454375	0.981809	1.111559	4.02E-14	8.04E-13

PPIAP74	0.043576	0.096647	1.149179	0.027738	0.039309
IGHV3-43	1.282413	3.834727	1.580263	0.004956	0.008333
TMC5	1.162971	2.574191	1.146303	0.002104	0.003859
LY6H	0.238909	0.484119	1.018903	2.49E-07	1.08E-06
KLHL6-AS1	0.64642	1.466945	1.182271	0.007748	0.012473
NTRK1	0.023437	0.086684	1.886988	8.18E-05	0.000203
RNASEH2A	6.849911	14.14486	1.04612	2.64E-22	3.37E-20
ZNF90	0.044819	0.280595	2.646325	4.67E-18	2.36E-16
ADH4	307.5245	148.9895	-1.04549	8.34E-09	4.98E-08
NEBL	0.377205	0.798719	1.08234	0.015767	0.023642
EPO	1.016711	4.79289	2.236986	1.90E-09	1.31E-08
DSCR8	0.317895	2.081566	2.711045	0.000237	0.000533
CFAP300	0.027943	0.072042	1.366335	4.44E-06	1.46E-05
SNORD46	0.327315	0.673535	1.041072	1.37E-09	9.74E-09
TK1	12.90574	27.57106	1.095142	5.67E-18	2.80E-16
CELF4	0.026462	0.101556	1.940257	2.94E-10	2.41E-09
FIRRE	0.062752	0.147053	1.228602	2.21E-10	1.85E-09
BNIP3P27	0.033518	0.092304	1.461468	1.90E-10	1.63E-09
PPM1E	0.243659	0.589364	1.274298	0.00041	0.000875
RAB38	0.332674	0.854445	1.36088	9.09E-09	5.37E-08
SLC25A5P5	0.055168	0.161033	1.545464	5.54E-09	3.44E-08
SIGLEC10-AS1	0.011165	0.027225	1.285931	0.000245	0.000547
ZNF204P	0.473362	1.158841	1.291667	1.30E-07	5.98E-07
RPH3A	0.004888	0.014187	1.537212	0.011474	0.017744
NCAPH	1.144062	2.946712	1.364941	6.00E-20	4.56E-18
MPP4	0.012198	0.025748	1.077834	2.62E-07	1.13E-06
FABP6	0.083352	0.811801	3.283835	1.52E-10	1.33E-09
LINC00941	0.101735	0.308313	1.599577	4.48E-06	1.47E-05
HERC2P3	0.132132	0.342596	1.374534	6.95E-07	2.73E-06
KLRC2	0.020792	0.055963	1.428475	0.001774	0.003316
SORCS1	0.015124	0.097728	2.691904	1.09E-08	6.33E-08
MND1	1.189397	2.450182	1.042659	4.41E-16	1.37E-14
ALOX15B	0.876882	2.791482	1.670577	0.000798	0.001599
IMPDH1P8	0.025198	0.067238	1.41597	1.06E-10	9.68E-10
KCP	0.133124	0.269274	1.016303	5.57E-05	0.000144
TRHDE-AS1	0.077886	0.285146	1.872271	7.83E-05	0.000195
ZNF439	0.128347	0.340039	1.405646	2.85E-10	2.34E-09
AFP	41.88294	374.1831	3.15931	3.05E-09	2.00E-08
FAXC	0.023528	0.051997	1.144064	9.39E-06	2.89E-05
OPRD1	0.016471	0.062217	1.917349	6.72E-10	5.10E-09
KCNK3	0.06606	0.166727	1.335639	0.022756	0.03289
LINC02518	0.02744	0.078544	1.5172	0.000356	0.000771
SERPINH1	14.30574	30.67424	1.100433	2.51E-16	8.24E-15
LINC02829	0.02677	0.057427	1.101079	0.000349	0.000757

LINC00158	0.010934	0.029916	1.452043	3.18E-05	8.62E-05
INSL6	0.043504	0.349968	3.008006	2.33E-05	6.53E-05
LINC00689	0.027053	0.097922	1.85584	1.46E-10	1.28E-09
ZNF648	0.535588	0.265522	-1.01229	7.55E-05	0.000189
DTX3	1.211444	2.506759	1.049096	6.55E-08	3.22E-07
ERVMER34-1	0.153676	0.58346	1.924742	2.92E-07	1.25E-06
PCYT1B	0.022291	0.140769	2.658777	2.20E-05	6.20E-05
SSX1	2.931398	13.07839	2.157524	8.57E-09	5.10E-08
EXD1	0.005487	0.01454	1.405916	5.86E-06	1.89E-05
FCHO1	0.360282	0.835479	1.213476	4.65E-08	2.37E-07
NKAIN1	0.027465	0.074527	1.440195	8.29E-09	4.96E-08
FER1L4	0.248908	0.540177	1.117818	5.10E-14	9.97E-13
BNIP3P9	0.060386	0.193277	1.678373	5.16E-06	1.68E-05
RTL8B	1.007254	2.389936	1.246545	0.000194	0.000445
HOXB9	0.090171	0.471324	2.385988	0.005269	0.008808
ZNF385C	0.199813	0.411762	1.043159	0.00294	0.005226
IQCA1	0.063961	0.325994	2.349589	9.53E-08	4.52E-07
FBLN1	3.638664	11.8009	1.697417	1.68E-06	6.08E-06
SHOX2	0.043073	0.174868	2.021429	7.71E-12	9.15E-11
CEP295NL	0.007413	0.020077	1.437428	6.56E-07	2.59E-06
S100B	0.386963	0.82472	1.091709	9.04E-05	0.000222
CFAP47	0.012609	0.032975	1.386966	0.000404	0.000864
KRT8P14	0.017757	0.038694	1.123703	1.34E-07	6.15E-07
SLC31A1P1	0.038426	0.149931	1.96413	6.97E-06	2.21E-05
ERBB4	0.004997	0.0276	2.465605	0.008391	0.013407
AURKB	2.0836	6.34321	1.606135	2.16E-21	2.22E-19
C21orf58	0.540285	1.085527	1.006602	3.98E-17	1.60E-15
FGF11	0.009223	0.019325	1.067131	0.000172	0.000397
SLC17A8	0.017714	0.049144	1.4721	0.000426	0.000906
SERPINI1	1.606487	4.966053	1.62819	3.17E-14	6.51E-13
MIR155HG	0.239908	0.493562	1.040748	1.07E-06	4.05E-06
GPX8	0.881345	1.862844	1.079729	0.000456	0.000963
ARHGAP11A	0.807952	2.074629	1.360512	1.90E-21	1.98E-19
TUBA4B	0.267281	0.62128	1.216885	0.014222	0.02154
LINC01208	0.035604	0.098219	1.463967	0.003219	0.005662
GIHCG	1.282862	2.585641	1.011155	1.41E-19	1.03E-17
LEP	0.011197	0.181876	4.021751	0.001626	0.003061
UHRF1	0.739271	2.063354	1.480816	1.16E-18	6.79E-17
SYT1	0.355148	1.088637	1.61603	2.17E-13	3.70E-12
FBXL7	0.877106	1.760707	1.005331	1.55E-07	7.04E-07
SNORD88A	0.108826	0.248183	1.189384	2.03E-06	7.22E-06
LGALS12	0.026094	0.429004	4.039202	7.59E-06	2.38E-05
SLC2A5	0.961048	2.118224	1.140175	5.04E-07	2.05E-06
ELAVL4	0.009353	0.027325	1.546643	6.28E-08	3.11E-07

SMARCE1P6	0.029117	0.073317	1.332289	4.54E-06	1.49E-05
MCM7	12.87483	28.63966	1.153461	2.81E-26	1.68E-23
CENPO	0.798655	1.776766	1.15361	6.53E-24	1.37E-21
ZFP57	0.047218	0.226489	2.262028	0.003097	0.005472
CD300LG	0.367881	0.13593	-1.43638	1.40E-14	3.17E-13
DDX11-AS1	0.118447	0.271937	1.199034	1.51E-18	8.58E-17
MEG8	0.020599	0.10273	2.318189	0.018417	0.027197
CD19	0.188402	0.513047	1.445279	7.20E-05	0.000181
H2AC12	0.044742	0.101253	1.178271	1.80E-06	6.47E-06
LINC01139	0.231279	1.540633	2.735816	5.80E-13	8.96E-12
IGF2BP2	2.360398	5.678172	1.266396	7.33E-13	1.10E-11
HECW1	0.028538	0.073008	1.355169	0.00456	0.007742
MEX3A	0.676218	1.7851	1.400444	1.99E-16	6.73E-15
ITLN1	0.199413	0.671648	1.751946	0.000471	0.000992
PIP5KL1	0.074544	0.152252	1.030293	1.59E-09	1.12E-08
PLA2G4D	0.005668	0.012875	1.183727	5.18E-07	2.10E-06
TRAV39	0.06633	0.150761	1.184538	0.026093	0.037233
PLEKHB1	0.489806	2.889082	2.560329	9.26E-10	6.84E-09
NPSR1-AS1	0.101726	0.256136	1.332219	6.13E-07	2.44E-06
FCGR1B	0.065534	0.157161	1.261938	2.94E-07	1.26E-06
DUXAP9	0.050426	0.112032	1.151677	2.16E-12	2.88E-11
PAGE2B	2.312392	9.943939	2.104431	3.94E-09	2.52E-08
CLMP	0.14866	0.413019	1.474194	0.01432	0.021669
GLB1L3	0.013022	0.093449	2.84328	8.77E-09	5.21E-08
SDS	239.1467	86.74463	-1.46305	4.36E-07	1.79E-06
C5orf58	0.156381	0.548585	1.810648	4.02E-10	3.18E-09
CYCSP6	0.222215	0.535408	1.268685	3.67E-05	9.81E-05
PRDM9	0.01632	0.060411	1.888137	3.76E-06	1.26E-05
LINC00632	0.006452	0.136524	4.403229	1.82E-11	1.99E-10
TEDC2	1.044115	2.715176	1.378765	3.35E-24	7.56E-22
MARCKS	14.11669	29.86542	1.081074	1.35E-15	3.81E-14
DNAH8	0.010137	0.030621	1.594875	4.97E-07	2.02E-06
HBE1	0.105866	0.644908	2.606859	5.38E-05	0.000139
CALB2	0.065903	0.29822	2.177967	3.31E-05	8.95E-05
G3BP1P1	0.009509	0.034755	1.869888	2.31E-12	3.06E-11
KRT18P5	0.049732	0.115416	1.214598	1.10E-13	2.00E-12
LINC00491	0.046946	0.214673	2.193078	6.70E-12	8.02E-11
LINC01287	0.854171	5.032252	2.558607	7.12E-07	2.79E-06
OLFML2B	2.076978	5.020101	1.27323	1.81E-07	8.07E-07
UCHL1	0.608582	14.36752	4.561216	2.04E-06	7.24E-06
KIF4B	0.002332	0.006489	1.476391	5.23E-07	2.11E-06
LINC01684	0.011167	0.025929	1.215353	0.002284	0.00416
B3GNT7	0.783994	1.73723	1.147874	0.000973	0.001914
SULT1B1	3.684179	1.534801	-1.26329	7.33E-08	3.57E-07

SLCO5A1-AS1	0.021425	0.043932	1.035989	0.001263	0.002433
LINC00501	0.016394	0.092425	2.495147	5.92E-09	3.65E-08
TUBA3C	0.230702	5.229442	4.502553	1.69E-10	1.46E-09
CRYBA4	0.028569	1.310931	5.519973	0.008934	0.014193
LINC01694	0.031978	0.178329	2.479387	6.45E-10	4.91E-09
C2CD6	0.021073	0.046939	1.15536	0.012115	0.018634
GIPR	0.187342	0.487308	1.379163	1.22E-08	6.99E-08
LINC01730	0.103669	0.278527	1.425838	2.70E-08	1.45E-07
SLC8A2	0.014415	0.052088	1.8534	0.00029	0.000639
C8orf48	0.102941	0.210562	1.032427	8.27E-05	0.000205
BMF	2.179072	4.434975	1.025213	2.28E-12	3.02E-11
TTC36	17.0493	7.543827	-1.17634	1.73E-09	1.20E-08
MSX1	0.79057	1.659741	1.069993	0.017848	0.026454
RPL39P18	0.071205	0.165977	1.220928	5.03E-06	1.64E-05
TEX11	0.25574	0.626367	1.292328	0.001853	0.003443
RPS8P4	0.028487	0.088885	1.641653	0.000125	0.000299
TRIP13	0.791887	2.842151	1.843616	1.16E-25	5.03E-23
MIR1295A	2.528746	1.16909	-1.11304	1.41E-06	5.19E-06
CLSTN1	7.011635	15.32108	1.127696	7.35E-15	1.76E-13
LINC00173	0.051645	0.137993	1.41789	8.59E-06	2.66E-05
ITPKA	2.677901	6.08106	1.18322	1.61E-11	1.77E-10
TREH	1.741756	0.835568	-1.05971	8.08E-08	3.89E-07
PABPN1P2	0.010638	0.023897	1.167634	1.57E-05	4.59E-05
ANKLE1	0.065874	0.233436	1.825234	8.27E-13	1.23E-11
GAPDHP32	0.030463	0.062214	1.030154	0.000693	0.00141
ZNF556	0.017655	0.074134	2.07007	4.14E-11	4.16E-10
ZNF99	0.031855	0.151016	2.245109	4.17E-08	2.14E-07
STRA6	0.090123	0.203162	1.172671	1.18E-05	3.55E-05
CREG2	0.024009	0.060516	1.333721	6.44E-11	6.15E-10
DMC1	0.077989	0.211373	1.43845	9.80E-11	9.03E-10
CTH	40.24681	19.09093	-1.07599	1.60E-07	7.22E-07
LINC01191	0.055289	0.133861	1.275683	6.60E-06	2.10E-05
ACSM4	0.021528	0.043213	1.005264	0.002209	0.004036
VAT1L	0.626837	1.296107	1.048022	0.002212	0.004041
HEPACAM2	0.059316	0.121242	1.031412	0.002796	0.004999
GTSF1	0.805983	4.577797	2.505832	1.70E-10	1.47E-09
LHFPL3-AS2	0.202674	0.900101	2.150924	5.18E-12	6.38E-11
FABP5	1.976012	5.121372	1.373939	1.20E-10	1.08E-09
CDCP2	0.004514	0.010716	1.247356	3.67E-07	1.53E-06
NXPE3	0.254745	0.605006	1.247893	4.33E-06	1.43E-05
DYNLL1P4	0.045495	0.106583	1.22821	3.44E-06	1.16E-05
RNFT2	0.17398	0.496221	1.512062	4.58E-21	4.39E-19
GJB7	0.00906	0.042201	2.219777	0.000801	0.001604
ARHGEF39	0.527819	1.217648	1.205981	2.27E-23	3.97E-21

FAM27E3	0.032751	0.082866	1.339235	6.79E-06	2.16E-05
ZIM2	0.01464	0.046127	1.655745	8.87E-07	3.41E-06
LINC02607	0.012144	0.067637	2.477557	1.07E-05	3.25E-05
PIGAP1	0.00414	0.014335	1.791931	4.47E-10	3.51E-09
SATB1-AS1	0.010674	0.048164	2.173866	7.12E-07	2.79E-06
TROAP-AS1	0.003646	0.008174	1.164607	6.62E-07	2.61E-06
SIX1	0.159061	0.320638	1.011367	0.000294	0.000645
ZNF714	0.113476	0.311527	1.456974	3.59E-14	7.27E-13
PKM	15.10973	35.73843	1.241998	2.99E-19	2.05E-17
LINC01960	0.010067	0.03456	1.779475	1.75E-05	5.05E-05
HNRNPA1P54	0.003101	0.006949	1.164334	8.60E-07	3.32E-06
ZBTB8B	0.002039	0.004569	1.164052	0.000414	0.000882
NOVA1-DT	0.091237	0.311579	1.771906	3.01E-10	2.46E-09
PODXL2	3.230358	6.860735	1.086669	1.07E-06	4.06E-06
CDC37P1	0.03276	0.065699	1.003917	0.006601	0.010781
SYN3	0.085201	0.204713	1.264662	0.000213	0.000483
PSMA8	0.004747	0.1403	4.885301	1.20E-09	8.63E-09
TM4SF19	0.111696	0.340478	1.607981	0.012509	0.019175
CADPS	0.035334	0.200417	2.503873	0.009749	0.015343
DGKI	0.024166	0.052492	1.119118	2.36E-05	6.61E-05
PAX9	0.012448	0.046149	1.890424	0.000831	0.00166
KCTD19	0.003838	0.009273	1.272875	0.000129	0.000307
LTO1	1.384979	4.330513	1.644673	4.17E-10	3.29E-09
RNA5SP283	0.170074	0.385922	1.18215	0.023266	0.033534
CRYGS	0.676779	2.494218	1.881831	3.62E-09	2.33E-08
RPS2P32	0.264061	0.642492	1.282808	8.23E-11	7.71E-10
OMG	0.080148	0.422802	2.399239	0.012949	0.019791
LINC02043	0.021056	0.070197	1.737206	4.54E-12	5.64E-11
GPNMB	7.425539	15.45078	1.057112	0.000272	0.000601
LINC00470	0.166874	0.448115	1.425115	2.98E-07	1.27E-06
SLC22A1	111.0856	52.1254	-1.09161	5.88E-08	2.92E-07
CABP7	0.074435	0.170867	1.198814	3.11E-08	1.64E-07
TET1	0.09516	0.279669	1.555302	1.55E-18	8.75E-17
LSP1P4	0.238858	0.508868	1.091134	3.68E-09	2.37E-08
KCNH8	0.040572	0.116102	1.516823	3.81E-08	1.98E-07
BRDT	0.002035	0.264575	7.02233	5.46E-10	4.22E-09
GPR84	0.09905	0.305312	1.624047	1.23E-08	7.09E-08
TMEM156	1.464191	2.970714	1.020706	0.000619	0.001272
GRM3	0.011123	0.097769	3.135819	1.64E-06	5.92E-06
LINC00652	0.013835	0.034067	1.300019	3.31E-05	8.95E-05
S100A3	0.349573	0.964718	1.464511	2.79E-05	7.67E-05
SLC1A5	4.021724	13.72177	1.770581	1.08E-14	2.48E-13
HCAR2	0.139893	0.433934	1.633149	4.48E-07	1.84E-06
CDKN2C	3.636094	7.997918	1.137235	3.90E-18	2.00E-16

HELLS	0.49033	1.195473	1.285756	3.69E-20	2.89E-18
DUSP5P1	0.07376	0.185448	1.330105	0.000117	0.000281
CTRC	0.011808	0.033252	1.493648	0.000924	0.001827
CDKN3	3.0768	7.439114	1.2737	3.56E-19	2.40E-17
TIGIT	0.308846	0.674763	1.127492	3.14E-05	8.53E-05
TACC3	2.573592	6.967597	1.436878	1.38E-27	2.16E-24
FCRL5	0.043646	0.115969	1.409809	0.01613	0.024146
DDX53	0.07548	0.277115	1.876312	4.84E-07	1.97E-06
LINC00494	0.157782	0.37339	1.242751	1.53E-06	5.57E-06
CDKN2A-DT	0.04831	0.111041	1.200689	1.73E-08	9.64E-08
HMGA2	0.039726	0.478	3.588858	3.30E-12	4.20E-11
WDR62	0.414263	1.18115	1.511573	3.39E-25	1.11E-22
CHRNA2	0.009397	0.03183	1.76009	0.005426	0.009046
LINC01572	0.024048	0.064592	1.425427	1.77E-13	3.09E-12
B3GNT5	0.850536	2.045279	1.265853	1.14E-06	4.27E-06
SBK1	0.244397	0.514018	1.07259	6.21E-06	1.99E-05
CHML	1.386859	2.940532	1.084256	8.30E-14	1.56E-12
TSPAN10	0.483984	1.432718	1.565723	0.000734	0.001483
IGHV7-81	0.110503	0.33382	1.59499	0.000128	0.000305
ETV5	1.788685	3.85882	1.109261	4.86E-15	1.21E-13
MYPN	0.004376	0.010782	1.300979	0.000598	0.001233
WTAPP1	0.024255	0.076863	1.664006	5.82E-08	2.90E-07
SMKR1	0.243757	0.525376	1.107909	0.001009	0.001979
ZNF229	0.243175	0.651117	1.42092	1.19E-09	8.60E-09
CCDC185	0.030649	0.102787	1.745752	2.59E-05	7.18E-05
SPTSSB	0.223101	0.73515	1.720343	5.05E-08	2.55E-07
MATN3	0.435973	2.358143	2.435341	4.72E-06	1.55E-05
TGFB2	0.605281	1.355955	1.163632	0.006298	0.010338
NIBAN1	0.865764	1.785817	1.044539	0.024347	0.034933
BNIP3P17	0.041337	0.3548	3.101498	2.33E-17	9.98E-16
GAPDHP21	0.024047	0.058038	1.271151	1.34E-06	4.96E-06
ZFPM2-AS1	0.810161	2.195496	1.438267	3.68E-06	1.23E-05
ZNF391	0.131172	0.372181	1.504548	8.40E-12	9.88E-11
VCX3A	0.034638	1.018175	4.877498	3.15E-11	3.28E-10
APCDD1L	0.016058	0.148938	3.213346	9.80E-06	3.00E-05
LINC00958	0.033805	0.420056	3.635259	1.81E-08	1.00E-07
STX1A	0.453214	0.937957	1.049331	1.34E-20	1.13E-18
RFC4	3.455119	7.780664	1.171158	2.21E-27	2.80E-24
IGKV2-29	0.640037	3.034899	2.245421	0.006448	0.010559
ABCC8	0.046408	0.161584	1.799834	0.000279	0.000616
ZNF788P	0.144312	0.322767	1.161304	5.62E-11	5.46E-10
RANBP3L	0.609819	0.298945	-1.0285	4.55E-05	0.00012
ZNF812P	0.158408	0.705385	2.154768	0.00029	0.000639
MAGEA8	0.062574	0.97841	3.966802	9.67E-10	7.11E-09

DGKB	0.019524	0.043533	1.156896	0.012876	0.019688
POLR2MP1	0.023096	0.058857	1.349602	0.000124	0.000297
LINC00520	0.009617	0.027006	1.489661	8.45E-05	0.000209
CRB2	0.017121	0.048229	1.49413	0.00037	0.000799
HERC2P8	0.001365	0.005794	2.085833	1.01E-09	7.36E-09
CECR7	0.053056	0.370732	2.8048	8.93E-13	1.32E-11
UTS2R	0.032307	0.083112	1.363217	0.005329	0.008899
RHEX	0.614391	2.031478	1.7253	5.49E-11	5.34E-10
CT75	0.014719	0.080458	2.450587	3.28E-05	8.87E-05
HOXA6	0.041908	0.094836	1.178208	9.31E-07	3.56E-06
RPL7AP28	0.026689	0.225562	3.079201	2.55E-05	7.08E-05
CBX2	0.526868	1.604719	1.606806	6.20E-12	7.49E-11
SLC66A1L	0.025219	0.203768	3.014333	9.80E-09	5.75E-08
PTGDR2	0.578296	1.732813	1.583236	0.002477	0.004477
CNTNAP1	0.437114	1.140398	1.383455	5.76E-12	7.01E-11
IGF2BP1	0.793371	2.952752	1.895992	9.71E-11	8.95E-10
TMEM130	0.074041	0.969817	3.711313	0.000256	0.00057
RNVU1-3	0.21644	0.501075	1.211055	6.85E-08	3.36E-07
RAMP2-AS1	0.166326	0.368807	1.148851	1.66E-05	4.83E-05
PLBD1-AS1	0.213345	0.463969	1.120838	2.20E-06	7.78E-06
LINC00355	0.147325	0.426422	1.533278	2.71E-07	1.17E-06
LINC00664	0.015504	0.071498	2.205275	1.98E-09	1.36E-08
FZD7	0.681315	1.609972	1.240641	1.61E-06	5.84E-06
IGHV1-69	1.07674	6.520772	2.598372	0.012645	0.019367
GABRA2	0.0394	0.31739	3.009986	2.03E-06	7.20E-06
LINC02208	0.004524	0.010823	1.258333	0.002072	0.003805
HERC2P5	0.003153	0.013205	2.066147	4.18E-10	3.30E-09
APCDD1L-DT	0.00404	0.02628	2.701397	5.28E-08	2.66E-07
PRSS45P	0.087062	0.176846	1.02238	1.90E-05	5.45E-05
ADGRD1	0.701296	1.495351	1.09239	0.000786	0.001576
LINC01914	0.128904	0.293963	1.189341	0.001119	0.002176
AFF2	0.032141	0.21022	2.709422	9.17E-07	3.51E-06
KC6	0.031782	0.127187	2.00066	6.64E-06	2.11E-05
LINC02765	0.021804	0.044824	1.039712	7.62E-05	0.00019
SAA2	250.6695	45.20698	-2.47117	1.95E-05	5.57E-05
PNPLA1	0.007426	0.022766	1.616158	1.91E-05	5.47E-05
POU2AF1	0.205733	0.600339	1.545001	5.52E-07	2.22E-06
LINC02280	0.00796	0.016917	1.087714	0.004049	0.006963
LINC01012	0.017381	0.038125	1.133237	4.19E-06	1.39E-05
XIRP1	0.015566	0.048604	1.642703	1.44E-05	4.25E-05
MAP3K4-AS1	0.136091	0.280861	1.045279	8.46E-10	6.31E-09
FAR2P1	0.084644	0.350728	2.050872	1.99E-11	2.15E-10
KNTC1	0.713737	1.782075	1.320094	1.13E-20	9.64E-19
CTAG2	0.992753	11.06046	3.477833	3.08E-12	3.95E-11

SASS6	0.625714	1.317747	1.074498	3.09E-19	2.12E-17
LHFPL4	0.14986	0.333457	1.153887	2.32E-05	6.52E-05
COL8A2	0.416575	0.929148	1.157331	0.000782	0.001569
LINC00189	0.340138	0.899961	1.403742	2.33E-05	6.54E-05
FOXL1	0.148262	0.346797	1.225945	2.73E-06	9.42E-06
NRM	4.537558	10.0112	1.141627	4.00E-20	3.13E-18
CEP55	0.665769	2.296811	1.786539	2.83E-23	4.80E-21
MLXP1	0.028192	0.134398	2.253137	5.32E-05	0.000137
AQP10	0.124864	0.669801	2.42337	7.61E-06	2.39E-05
ATP1A4	0.012204	0.037856	1.633216	0.0023	0.004185
LINC01192	0.007288	0.036358	2.31862	3.80E-10	3.03E-09
GJB6	0.072373	0.686029	3.244738	0.001488	0.002826
GLIS2	1.9581	4.053652	1.049768	3.40E-10	2.74E-09
MYCBPAP	0.023285	0.054594	1.229365	4.47E-08	2.29E-07
PITX2	0.025663	0.215512	3.069987	5.55E-09	3.44E-08
SPIB	0.401751	1.447116	1.848806	5.98E-06	1.92E-05
FOXN4	0.66367	1.605066	1.274095	0.006325	0.010377
ANGPTL1	3.150659	1.355861	-1.21644	2.37E-07	1.03E-06
CDCA5	1.936187	5.133416	1.406701	5.18E-25	1.59E-22
ZNF835	0.136848	0.291473	1.090781	2.04E-06	7.24E-06
SLC2A1	1.298422	3.803465	1.550556	1.11E-08	6.44E-08
CNDP1	1.427215	0.582709	-1.29236	0.003102	0.00548
RPL35P5	1.14405	2.843731	1.313635	3.69E-05	9.86E-05
LINC02228	0.04369	0.545938	3.643357	1.14E-09	8.24E-09
VAX2	0.288827	0.640204	1.148326	0.018986	0.02794
TYRO3	0.557356	1.588369	1.510875	2.28E-14	4.89E-13
MCF2L2	0.026601	0.099393	1.901656	9.04E-09	5.34E-08
C7orf61	0.082224	0.234138	1.50972	8.06E-07	3.13E-06
TRIM71	0.367203	1.726536	2.23323	2.31E-07	1.01E-06
DEPDC1-AS1	0.011786	0.050631	2.102998	7.00E-13	1.06E-11
MAMSTR	0.358884	1.007344	1.488968	1.57E-16	5.42E-15
CHST10	0.490417	1.071407	1.127425	1.58E-07	7.17E-07
POLD1	3.112209	6.362936	1.031754	1.34E-26	1.09E-23
CCDC13-AS1	0.076484	0.407615	2.41397	9.57E-08	4.54E-07
CDHR1	0.043744	0.239714	2.454154	0.035163	0.048693
LINC00506	0.008877	0.03442	1.955158	3.93E-12	4.95E-11
PYCR1	4.537002	11.85411	1.385576	2.15E-07	9.44E-07
LINC02315	0.288636	1.115836	1.9508	5.87E-09	3.62E-08
ACTBP8	0.056151	0.826403	3.879456	1.49E-10	1.31E-09
CASC8	0.034521	0.163388	2.242773	2.54E-08	1.37E-07
DDIAS	0.278974	0.643238	1.20522	1.28E-18	7.36E-17
HGFAC	37.09467	15.85754	-1.22604	3.56E-08	1.85E-07
LINC01905	0.044935	0.161231	1.843201	3.20E-08	1.69E-07
UBE2S	3.479725	8.748256	1.330022	2.06E-26	1.39E-23

B3GNT9	0.789166	1.586529	1.007473	2.53E-07	1.10E-06
HLA-DQB2	2.273767	4.8744	1.10014	0.000189	0.000434
ZNF93	0.181636	0.564879	1.636891	3.00E-18	1.56E-16
GPX7	2.717237	6.044615	1.15351	4.33E-10	3.41E-09
ASF1B	2.3632	6.73192	1.510276	7.00E-24	1.45E-21
SAMD13	0.097117	0.21942	1.175895	3.02E-07	1.29E-06
B3GAT2	0.039465	0.084797	1.103451	2.03E-06	7.20E-06
GJD4	0.008701	0.017639	1.019547	0.002021	0.003722
OVOL3	0.028639	0.0816	1.51057	9.58E-08	4.54E-07
VN1R20P	0.024533	0.054843	1.160567	5.04E-06	1.65E-05
KHDRBS2	0.016767	0.043382	1.371521	0.001994	0.00368
RPS3AP13	0.019906	0.039933	1.004413	8.59E-06	2.66E-05
TERB2	0.349624	0.118029	-1.56666	4.60E-07	1.88E-06
POLD2P1	0.025127	0.141169	2.490145	3.40E-11	3.50E-10
STAC	0.149255	0.384146	1.363876	0.013193	0.02013
FCGR3A	7.300407	14.82407	1.021893	6.15E-05	0.000157
PCSK1N	2.461356	6.220069	1.337477	1.94E-05	5.55E-05
ZC3HAV1L	0.273898	0.598256	1.127127	1.50E-08	8.47E-08
CD80	0.090216	0.193148	1.09825	3.66E-06	1.23E-05
MCOLN3	0.159535	0.650383	2.027413	9.27E-08	4.41E-07
CTSK	5.275538	24.09948	2.191612	0.000165	0.000384
ITGA3	1.746152	3.78973	1.117916	0.002191	0.004005
CSTL1	0.037994	0.119423	1.652238	0.000106	0.000258
SHISAL2B	0.01723	0.03552	1.043752	0.000457	0.000966
ACTG1P20	0.152865	0.330666	1.113118	6.93E-07	2.72E-06
STARD6	0.004451	0.050913	3.515792	2.94E-13	4.81E-12
MLF1	0.218768	0.632546	1.531769	3.43E-06	1.16E-05
SF3A3P2	0.005238	0.011656	1.15408	9.84E-06	3.01E-05
DGKK	0.016612	0.130855	2.977666	0.021788	0.031639
PRKCG	0.007118	0.025849	1.860555	2.47E-06	8.61E-06
LINC00628	0.06908	0.141436	1.0338	3.44E-11	3.53E-10
LINC01748	0.042324	0.420402	3.312212	8.27E-21	7.50E-19
MRPS17P1	0.007745	0.021675	1.484687	1.91E-07	8.49E-07
NFIA-AS2	0.023987	0.049807	1.054128	0.000396	0.000849
CENPE	0.344662	0.942878	1.451889	2.42E-21	2.46E-19
IGHV4-39	10.48276	35.35952	1.75408	0.029429	0.041446
CDCA7	0.458096	2.170216	2.244118	4.82E-13	7.55E-12
MATN4	0.030784	0.70247	4.51218	0.000478	0.001005
CASC18	0.015046	0.039459	1.390984	0.002239	0.004084
TFPI2-DT	0.022354	0.125407	2.488026	2.87E-11	3.00E-10
LINC01224	0.092664	0.390592	2.075589	3.36E-13	5.44E-12
NPPB	0.147791	0.320923	1.118673	1.35E-08	7.68E-08
SMPD4P1	0.008266	0.043972	2.41136	0.000647	0.001325
LINC02576	0.027686	0.080948	1.547865	1.23E-07	5.69E-07

CR1L	0.026017	0.080462	1.628848	9.28E-08	4.41E-07
ARPC3P5	0.028731	0.070767	1.300497	2.09E-05	5.92E-05
SPON1-AS1	0.048339	0.127585	1.400204	0.000505	0.001057
CDC25C	0.905469	2.416606	1.416246	1.75E-21	1.86E-19
VN1R48P	0.090657	0.189244	1.061764	0.024996	0.035791
SOHLH1	0.029802	0.570692	4.259249	2.11E-06	7.48E-06
GAS2L3	0.518393	1.370508	1.402591	9.69E-22	1.11E-19
FAT2	0.010386	0.030184	1.539171	2.05E-05	5.83E-05
CXCL5	0.734835	6.165996	3.068841	3.79E-07	1.58E-06
TMIE	0.745146	1.68153	1.17418	0.000373	0.000804
NOC2LP2	0.019901	0.062855	1.659196	6.35E-07	2.52E-06
RCC2P6	0.009531	0.024994	1.390887	9.29E-07	3.55E-06
FOXN1	0.01265	0.032733	1.37162	0.000379	0.000816
LGI4	0.737108	1.724504	1.226234	1.01E-05	3.09E-05
BTN1A1	0.006221	0.025081	2.011452	6.51E-06	2.08E-05
SNHG3	1.706224	4.953058	1.537512	8.35E-20	6.23E-18
TNNC1	1.655887	3.580865	1.112704	0.011111	0.017252
ACTN3	0.012706	0.053998	2.087433	1.99E-06	7.10E-06
RPL7AP22	0.022641	0.046306	1.032243	0.000294	0.000646
ARHGEF19	0.643265	1.323312	1.040668	2.22E-13	3.76E-12
ELOVL4	0.070213	0.326842	2.218793	3.23E-08	1.70E-07
FAM72B	0.09079	0.260062	1.51826	3.16E-19	2.16E-17
MT1X	130.5439	54.50301	-1.26013	0.000521	0.001088
MXRA5Y	0.007933	0.015992	1.011424	0.011345	0.017567
TFAP2C	0.138325	0.285422	1.045036	0.012479	0.019137
TNIP3	0.034218	0.068499	1.001328	5.01E-07	2.04E-06
PKIB	1.292364	3.809961	1.559763	2.83E-06	9.72E-06
LAMA1	0.112892	0.297078	1.395893	0.032404	0.045265
CENPK	0.404706	1.001603	1.307363	2.38E-19	1.66E-17
THAP5P1	0.038818	0.086793	1.160851	5.46E-07	2.20E-06
CD3D	3.497994	7.182238	1.037905	1.12E-06	4.22E-06
PRAME	1.315537	4.833198	1.877326	8.26E-08	3.97E-07
CPSF4L	0.046112	0.111743	1.276964	0.000107	0.000258
TAFA4	0.201732	0.856195	2.0855	3.60E-05	9.66E-05
RAET1G	0.087744	0.177354	1.01526	0.000107	0.00026
NKPD1	0.021012	0.047088	1.164167	0.000123	0.000294
PLA2G1B	1.812987	3.734645	1.042603	0.003006	0.005329
PLPP4	0.185359	0.679932	1.875067	0.008347	0.01335
SGO1	0.438923	1.317729	1.586015	1.70E-25	6.90E-23
WDR38	0.015314	0.032919	1.104051	1.47E-06	5.37E-06
CENPW	4.368883	12.65873	1.534796	8.21E-28	1.39E-24
ZNF321P	0.163238	0.367234	1.169727	1.81E-14	4.01E-13
ZNF681	0.140236	0.443258	1.660288	9.21E-19	5.57E-17
RBM11	0.01099	0.068034	2.630006	0.00389	0.006713

PDCD1	0.799381	1.820129	1.187085	3.45E-06	1.16E-05
MAP1A	0.236818	0.601785	1.345468	0.004014	0.006905
BMP2K-DT	0.004492	0.00943	1.070044	1.91E-07	8.48E-07
CENPF	1.318063	3.403989	1.368807	5.53E-18	2.74E-16
LINC01991	0.012023	0.04092	1.76703	8.70E-05	0.000215
STIL	0.400595	1.00674	1.329475	3.96E-22	4.85E-20
RN7SL221P	0.128967	0.293852	1.188087	8.10E-11	7.61E-10
LINC02870	0.043167	0.588567	3.769198	1.62E-06	5.88E-06
LINC00634	0.031371	0.139112	2.148733	7.04E-19	4.44E-17
G6PD	5.464078	21.86105	2.000313	1.91E-22	2.48E-20
ACOXL	0.016912	0.033924	1.00429	8.89E-09	5.27E-08
PNCK	0.179317	1.717188	3.259465	1.25E-12	1.78E-11
IGFN1	0.024146	1.206818	5.643265	0.030634	0.04299
PARAL1	0.045118	0.150925	1.742048	6.32E-08	3.13E-07
OSM	0.234363	0.519552	1.148523	7.15E-06	2.26E-05
KPNA4P1	0.126104	0.05783	-1.12473	1.70E-05	4.91E-05
UGT3A2	0.386006	1.453101	1.912439	0.000418	0.000891
MAD2L1	0.907544	2.242708	1.305202	3.54E-21	3.47E-19
C5orf34	0.359549	0.817639	1.185276	6.53E-22	7.71E-20
MKI67	1.504396	4.103124	1.447539	1.17E-21	1.28E-19
LINC02820	0.073894	0.424772	2.523156	4.47E-15	1.12E-13
YJEFN3	0.435924	1.010079	1.212319	5.44E-11	5.30E-10
CIP2A	0.412782	1.287459	1.641076	5.92E-24	1.25E-21
TSSC2	0.065472	0.161366	1.301386	8.82E-05	0.000217
IGHV3-47	0.083643	0.293088	1.809027	1.69E-06	6.12E-06
BEST1	0.1701	0.355575	1.063769	2.71E-09	1.80E-08
CDC25B	7.985671	18.51437	1.21316	4.54E-21	4.37E-19
CASP5	0.043194	0.1339	1.632238	4.27E-09	2.71E-08
IL1A	0.012843	0.037167	1.5331	1.16E-09	8.35E-09
HPDL	0.143507	0.559504	1.963031	3.10E-11	3.23E-10
CENPI	0.304275	0.817989	1.426707	8.66E-22	9.99E-20
CD8B	0.59773	1.422305	1.250666	0.000226	0.000509
RXFP4	0.017477	0.102646	2.55416	6.23E-09	3.82E-08
HOXB7	0.733934	1.627265	1.148727	0.029694	0.041792
TPTE2	0.006267	0.012975	1.049939	0.000258	0.000573
UNC80	0.005018	0.010867	1.114784	0.004469	0.007608
FFAR3	0.013697	0.02765	1.013385	0.000378	0.000815
STK33	0.051513	0.122109	1.245149	7.54E-07	2.94E-06
LYPD8	0.313156	0.980858	1.647161	4.09E-08	2.11E-07
RPL23AP95	0.059417	0.135695	1.19141	1.46E-08	8.25E-08
TDRD9	0.057179	0.379903	2.732064	3.67E-05	9.81E-05
CSAG1	1.345017	8.056968	2.582613	6.28E-11	6.01E-10
SFRP5	4.475942	10.58137	1.241263	0.026885	0.038226
ADAM23	0.310445	0.991254	1.674916	8.53E-05	0.000211

ENO2	0.934741	2.310411	1.30551	3.31E-11	3.43E-10
HOXC9	0.282814	0.710713	1.329414	8.49E-05	0.00021
SOX11	0.017219	0.047365	1.459827	7.47E-07	2.92E-06
DLX2	0.024866	0.130889	2.396107	7.12E-09	4.31E-08
NDC80	1.33188	3.550292	1.414474	1.30E-22	1.83E-20
ETNPPL	30.45919	12.74562	-1.25688	2.49E-10	2.06E-09
F9	90.48367	43.0822	-1.07057	4.37E-12	5.45E-11
GRM6	0.011424	0.049842	2.125276	8.62E-05	0.000213
SCAT2	0.295977	0.692905	1.227173	5.73E-16	1.74E-14
PPP1R26P1	0.008666	0.020423	1.236767	0.012013	0.018486
TRAIP	0.667367	1.900241	1.50963	7.68E-29	2.23E-25
NLRP7	0.009137	0.032196	1.817065	5.09E-05	0.000132
LINC02550	0.092198	0.258372	1.486644	7.86E-07	3.06E-06
PTTG1	4.762384	13.49539	1.502711	1.83E-23	3.38E-21
GBX2	0.01328	0.03257	1.294326	0.000892	0.00177
FAM72D	0.095258	0.284558	1.578809	1.14E-19	8.38E-18
MED28P7	0.04637	0.133763	1.528402	6.22E-11	5.97E-10
PODNL1	0.112066	0.231966	1.049566	0.012712	0.01946
PCDHA3	0.025389	0.05802	1.192374	0.022401	0.032445
KIF25-AS1	0.017203	0.132154	2.941467	0.00083	0.001657
TNIK	0.626541	1.264881	1.01352	9.60E-07	3.66E-06
FAM83A-AS1	11.69591	1.961996	-2.57561	7.73E-08	3.75E-07
ZNF702P	0.142367	0.361603	1.344796	2.65E-07	1.14E-06
SNRK-AS1	0.02637	0.07128	1.434613	9.79E-07	3.73E-06
ATP8B5P	0.037012	0.100182	1.436583	8.24E-05	0.000205
CHODL	0.204771	0.613034	1.581956	0.00015	0.000351
TAGLN3	0.008657	0.03389	1.968904	0.002662	0.004779
CYCSP10	0.091728	0.190068	1.051083	2.39E-08	1.29E-07
OR7E126P	0.047839	0.144445	1.594251	1.89E-09	1.30E-08
GNAS-AS1	0.018381	0.051382	1.483062	0.000192	0.00044
IGHG3	19.99535	50.92665	1.348756	0.010024	0.015716
GPR19	0.124821	0.289565	1.214025	1.16E-15	3.35E-14
CDCA4	1.736944	3.996923	1.202338	1.66E-24	4.32E-22
HROB	0.652836	1.617042	1.308564	9.21E-23	1.35E-20
PCAT14	0.010841	0.068811	2.666109	0.003562	0.006205
ACAN	0.092697	0.293703	1.663756	3.61E-09	2.33E-08
NAA11	0.053227	0.760966	3.837607	1.64E-12	2.25E-11
CHI3L2	0.138693	0.487841	1.814515	7.33E-05	0.000184
NUDT11	0.049623	0.320054	2.689232	0.000403	0.000862
NLGN4X	0.320526	0.925747	1.530177	0.005086	0.008526
PHF10P1	0.01607	0.032199	1.002641	0.006021	0.009929
WDHD1	0.448598	1.097922	1.291281	1.09E-21	1.22E-19
PRPH2	0.063347	0.155069	1.291567	0.004174	0.007156
UBE2SP2	0.051235	0.118934	1.214954	4.88E-14	9.58E-13

PPIAP39	0.180759	0.415015	1.199093	0.01484	0.022383
DES	0.438498	2.280266	2.378561	0.024786	0.035504
TRPM2-AS	0.028546	0.072969	1.354004	0.003135	0.005531
LINC00944	0.28964	0.610932	1.076752	1.40E-07	6.42E-07
HOXA5	0.435575	1.054226	1.27519	0.000185	0.000425
ZYG11A	0.273216	0.577404	1.079538	3.46E-08	1.81E-07
DUSP9	2.659912	19.55008	2.877724	4.92E-12	6.08E-11
LINC01410	0.056043	0.143825	1.3597	0.000667	0.001361
KIF18A	0.316456	1.016613	1.683695	3.15E-23	5.25E-21
MAFA-AS1	0.845192	2.712322	1.682178	4.92E-06	1.61E-05
LINC02899	0.154595	0.390025	1.335073	0.001827	0.003399
KBTBD11-AS1	0.264773	0.108983	-1.28065	1.36E-07	6.24E-07
RPL7P38	0.01509	0.033386	1.145668	0.002124	0.003889
TYRP1	0.095138	0.445326	2.226773	0.000108	0.000262
ABCB5	0.035107	0.330039	3.232815	4.29E-06	1.42E-05
ACNATP	0.742412	0.362602	-1.03383	1.04E-09	7.59E-09
A4GNT	0.180057	0.493039	1.453248	6.61E-06	2.11E-05
KCNC1	0.018218	0.05652	1.633422	0.000242	0.000542
NDP	0.017037	0.050407	1.564966	3.63E-05	9.71E-05
FAM182B	0.037891	0.123088	1.699757	8.14E-15	1.93E-13
ARMCX1	1.037261	2.361881	1.187158	4.67E-06	1.53E-05
LHFPL3	0.025058	0.216097	3.108362	1.37E-13	2.43E-12
LINC00665	0.720048	2.094303	1.540304	1.21E-12	1.73E-11
SP8	0.060784	0.270056	2.151503	7.38E-08	3.59E-07
RN7SL8P	0.114213	0.65369	2.516884	2.36E-13	3.96E-12
ADAMTS14	0.158184	0.38854	1.296456	4.47E-07	1.83E-06
MMP14	15.12166	31.29577	1.049351	6.07E-09	3.73E-08
L1TD1	0.009844	0.056771	2.527829	0.003361	0.005888
TWIST1	0.112322	0.850433	2.920562	2.26E-06	7.95E-06
TFAP2E	0.132796	0.300084	1.176155	1.36E-11	1.53E-10
RPRM	0.014813	0.116559	2.976148	1.02E-10	9.33E-10
NAP1L6P	0.062957	0.275128	2.12766	2.90E-09	1.91E-08
PTK7	1.133618	2.564457	1.177718	1.03E-05	3.13E-05
FAR2P4	0.017866	0.135968	2.928004	1.19E-11	1.35E-10
PHEX	0.069388	0.270533	1.963038	3.89E-07	1.62E-06
ZFP69B	0.270885	0.546557	1.012693	4.97E-20	3.81E-18
NKX3-2	0.02974	0.161362	2.439802	6.93E-13	1.05E-11
CKS2	18.91227	42.63503	1.172717	9.81E-21	8.59E-19
ENPP6	0.16946	0.469435	1.469982	1.44E-05	4.25E-05
ICAM5	0.08836	0.221763	1.327553	0.000205	0.000468
ELOVL3	0.098647	0.421543	2.095334	8.59E-11	8.01E-10
RECQL4	3.330542	7.37539	1.146962	4.29E-17	1.68E-15
IGHV3-79	0.047738	0.449786	3.236026	1.48E-05	4.35E-05
KASH5	0.03373	0.100024	1.568228	2.22E-05	6.26E-05

GXYLT2	0.237623	0.597281	1.329732	0.000151	0.000353
HAS2-AS1	0.029009	0.08273	1.511915	0.000521	0.001087
FSCN1	6.967716	14.13332	1.020343	0.001345	0.002576
DUSP15	0.105663	0.425103	2.008344	6.24E-08	3.09E-07
KIF4A	1.357063	4.266962	1.652722	1.35E-23	2.64E-21
CLEC2L	0.043147	0.978782	4.503668	5.87E-16	1.78E-14
PTPRG-AS1	0.036986	0.089843	1.280408	2.61E-06	9.07E-06
E2F7	0.206919	0.502393	1.279747	8.87E-16	2.62E-14
CRLF1	0.385878	1.598522	2.050523	0.000141	0.000332
RPS12P31	0.035601	0.075118	1.077223	3.53E-06	1.19E-05
CPNE4	0.009221	0.025601	1.473223	2.72E-06	9.40E-06
KIF23	0.584155	1.812931	1.633902	1.32E-23	2.61E-21
UNC13C	0.001733	0.009882	2.51178	0.000285	0.000629
CDK1	2.243423	6.38669	1.509366	1.01E-23	2.00E-21
ZNF724	0.085082	0.184837	1.119327	2.51E-12	3.31E-11
BUB1	0.803456	2.235443	1.47627	1.61E-22	2.16E-20
SLCO4A1-AS1	0.165454	0.369984	1.161032	0.027772	0.039352
DCAF8L2	0.088865	0.187201	1.074904	8.44E-08	4.05E-07
CIT	0.389863	0.874192	1.164981	3.24E-17	1.32E-15
NAV2-AS3	0.035356	0.090953	1.363172	1.64E-05	4.77E-05
HASPIN	0.190114	0.474349	1.319079	7.89E-18	3.74E-16
GPM6B	0.082782	0.208877	1.335264	1.04E-06	3.93E-06
KRT15	0.052095	0.122743	1.236416	0.00257	0.004627
CXCL1	5.114859	12.55405	1.295386	0.000186	0.000428
GBP5	1.269765	2.629034	1.049971	0.001227	0.00237
REG4	0.076857	0.622895	3.018735	0.020178	0.029517
DSG1	2.255517	1.074491	-1.06981	5.49E-09	3.41E-08
ALDOAP2	0.010077	0.023772	1.238121	9.76E-08	4.61E-07
FCGR1A	0.358768	0.910377	1.343411	1.40E-06	5.15E-06
SGCG	0.00684	0.059587	3.122911	6.99E-06	2.21E-05
ZNF365	0.012116	0.029261	1.272022	0.000911	0.001804
PTGES3L	0.041252	0.141037	1.773547	4.43E-15	1.11E-13
CCR5AS	0.18678	0.400748	1.101357	1.63E-05	4.73E-05
LINC01589	0.177038	0.901224	2.347827	9.31E-08	4.42E-07
XCL1	0.300173	0.992222	1.72487	5.72E-12	6.98E-11
TLX2	0.02766	0.069932	1.338168	0.003394	0.00594
MAP7D2	0.591531	1.28008	1.113707	6.51E-05	0.000166
KCNB1	1.155758	0.536458	-1.1073	7.95E-08	3.84E-07
RRM2	3.770856	8.919623	1.242091	1.01E-21	1.14E-19
CENPA	0.851311	2.801207	1.71829	7.72E-26	3.48E-23
DPEP1	0.619719	1.282341	1.049094	0.034367	0.047737
ZNF208	0.02539	0.135271	2.413549	5.19E-07	2.10E-06
DLGAP1	0.022726	0.057861	1.34823	0.000113	0.000272
SLC22A20P	0.049512	0.102898	1.055367	1.48E-07	6.74E-07

LINC01667	0.134074	1.247984	3.218504	1.18E-11	1.33E-10
PRSS30P	0.061451	0.142991	1.218417	2.75E-05	7.58E-05
AURKAP1	0.016267	0.041602	1.354711	1.87E-07	8.33E-07
RNU4ATAC	0.231818	0.579506	1.321827	8.91E-06	2.75E-05
IGDCC4	0.116552	0.302289	1.374954	3.09E-07	1.31E-06
ANKRD18B	0.021248	0.055896	1.395443	2.83E-06	9.72E-06
SLC13A5	36.62062	18.29658	-1.00108	2.58E-13	4.29E-12
LINC01366	0.018764	0.045947	1.292009	0.000125	0.000298
NDN	2.235526	7.288791	1.705065	0.000112	0.00027
LINC01595	8.934277	3.877024	-1.2044	1.37E-06	5.06E-06
DENND5B-AS1	0.021444	0.048589	1.180042	5.89E-10	4.52E-09
LRP4	0.286906	0.747457	1.381414	2.56E-07	1.11E-06
LINC01711	0.025025	0.09672	1.950435	0.000449	0.00095
DRD1	0.442787	0.188357	-1.23315	1.20E-07	5.57E-07
SMPDL3B	0.448931	1.103768	1.297871	5.39E-11	5.26E-10
ZNF525	0.325165	0.658879	1.018841	2.03E-12	2.72E-11
PKD1L2	0.072516	0.220852	1.60671	0.001681	0.003157
CIB2	0.826666	2.117845	1.35722	5.76E-12	7.01E-11
GRIK1	0.033839	0.104788	1.630725	1.70E-07	7.66E-07
PTGFR	1.065057	2.485194	1.222427	0.003726	0.006461
FCGR2C	0.250429	0.671724	1.423468	6.96E-06	2.20E-05
MT3	0.335528	0.684731	1.029104	0.000255	0.000568
MCRIP2P1	0.062882	0.129171	1.038552	7.56E-07	2.95E-06
ADAMTS3	0.062186	0.177729	1.515015	0.000193	0.000443
ASCL2	0.248945	0.652746	1.390695	2.83E-06	9.72E-06
GAREM2	0.316299	0.681354	1.107115	1.40E-10	1.24E-09
LINC02109	0.025185	0.138705	2.461395	6.56E-11	6.25E-10
RBMXP1	0.038435	0.107526	1.484191	0.002089	0.003835
MIR663AHG	0.009209	0.138423	3.909828	6.32E-13	9.67E-12
ANXA8L1	0.025287	0.147787	2.547028	0.016472	0.024605
SLC7A8	0.767427	1.699268	1.146811	0.000302	0.000661
CALN1	0.00481	0.028792	2.581629	2.05E-05	5.81E-05
LRP2	0.058831	0.318466	2.436502	0.000151	0.000355
SFN	15.13402	36.10703	1.254484	1.78E-06	6.42E-06
RAB42	0.416868	1.168199	1.486625	1.53E-15	4.27E-14
HORMAD1	0.031123	0.075982	1.287685	0.019718	0.028918
GCNT1	0.246695	0.768387	1.639102	4.08E-05	0.000108
EEF1DP3	0.018824	0.045133	1.261602	6.00E-07	2.39E-06
H1-5	0.079159	0.167411	1.080576	1.85E-10	1.60E-09
FHOD3	0.176426	0.617431	1.807218	5.55E-09	3.44E-08
LDOC1	2.69741	7.089198	1.394048	0.015641	0.023472
LINC01775	0.064629	0.144636	1.162178	4.02E-09	2.56E-08
ZNF287	0.145508	0.319766	1.135924	3.11E-08	1.64E-07
HMGB1P3	0.042678	0.10302	1.271359	2.78E-05	7.65E-05

TMC2	0.008773	0.027097	1.626951	1.87E-07	8.33E-07
SLC22A16	0.011833	0.025696	1.11874	0.000187	0.000429
TUBB3	0.06734	0.154025	1.19364	0.000231	0.000519
SMIM43	0.017861	0.142746	2.998537	3.31E-09	2.16E-08
FUNDC2P2	0.10639	0.343556	1.691187	0.000524	0.001093
TMEM243	0.964066	2.004227	1.055842	6.27E-13	9.61E-12
ICOS	0.187029	0.384503	1.039735	6.22E-06	1.99E-05
HAPLN1	0.03273	0.25893	2.983891	1.56E-08	8.78E-08
HAPLN3	0.699792	1.417929	1.018788	1.58E-05	4.60E-05
RDH16	58.19368	28.32587	-1.03874	1.74E-08	9.71E-08
RGS13	0.017533	0.104368	2.573533	3.97E-08	2.05E-07
GABRG3	0.017258	0.041343	1.260398	0.004135	0.007095
POPDC3	0.189986	1.136637	2.580808	4.03E-09	2.57E-08
FAM153CP	0.007745	0.023519	1.602421	0.002556	0.004604
RPL10L	0.130236	0.792825	2.605876	4.33E-10	3.41E-09
COL24A1	0.042816	0.237334	2.47071	5.44E-11	5.30E-10
TPBG	0.318876	0.706529	1.147753	3.75E-05	0.0001
DCHS2	0.007179	0.023333	1.700474	3.00E-05	8.19E-05
CYP2E1	363.922	164.3708	-1.14668	9.94E-06	3.04E-05
LINC02506	1.058587	3.487729	1.720149	1.47E-07	6.70E-07
FOXD2	0.383925	0.78455	1.031043	1.41E-09	1.00E-08
KCTD17	3.017533	7.014195	1.216908	1.12E-13	2.04E-12
MROH7-TTC4	0.002752	0.005522	1.004926	0.001263	0.002433
ST6GALNAC5	0.029554	0.171336	2.535385	2.27E-15	6.08E-14
CCR10	0.149253	0.29937	1.004173	1.39E-12	1.95E-11
CYP2A13	3.433299	1.202794	-1.51321	0.000603	0.001242
ANKRD18CP	0.0174	0.042131	1.275818	7.64E-06	2.40E-05
ARL4AP5	0.016529	0.04147	1.327082	4.95E-07	2.01E-06
ADAM12	0.256054	0.533818	1.0599	2.57E-06	8.94E-06
SPDEF	0.824619	2.091929	1.343034	4.93E-05	0.000129
BSG-AS1	0.891518	1.936241	1.118922	4.89E-12	6.05E-11
GAP43	0.063182	0.238845	1.9185	5.78E-08	2.88E-07
HOXA11	0.049851	0.275893	2.468412	3.02E-08	1.60E-07
LINC01929	0.051245	0.20157	1.975813	0.000307	0.000673
ABHD17AP4	0.015507	0.049813	1.683607	0.00696	0.011312
DLGAP2	0.021673	0.007969	-1.44338	0.034656	0.04808
AIM2	0.187348	0.575955	1.620236	7.98E-05	0.000199
HOXD-AS2	0.110034	0.250664	1.187802	0.014924	0.022496
GPR89P	0.012892	0.057029	2.145205	1.27E-08	7.27E-08
CYP2C8	135.3875	66.22639	-1.03162	3.90E-09	2.50E-08
LDC1P	0.036469	0.073448	1.010046	0.000535	0.001114
TRPM2	0.466929	0.941539	1.011816	1.89E-07	8.42E-07
ERICH4	0.048324	0.235176	2.282921	1.27E-05	3.80E-05
LHX2	0.236299	0.733617	1.634412	0.000527	0.001098

SLC6A2	2.667405	1.117976	-1.25455	0.011595	0.017905
PITX1	0.829373	2.677064	1.690558	1.03E-06	3.89E-06
AGPAT4	0.326524	0.818053	1.325003	6.98E-13	1.06E-11
SLC30A3	0.214118	0.562853	1.394353	0.003149	0.005555
LINC02377	0.174976	0.608478	1.798045	1.68E-08	9.39E-08
MIR646HG	0.051784	0.109991	1.086821	5.07E-05	0.000132
ANO4	0.049353	0.128071	1.375736	0.000231	0.00052
MCM4	4.839172	10.40528	1.104484	2.13E-18	1.15E-16
PLK4	0.380729	1.007554	1.404021	1.96E-22	2.54E-20
MUC12-AS1	0.171101	1.082816	2.661866	1.97E-05	5.62E-05
TBX3-AS1	1.120349	0.548353	-1.03077	8.57E-06	2.66E-05
UGT1A2P	4.212259	1.506999	-1.48292	3.64E-08	1.89E-07
AXDND1	0.021861	0.061037	1.481289	3.75E-05	0.0001
POU4F1	0.008801	0.034751	1.981276	0.000958	0.001889
MGAM2	0.093542	0.527389	2.49518	2.63E-07	1.13E-06
NCAPD2	3.079216	7.045631	1.194166	2.08E-21	2.15E-19
SCG3	0.033789	0.187549	2.472628	9.14E-07	3.50E-06
GABRA3	0.044869	0.495864	3.466168	2.75E-17	1.14E-15
FANCB	0.084215	0.236456	1.48943	1.77E-22	2.35E-20
CCIN	0.019225	0.039672	1.04515	0.004059	0.006975
NT5DC2	2.958429	9.587597	1.696338	2.55E-17	1.08E-15

Table S6 Co-expression miRNA of P3H1 obtained using starBase

miRNAname	Gene Name	clipExp Num	PITA	RNA22	miRmap	microT	miRanda	PicTar	Target Scan	Pancancer Num
hsa-miR-29a-3p	P3H1	13	1	0	1	1	1	0	1	20
hsa-miR-29b-3p	P3H1	13	1	0	1	1	1	0	1	23
hsa-miR-29c-3p	P3H1	13	1	0	1	1	1	0	1	26
hsa-miR-362-5p	P3H1	1	1	0	0	0	0	0	1	11
hsa-miR-370-3p	P3H1	2	1	0	0	1	0	0	0	1
hsa-miR-335-5p	P3H1	13	1	0	0	0	1	0	0	9
hsa-miR-588	P3H1	1	1	1	0	0	0	0	0	0
hsa-miR-500b-5p	P3H1	2	0	0	0	1	0	1	1	4
hsa-miR-378g	P3H1	2	0	1	1	1	0	0	0	4
hsa-miR-378g	P3H1	2	0	0	1	1	0	0	0	4
hsa-miR-378g	P3H1	2	0	0	1	1	0	0	0	4
hsa-miR-1343-3p	P3H1	2	0	0	1	0	0	1	0	2

Table S7 Co-expression lncRNA of hsa-miR-29c-3p obtained using starBase

miRNAname	geneName	geneType	clipExpNum	pancancerNum
hsa-miR-29c-3p	AL139287.1	sense_intronic	1	4
hsa-miR-29c-3p	AL391244.1	processed_transcript	7	5
hsa-miR-29c-3p	AL031282.2	processed_transcript	1	2
hsa-miR-29c-3p	AL513327.3	lincRNA	1	4
hsa-miR-29c-3p	AL138787.2	antisense	1	3
hsa-miR-29c-3p	AL138787.2	antisense	1	3
hsa-miR-29c-3p	AL603840.1	lincRNA	1	4
hsa-miR-29c-3p	AL355488.1	antisense	8	7
hsa-miR-29c-3p	AL365361.1	lincRNA	1	0
hsa-miR-29c-3p	RP11-435B5.4	lincRNA	1	0
hsa-miR-29c-3p	GAS5	processed_transcript	4	5
hsa-miR-29c-3p	GAS5	processed_transcript	4	5
hsa-miR-29c-3p	GAS5	processed_transcript	4	5
hsa-miR-29c-3p	MIR29B2CHG	lincRNA	1	0
hsa-miR-29c-3p	AC098828.2	lincRNA	1	12
hsa-miR-29c-3p	DNAJC27-AS1	antisense	1	3
hsa-miR-29c-3p	AC132154.1	TEC	1	3
hsa-miR-29c-3p	PCBP1-AS1	processed_transcript	1	2
hsa-miR-29c-3p	AC010894.2	lincRNA	1	6
hsa-miR-29c-3p	AC016717.2	lincRNA	4	3
hsa-miR-29c-3p	LINC01907	lincRNA	1	3
hsa-miR-29c-3p	THUMPD3-AS1	antisense	3	7
hsa-miR-29c-3p	LINC00852	antisense	3	4
hsa-miR-29c-3p	LINC00879	lincRNA	1	4
hsa-miR-29c-3p	NOP14-AS1	antisense	1	7
hsa-miR-29c-3p	MIR4458HG	lincRNA	6	0
hsa-miR-29c-3p	LIFR-AS1	antisense	1	2
hsa-miR-29c-3p	HCG18	antisense	2	9
hsa-miR-29c-3p	HCP5	sense_overlapping	3	1
hsa-miR-29c-3p	AFDN-DT	lincRNA	2	9
hsa-miR-29c-3p	AC091729.3	antisense	1	6
hsa-miR-29c-3p	AC019117.2	lincRNA	1	4
hsa-miR-29c-3p	HOXA-AS3	antisense	1	8
hsa-miR-29c-3p	HOXA10-AS	antisense	2	9
hsa-miR-29c-3p	AC007036.3	sense_overlapping	9	0
hsa-miR-29c-3p	AC005154.1	processed_transcript	3	5
hsa-miR-29c-3p	AC005154.1	processed_transcript	4	5
hsa-miR-29c-3p	AC005154.1	processed_transcript	2	5
hsa-miR-29c-3p	AC018647.2	antisense	1	5
hsa-miR-29c-3p	SNHG15	lincRNA	1	7
hsa-miR-29c-3p	AC073335.2	lincRNA	1	2

hsa-miR-29c-3p	STAG3L5P-PVRIG2P-PILRB	processed_transcript	4	3
hsa-miR-29c-3p	LINC00689	processed_transcript	1	6
hsa-miR-29c-3p	AC104964.4	lincRNA	1	3
hsa-miR-29c-3p	AC091182.1	processed_transcript	1	5
hsa-miR-29c-3p	AC083837.1	lincRNA	1	1
hsa-miR-29c-3p	AC083837.1	lincRNA	1	1
hsa-miR-29c-3p	AC090579.1	antisense	1	2
hsa-miR-29c-3p	PVT1	lincRNA	2	8
hsa-miR-29c-3p	AC105219.1	sense_intronic	1	5
hsa-miR-29c-3p	AC084125.2	processed_transcript	1	4
hsa-miR-29c-3p	EBLN3P	lincRNA	4	4
hsa-miR-29c-3p	AL590705.5	processed_transcript	1	2
hsa-miR-29c-3p	ARRDC1-AS1	antisense	2	4
hsa-miR-29c-3p	AL157392.3	processed_transcript	1	4
hsa-miR-29c-3p	AL158835.1	lincRNA	1	2
hsa-miR-29c-3p	H19	processed_transcript	13	12
hsa-miR-29c-3p	KCNQ1OT1	antisense	1	7
hsa-miR-29c-3p	KCNQ1OT1	antisense	1	7
hsa-miR-29c-3p	KCNQ1OT1	antisense	2	7
hsa-miR-29c-3p	KCNQ1OT1	antisense	2	7
hsa-miR-29c-3p	AL137804.1	antisense	1	5
hsa-miR-29c-3p	NEAT1	lincRNA	14	1
hsa-miR-29c-3p	NEAT1	lincRNA	9	1
hsa-miR-29c-3p	NEAT1	lincRNA	9	1
hsa-miR-29c-3p	NEAT1	lincRNA	10	1
hsa-miR-29c-3p	NEAT1	lincRNA	12	1
hsa-miR-29c-3p	NEAT1	lincRNA	8	1
hsa-miR-29c-3p	SSSCA1-AS1	processed_transcript	2	3
hsa-miR-29c-3p	AP003555.1	lincRNA	1	2
hsa-miR-29c-3p	AP000873.2	processed_transcript	1	3
hsa-miR-29c-3p	AP001273.1	TEC	1	4
hsa-miR-29c-3p	AP003025.1	antisense	1	5
hsa-miR-29c-3p	MIR4697HG	TEC	4	3
hsa-miR-29c-3p	AC092747.4	lincRNA	3	2
hsa-miR-29c-3p	AC144548.1	processed_transcript	2	5
hsa-miR-29c-3p	LINC00943	lincRNA	1	2
hsa-miR-29c-3p	AL355001.2	lincRNA	2	6
hsa-miR-29c-3p	AL135999.1	antisense	3	2
hsa-miR-29c-3p	AL137129.1	processed_transcript	11	4
hsa-miR-29c-3p	VASH1-AS1	lincRNA	2	4
hsa-miR-29c-3p	LINC00638	lincRNA	1	10
hsa-miR-29c-3p	FAM30A	lincRNA	1	2
hsa-miR-29c-3p	RAD51-AS1	processed_transcript	1	3

hsa-miR-29c-3p	OIP5-AS1	processed_transcript	7	3
hsa-miR-29c-3p	DNAAF4-CCPG1	processed_transcript	3	0
hsa-miR-29c-3p	NPTN-IT1	sense_intronic	1	2
hsa-miR-29c-3p	NPTN-IT1	sense_intronic	1	2
hsa-miR-29c-3p	LINC01578	processed_transcript	1	5
hsa-miR-29c-3p	AC022167.2	antisense	1	3
hsa-miR-29c-3p	MIR193BHG	lincRNA	1	9
hsa-miR-29c-3p	AC120114.4	TEC	1	3
hsa-miR-29c-3p	MIR762HG	antisense	1	3
hsa-miR-29c-3p	CRNDE	lincRNA	1	9
hsa-miR-29c-3p	AC040162.3	lincRNA	3	2
hsa-miR-29c-3p	AC020978.7	sense_overlapping	2	2
hsa-miR-29c-3p	AC092384.3	sense_intronic	1	3
hsa-miR-29c-3p	AC012146.1	processed_transcript	5	8
hsa-miR-29c-3p	MIR497HG	antisense	5	1
hsa-miR-29c-3p	AC087501.4	sense_intronic	1	5
hsa-miR-29c-3p	CCDC144NL-AS1	antisense	1	6
hsa-miR-29c-3p	AC005899.4	processed_transcript	1	2
hsa-miR-29c-3p	AC018521.1	processed_transcript	7	1
hsa-miR-29c-3p	AC018628.1	TEC	3	4
hsa-miR-29c-3p	AC005332.7	lincRNA	4	5
hsa-miR-29c-3p	LINC00511	lincRNA	1	19
hsa-miR-29c-3p	SNHG20	processed_transcript	1	5
hsa-miR-29c-3p	AC114271.1	antisense	3	4
hsa-miR-29c-3p	AC092329.4	processed_transcript	1	0
hsa-miR-29c-3p	LINC01224	lincRNA	1	5
hsa-miR-29c-3p	AC002116.1	processed_transcript	1	3
hsa-miR-29c-3p	AC016590.3	antisense	1	3
hsa-miR-29c-3p	CTD-2337J16.1	lincRNA	1	0
hsa-miR-29c-3p	SNHG17	processed_transcript	1	15
hsa-miR-29c-3p	LINC01270	lincRNA	1	10
hsa-miR-29c-3p	MIR646HG	lincRNA	1	3
hsa-miR-29c-3p	AL121832.3	sense_intronic	1	5
hsa-miR-29c-3p	AL117379.1	lincRNA	1	4
hsa-miR-29c-3p	AP001432.1	lincRNA	5	4
hsa-miR-29c-3p	AJ239328.1	sense_overlapping	1	3
hsa-miR-29c-3p	DUXAP8	processed_transcript	1	12
hsa-miR-29c-3p	DUXAP8	processed_transcript	3	12
hsa-miR-29c-3p	MIAT	lincRNA	1	2
hsa-miR-29c-3p	MIAT	lincRNA	1	2
hsa-miR-29c-3p	TUG1	bidirectional_promoter_lncRNA	14	10
hsa-miR-29c-3p	TUG1	bidirectional_promoter_lncRNA	7	10
hsa-miR-29c-3p	LINC01521	lincRNA	2	13
hsa-miR-29c-3p	AL031587.5	TEC	2	10

hsa-miR-29c-3p	MIRLET7BHG	lincRNA	13	3
hsa-miR-29c-3p	AL117329.1	lincRNA	1	8
hsa-miR-29c-3p	BX890604.1	lincRNA	1	8
hsa-miR-29c-3p	XIST	lincRNA	8	1
